# Integrative microbiome and metabolome profiles reveal the impacts of periodontitis via oral-gut axis in first-trimester pregnant women

**DOI:** 10.1186/s12967-024-05579-9

**Published:** 2024-09-03

**Authors:** Tianfan Cheng, Ping Wen, Rong Yu, Feng Zhang, Huijun Li, Xiaoyi Xu, Dan Zhao, Fang Liu, Weilan Su, Zheng Zheng, Hong Yang, Jilong Yao, Lijian Jin

**Affiliations:** 1 Division of Periodontology & Implant Dentistry, Faculty of Dentistry, The University of Hong Kong, Hong Kong, China; 2 Institute of Maternal and Child Medicine & Shenzhen Key Laboratory of Maternal and Child Health and Diseases, Shenzhen Maternity & Child Healthcare Hospital, Shenzhen, China; 3 Division of Stomatology, Shenzhen Maternity & Child Healthcare Hospital, Shenzhen, China; 4 Center for Disease Control and Prevention, Shenzhen, China; 5 Department of Implant Dentistry, Beijing Stomatological Hospital, Capital Medical University, Beijing, China; 6 Division of Obstetrics & Gynecology, Shenzhen Maternity & Child Healthcare Hospital, Shenzhen, China

**Keywords:** Periodontitis, Pregnancy, Microbiome, Metabolome, Gut, Integrative analysis, First trimester

## Abstract

**Background:**

Periodontitis results from host-microbe dysbiosis and the resultant dysregulated immunoinflammatory response. Importantly, it closely links to numerous systemic comorbidities, and perplexingly contributes to adverse pregnancy outcomes (APOs). Currently, there are limited studies on the distal consequences of periodontitis via oral-gut axis in pregnant women. This study investigated the integrative microbiome-metabolome profiles through multi-omics approaches in first-trimester pregnant women and explored the translational potentials.

**Methods:**

We collected samples of subgingival plaques, saliva, sera and stool from 54 Chinese pregnant women at the first trimester, including 31 maternal periodontitis (Perio) subjects and 23 Non-Perio controls. By integrating 16S rRNA sequencing, untargeted metabolomics and clinical traits, we explored the oral-gut microbial and metabolic connection resulting from periodontitis among early pregnant women.

**Results:**

We demonstrated a novel bacterial distinguisher *Coprococcus* from feces of periodontitis subjects in association with subgingival periodontopathogens, being different from other fecal genera in Lachnospiraceae family. The ratio of fecal *Coprococcus* to *Lachnoclostridium* could discriminate between Perio and Non-Perio groups as the ratio of subgingival *Porphyromonas* to *Rothia* did. Furthermore, there were differentially abundant fecal metabolic features pivotally enriched in periodontitis subjects like L-urobilin and kynurenic acid. We revealed a periodontitis-oriented integrative network cluster, which was centered with fecal *Coprococcus* and L-urobilin as well as serum triglyceride.

**Conclusions:**

The current findings about the notable influence of periodontitis on fecal microbiota and metabolites in first-trimester pregnant women via oral-gut axis signify the importance and translational implications of preconceptional oral/periodontal healthcare for enhancing maternal wellbeing.

**Supplementary Information:**

The online version contains supplementary material available at 10.1186/s12967-024-05579-9.

## Background

Periodontitis, a major oral disease burden worldwide, is the primary cause of severe tooth loss in adults, with huge socio-economic impacts [1]. Its onset and progression are crucially attributed to the dysbiotic biofilms on oral niches and dysregulated immunoinflammatory response [2]. The keystone pathogen *Porphyromonas gingivalis* is essential to drive the shift of symbiosis to dysbiosis, thereby disrupting microbe-host homeostasis and accounting for the pathogenesis of periodontitis [2]. Actually, periodontitis also closely links to its systemic comorbidities/disorders, e.g., diabetes, cardiovascular disease, inflammatory bowel disease, colorectal cancer, Alzheimer’s disease (AD) and adverse pregnancy outcomes (APOs), via the underlying pathways of infection, inflammation, maladaptation of stem cells and myelopoiesis, and life-style risk factors like tobacco use [2, 3]. The study on the oral-gut microbiota profiles has revealed the distal impacts of periodontitis [4].

For women, increased progesterone and estrogen levels during pregnancy may greatly increase the risk of gestational gingivitis and periodontitis [5]. As pregnancy progresses, there are apparent changes in gut and vaginal microbiota, metabolic and immune profiles [6, 7], and maternal periodontitis increases the risks of APOs like preterm birth/low birth weight [3] and accounts for abnormal liver function [8]. Actually, periodontal diseases induce high levels of childbirth-related proinflammatory cytokines, and periodontopathogens are detectable in placenta and amniotic fluid of pregnant women with periodontitis [3]. Evidently, maternal microbiome casts extensive effects on fetal/neonatal development and the establishment of normal microbiota following different delivery modes, breastfeeding or antibiotic usage [7], via multiple routes of amniotic fluid and placenta, vagina, feces, milk and saliva.

Although the remodeling of oral and gut microbiome and metabolome during pregnancy was studied separately [5, 9–11], currently, there are limited integrative multi-omics and multi-body-site studies on connecting pregnancy to other inter-linked diseases and conditions like periodontitis. We hypothesized that maternal periodontitis may exert distal impacts on fecal microbiota and metabolites in pregnant women via the oral-gut axis. This study investigated both microbiome and metabolome profiles among the first-trimester pregnant women with or without periodontitis and addressed the translational implications.

## Methods

### Study design and subjects

**Fig. 1:**
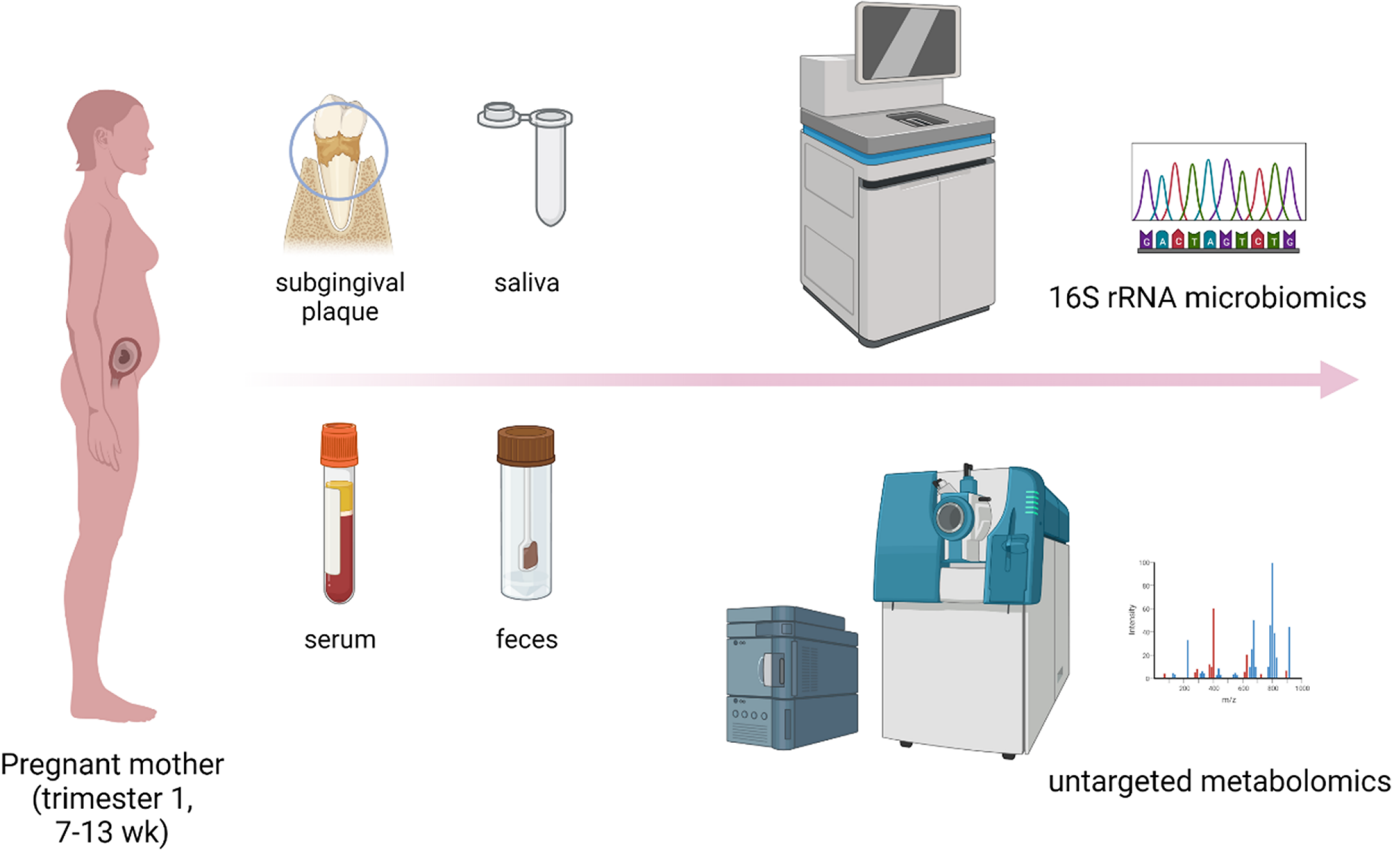
Study design and workflow of sampling and analysis. Microbiomic and metabolomic analyses were conducted on samples of subgingival plaque, saliva, serum, and feces from first-trimester pregnant women with different periodontal states (Created with Biorender.com)

The study was approved by the medical ethics committee of Shenzhen Maternity & Child Healthcare Hospital (SMCHH) (Approval No.: SFYLS [2020]013), following the current Declaration of Helsinki. A total of 54 Chinese pregnant women (20–35 years at the first trimester, 7–13 weeks of gestation) were recruited from SMCHH during the period of August 2020 to February 2021, with informed written consents from all participants. The details of recruitment, examination, and sample collection as well as demographic characteristics and systemic/periodontal profiles are presented in the following Methods and Tables S1–S4. The study framework is presented in Fig. 1. The following pregnant females were excluded from the present study: (a) pre-pregnant body mass index (BMI) ≥ 28 kg/m^2^ and systemic diseases/disorders; (b) infectious diseases including COVID-19; (c) non-singleton pregnancy; (d) use of antibiotics, immunosuppressants, anti-osteoporotic agents or steroids/hormones; (e) lack of medical files at SZMCH; (f) undertaking active orthodontic treatment; (g) periodontal therapy in preceding 3 months; and (h) vegetarian or vegan.

Details of clinical examination and collection of samples were described in Supplementary Materials.

### DNA extraction and 16S rRNA sequencing

Total genomic DNA was extracted in all kinds of samples with the CTAB method. The V3‒V4 regions of 16S rRNA were amplified by the barcoded pair of 341F (5’-CCTAYGGGRBGCASCAG-3’) and 806R (5’-GGACTACNNGGGTATCTAAT-3’) for library preparation. Two blank controls for each sample were included during amplification for verifying no contamination. Sequencing was undertaken on the Illumina NovaSeq 6000 Systems (Illumina) for PE250 (50,000 tags per sample) paired-end sequencing (Novogene, Beijing, China).

### Data processing and microbiome analysis

The raw datasets were analyzed with DADA2 v1.22.0 [12] pipeline for generating Amplicon Sequence Variant (ASV) counts, and taxonomy assignment was performed with curated train set with species of SILVA database v138.1 for DADA2. For microbiome analysis, singletons in data were filtered out as minimally filtered dataset (MFD) remaining 4,741 ASVs. Details were described in Supplementary Materials.

### Selection of microbial features

Differentially abundant taxa were selected from the dataset of minima of relative abundance of 0.05% and prevalence of 5% (FD0.05), implementing methods of DESeq2 [13], ANCOM-BC2 [14], sPLS-DA [15] and random forest [16]. Details were presented in Supplementary Materials.

### Functional prediction

Unstratified functional prediction was performed using PICRUSt2 2.5.0 [17] on the dataset FD0.05, and the predicted results of enzyme commissions (ECs), KEGG orthologs (KOs) and MetaCyc pathways (PWs) were further examined using ANCOM-BC2 (*P* < 0.05). Further Spearman’s correlation of DESeq2-selected genera (centered log-ratio (clr) transformed microbiome abundance) with ANCOM-BC2-selected functional terms was analyzed using *corr.test* function of psych v2.3.9 (FDR < 0.05).

### Metabolite extraction, separation, and mass spectrum acquisition

Details were described in Supplementary Materials.

### Untargeted metabolomic data pre-processing

Details were shown in Supplementary Materials.

### Metabolomic analysis

Metabolome data were further filtered with the criteria of the relative standard deviation (RSD) > 30% of QC samples and interquartile range (IQR) < 10%, and further log_10_-transformed and Pareto scaled using MetaboAnalyst 5.0 and its MetaboAnalystR v3.3.0 package [18], generating filtered datasets (FD-IQR-Pareto). PCA was performed using mixOmics and PERMANOVA on Euclidian distances between Non-Perio and Perio was conducted using *adonis2*. Significantly differentially abundant metabolites were identified using univariate approaches Wilcoxon rank-sum test and linear model of *limma* [19] incorporating BMI covariate implemented in MetaboAnalystR and using generalized linear model (GLM) incorporating BMI covariate using *glm* function of R package *stats* v4.2.2. sPLS-DA was utilized to explore metabolic features associated with Non-Perio and Perio. Selected significant metabolic features were further subjected to functional pathway enrichment with integration of *mummichog* (*p*-value cutoff: 0.01) and gene set enrichment analysis (GSEA) algorithms using the human MFN pathway library implemented in MetaboAnalystR. Selected significant metabolic features with MS2 annotation and HMDB identifiers were further subjected to quantitative metabolite enrichment analysis (qMSEA) with log_10_ transformation and Pareto scaling against 99 a priori defined metabolite sets in The Small Molecule Pathway Database (SMPDB) and 44 a priori sets in human feces for disease signatures using only metabolite sets containing at two compound entries.

### Integration of microbiome, metabolome, and systemic clinical parameters

The multi-omics integration algorithm DIABLO [20] of mixOmics was implemented to either integrate the microbiome and metabolome data from various sources (SBP, MSA and MST) or integrate the microbiome, metabolome and clinical data from same source (SBP and MST), for exploring the underlying features contributing to periodontal status. Microbiome data of FD0.05 with robust centered log-ratio (rclr) transformation of relative abundance (FD0.05-rclr) were used at the levels of ASV, genus and species. Metabolome data of FD-IQR-Pareto were used. By setting block link of the design matrix of 0.1 (correlation value), the initial DIABLO models (generated using *block.splsda* function) were tuned with folds/repeats (10 × 50) of cross-validation to select numbers of components and features in the final models using *tune.block.splsda* function. Microbiome data of FD0.05 and clr-transformed FD0.05 were both used in Spearman’s correlation analysis.

## Results

### Pregnant women with periodontitis had a notable microbiome profile at the first trimester

The 54 pregnant women (7 − 13 gestational weeks), 31 Periodontitis subjects (Perio group) and 23 Periodontal Health/Gingivitis subjects (Non-periodontitis/Non-Perio group) following the current AAP/EFP classification [21], were recruited respectively control of confounders (see Methods) minimizing their influences. There was no significant difference in gestational week when examining subjects and taking samples and demographic characteristics including dietary habitats in both groups. It existed significant increase in blood glucose and decrease of serum albumin in Perio; besides periodontal characteristics and no significant differences in demographic characteristics including dietary habitats (Fig. 1, full dataset: Table S1, summaries: Tables S2–S4). We noticed significantly increased weight at delivery, BMI at 34 − 36 gestational weeks and that at delivery in Perio group. PCA on systemic parameters showed significant inter-group variation only at Component 1 (18%, *P* = 0.0075, Fig. S1). 16S rRNA sequencing identified 4,741 ASVs across all samples of *s*u*b*gingival *p*laque (SBP), *sa*liva (*m*aternal, MSA) and *st*ool (*m*aternal, MST, using feces afterwards), after singleton removal. The multivariate framework MixMC well discriminated the microbiota of these three sample sources (Fig. S2). Similar apparent inter-group variations of community composition occurred at phylum, genus, and family in SBP and MST, notably increasing *Prevotella* in Perio (Table S7, Figs. S3 − S5). Periodontitis did not significantly alter the alpha diversity of microbiota for all sample types, except the Camargo’s Evenness is significantly lower in Perio (*P* = 0.0067). The PCoA on Bray-Curtis distances (Fig. S6) revealed significant variations among three sample types (PERMANOVA: *P* = 0.001), and subgingival inter-group variation (PERMANOVA: *P* = 0.004, Table S8).

### Maternal periodontitis accounted for microbial oral-gut variations in oral samples and feces

**Fig. 2:**
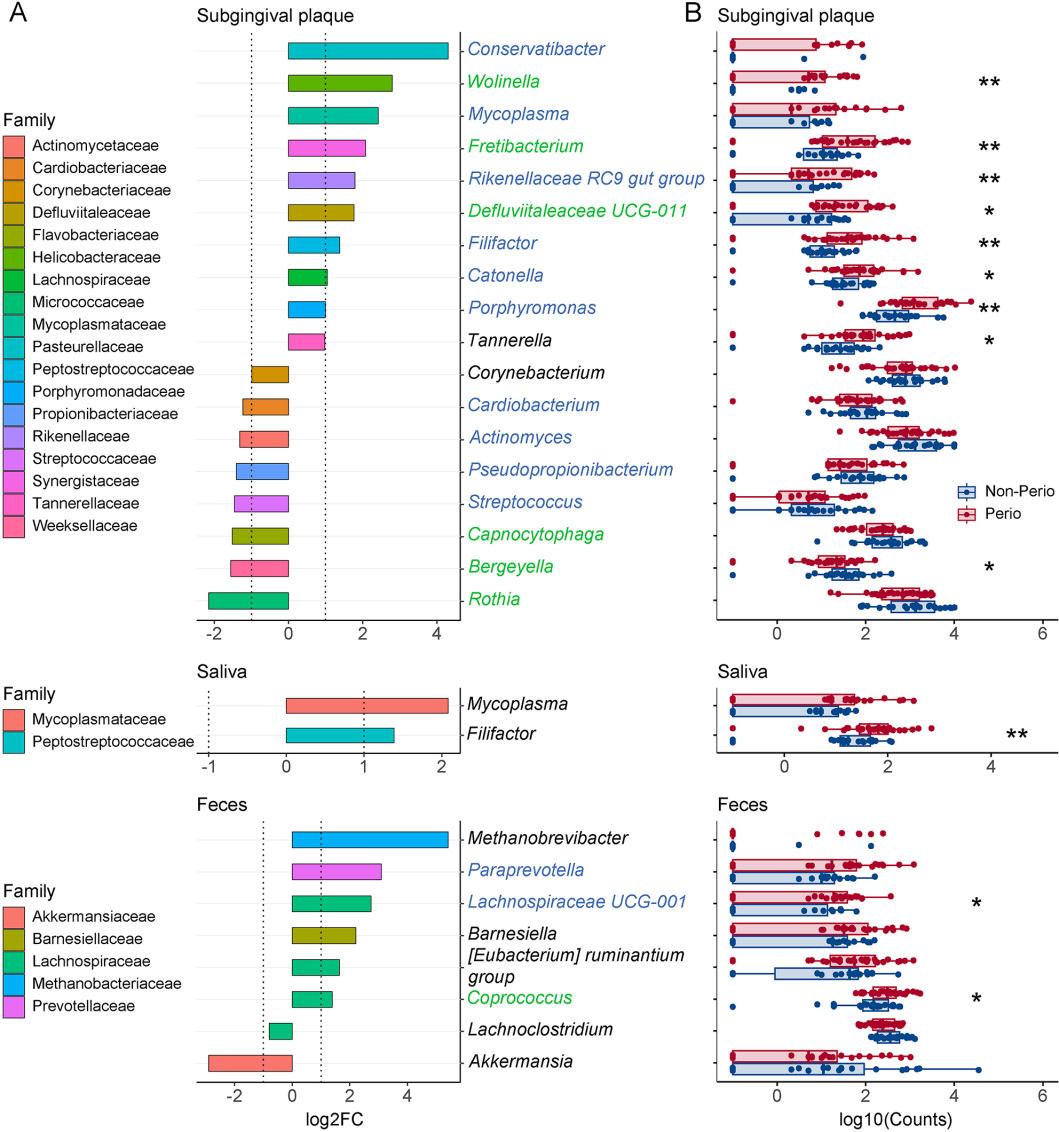
DESeq2-selected differentially abundant genera in subgingival plaques (SBP), saliva (MSA) and feces (MST) in the pregnant women with Perio (*n* = 31) and Non-Perio (*n* = 23). **A** Log_2_FC: Perio to Non-Perio. FDR-adjusted *P* values from Wald test (two-sided) implemented with Benjamini-Hochberg adjustment for multiple comparisons. Green labels of genera: FDR-adjusted *P* < 0.05; blue: FDR-adjusted *P* < 0.1. The detailed DESeq2 results are provided in Table S9. **B** Boxplots of selected genera abundance (counts) in log_10_ scale with addition of a pseudocount of 0.1 to the original counts. Wilcoxon rank sum test: *P < 0.05, **P < 0.01

For each sample type, we retained 1,175, 1,377 and 1,076 ASVs for SBP, MSA and MST respectively after filtering (relative abundance > 0.05%; prevalence > 5%). Considering the distal nature of gut microbiome to the oral cavity and the young and generally healthy subjects, we implemented multiple approaches to exploring periodontitis-discriminative (differentially abundant) microbial taxa, including DESeq2, ANCOM-BC2, sPLS-DA and random forest. Expectedly, the abundance of subgingival families, genera and species of several periodontopathogens [2] like *Porphyromonas*, *Tannerella*, *Filifactor*, *Wolinella* and *Fretibacterium* spp. significantly increased in Perio as demonstrated by DESeq2 (FDR < 0.05), Wilcoxon rank sum test and ANCOM-BC2 (insensitive to the pseudo-count addition); whereas there was a decrease in oral commensals or opportunistic pathogens such as *Rothia* and *Capnocytophaga* spp. (Fig. 2, Fig. S7, Table S9, S10A-C). In contrast, the taxa of common periodontopathogens in saliva were hardly identified using both DESeq2 and ANCOM-BC2, except species by ANCOM-BC2, indicating salivary microbiome is less powerful in discriminating periodontal status during pregnancy. Taxon features from classes of Bacteroidia and Clostridia in feces accounted mostly for discernment. In feces, the family and genus for the gut commensal *Akkermansia* were Non-Perio-enriched by DESeq2, while *Coprococcus* spp. and Lachnospiraceae UCG-001 from the Lachnospiraceae family accounted for Perio by both DESeq2 (FDR < 0.05) and ANCOM-BC2. Surprisingly, fecal genera, species and ASVs of Lachnospiraceae showed divergent favor with Perio, leading to no differential abundance in Lachnospiraceae family (Tables S9 and S10A).

**Fig. 3:**
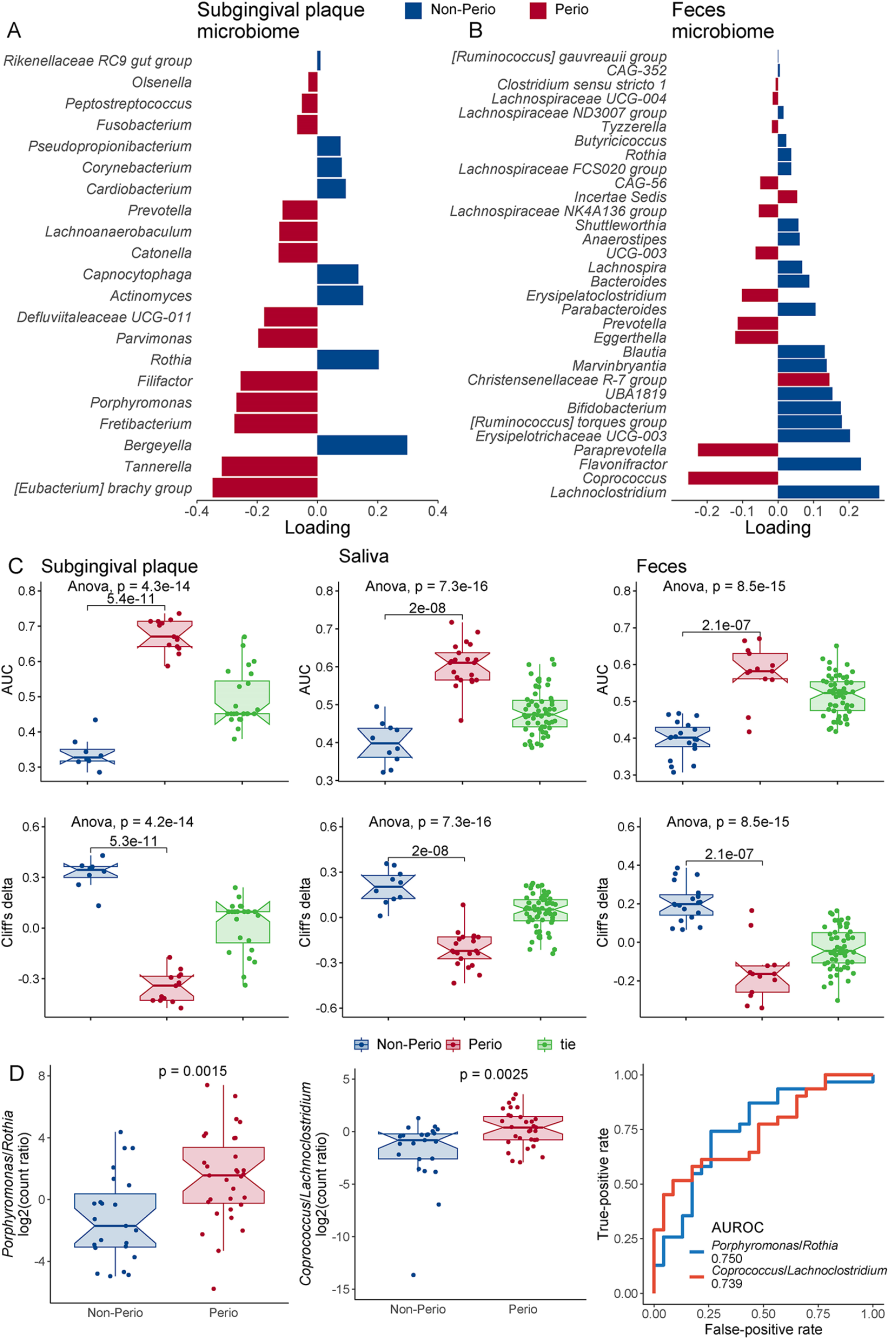
sPLS-DA analysis on microbial genera in the pregnant women with Perio (*n* = 31) and Non-Perio (*n* = 23). **A** sPLS-DA-selected important genera in subgingival plaques (SBP) in Component 1. Genera of “tie” (non-differentiated) of sPLS-DA were not shown. **B** sPLS-DA-selected important genera in feces (MST) in Component 1. Bar length represents loading coefficient weight of selected genera, ranked in ascending order of importance, bottom to top; bar color denotes the group in which the genus has the highest median abundance, red = Perio, blue = Non-Perio. The detailed sPLS-DA results are provided in Table S11. **C** Bar plots of AUROCs and Cliff’s deltas of sPLS-DA-selected genera in subgingival plaques, saliva and feces. ANOVA and pairwise Student’s *t*-test were performed. Enriched groups: Non-Perio, Perio and tie, equally contributed. Cliff’s deltas were calculated relative to Non-Perio. **D** Ratios (log_2_) of the abundance (counts) of *Porphyromonas* to *Rothia* in SBP and *Coprococcus* to *Lachnoclostridium*. Wilcoxon rank sum test. ROC of two ratio pairs for discriminating between Perio and Non-Perio

To examine the contribution of each taxon toward periodontitis, we further employed the supervised sPLS-DA on microbiome of SBP, MSA and MST (Table S11). The prominent subgingival periodontopathogen genera enriched toward Perio (AUROC_Perio−vs.−Non−Perio_ > 0.70, Cliff’s delta_Perio−vs.−Non−Perio_ < − 0.4), while oral commensal representatives linked to Non-Perio (Fig. 3A, C). However, salivary genera showed different profiles with smaller loadings (|loading| < 0.2) of importance (Fig. S8). Outstandingly, fecal *Coprococcus* (AUROC_Perio−vs.−Non−Perio_ = 0.67, Cliff’s delta_Perio−vs.−Non−Perio_ = − 0.34) favored periodontitis, whereas *Lachnoclostridium* (AUROC_Non−Perio−vs.−Perio_ = 0.69, Cliff’s delta_Perio−vs.−Non−Perio_ = 0.39) from the same family Lachnospiraceae connected to Non-Perio (Fig. 3B), reiterating the intra-Lachnospiraceae divergency toward periodontitis. The sPLS-DA of microbiota distinctly differentiated between Perio and Non-Perio in all three types of samples (Fig. 3C). Additionally and comparably, the abundance ratios of subgingival *Porphyromonas* to *Rothia* (P/R ratio), as a signature of periodontal dysbiosis [22], and fecal *Coprococcus* to *Lachnoclostridium* (C/L ratio) were significantly (*P* < 0.01) higher in Perio group (AUROC_Perio−vs.−NonPerio_ > 0.7, Fig. 3D). The random forest identification with Boruta algorithm further corroborated the results of DESeq2, ANCOM-BC2, and sPLS-DA results (Fig. S9). These observations imply that periodontitis could alter both oral and gut microbiomes of first-trimester pregnant women.

### Microbiome network analysis revealed oral-gut links at first trimester

**Fig. 4:**
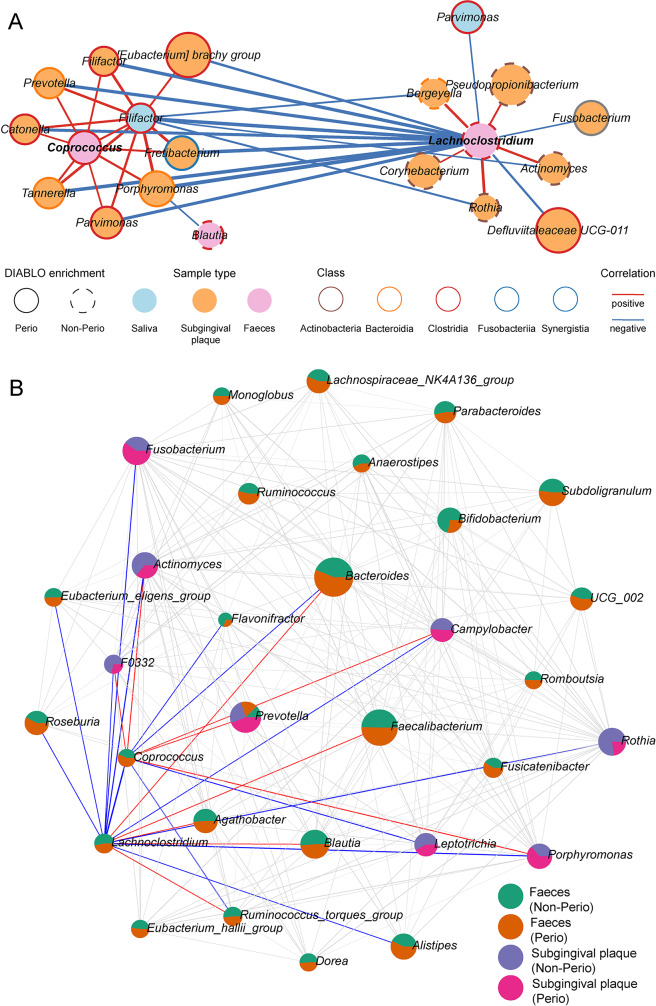
Inter-sample network of microbiota genera in the pregnant women with Perio (*n* = 31) and Non-Perio (*n* = 23). **A** Inter-sample network integrated by DIABLO algorithm for periodontitis-discriminative genera among subgingival plaques, saliva and feces (correlation |*r*| > 0.3). Nodes of the ‘tie’ genera were not shown. The line width represented |*r*|. **B** Inter- and intra-sample SECOM (Pearson1) correlation network (absolute Pearson coefficient |*r*| > 0.3 and *P* < 0.05). The correlations for fecal genera *Coprococcus* and *Lachnoclostridium* were highlighted with red lines as positive correlation and blue lines as negative one while grey lines for other correlations. The areas of pies represent the medians of relative abundance of genera. The detailed SECOM correlation is provided in Table S12

Considering periodontitis-related alteration of the gut microbiota, we further explored the potential microbiome network of oral-gut axis. DIABLO integration genera from the three sites revealed a periodontitis-discriminative correlation (*r* > 0.3) network with two apparent clusters for Perio and Non-Perio (Fig. 4A). Perio-enriched fecal genus *Coprococcus* and Non-Perio-enriched *Lachnoclostridium* associated distinctly with subgingival pathogens and commensals, respectively.

To unveil intra-microbiota relationship, SECOM (Pearson1 mode) was employed to validate the intra- and inter-connection of subgingival and fecal microbiota, which differed distinctly within the inter-connection network (Fig. 4B, Table S12), except for *Prevotella*, an health- and disease-related diverse and ubiquitous genus in various human niches [23]. The highlighted *Coprococcus* positively linked to periodontopathogenic *Porphyromonas* (SECOM (Pearson1) = 0.760) but not to *Fusobacterium*, whereas inversely correlated to subgingival *Leptotrichia* as well as fecal *Bacteroides* and *Lachnoclostridium* (SECOM (Pearson1) = − 0.33 and − 0.415). Contrarily, *Lachnoclostridium* was positively connected to *Faecalibacterium* and negatively linked with subgingival genera. Taken together, there is the potential periodontitis-promoted oral-gut linkage of microbiota in early pregnancy.

### Periodontitis altered microbiome functions in subgingival and fecal milieux

Functional prediction using PICRUSt2 integrated with ANCOM-BC2 (Table S13) showed top Perio-enriched enzymes (Enzyme Classification, EC) and KEGG orthologs (KO) in SBP, including lipopolysaccharide biosynthesis (wecG and wbpB) and *P. gingivalis* virulence factor gingipain R (K08589) (*P* < 0.01). KEGG enrichment on KOs (Fig. S10, Table S14) revealed multiple periodontitis-related pathways (e.g., map00860: Porphyrin metabolism; map00650: Butanoate metabolism; map00540: Lipopolysaccharide biosynthesis) in SBP. Consistently, Perio-enriched MetaCyc ontology predictions in SBP consisted of two pathways related to the fermentation of short-chain fatty acids (SCFAs, e.g., butanoate), namely P163-PWY (L-lysine fermentation to acetate and butanoate) and PWY-5677 (succinate fermentation to butanoate) (*P* < 0.005). In feces, both ECs and KOs enriched dominantly in Non-Perio, and KEGG enriched metabolic pathways (e.g., map00121: Secondary bile acid biosynthesis, *bai* operon; map00650; map00640: Propanoate metabolism, *pdu* operon), which were attenuated in Perio. For MetaCyc, energy-related metabolic pathways, butanoate-related P163-PWY and PWY-5677 in feces were downregulated in Perio. In different milieux, periodontopathogen-producing SCFAs could disrupt periodontal tissues [24], while fecal microbiota-derived ones may benefit gut health and immunity [25], consistent with our findings on increased microbial butyrate/SCFA synthesis in subgingival plaques while reduced in feces of Perio subjects.

Moreover, there were significant correlations between genera and PICRUSt2-predicted functions and pathways (Table S15) in SBP and MST. In SBP, the SCFA-producing pathways correlated to *Porphyromonas* (ρ > 0.5, FDR < 0.001), and *Actinomyces* and *Rothia* (ρ ≤ −0.3, FDR ≤ 0.05). Periodontitis-enriched fecal *Coprococcus* negatively linked to glucarate/galactarate-degrading and SCFA-related pathways P163-PWY and PWY-5677 (− 0.6 < ρ < −0.4, FDR < 0.01). Furthermore, *Akkermansia* exhibited moderate correlations (ρ > 0.3, FDR < 0.05) to pathways including SCFA-related ones (Fig. S11). KOs in feces for both *bai* and *pdu* operons were significantly negatively correlated with *Coprococcus*. Therefore, we speculated that periodontitis may perturb microbiome functions via subgingival periodontopathogens and fecal bacteria.

### Periodontitis contributed to the variation of metabolic features within feces

**Fig. 5:**
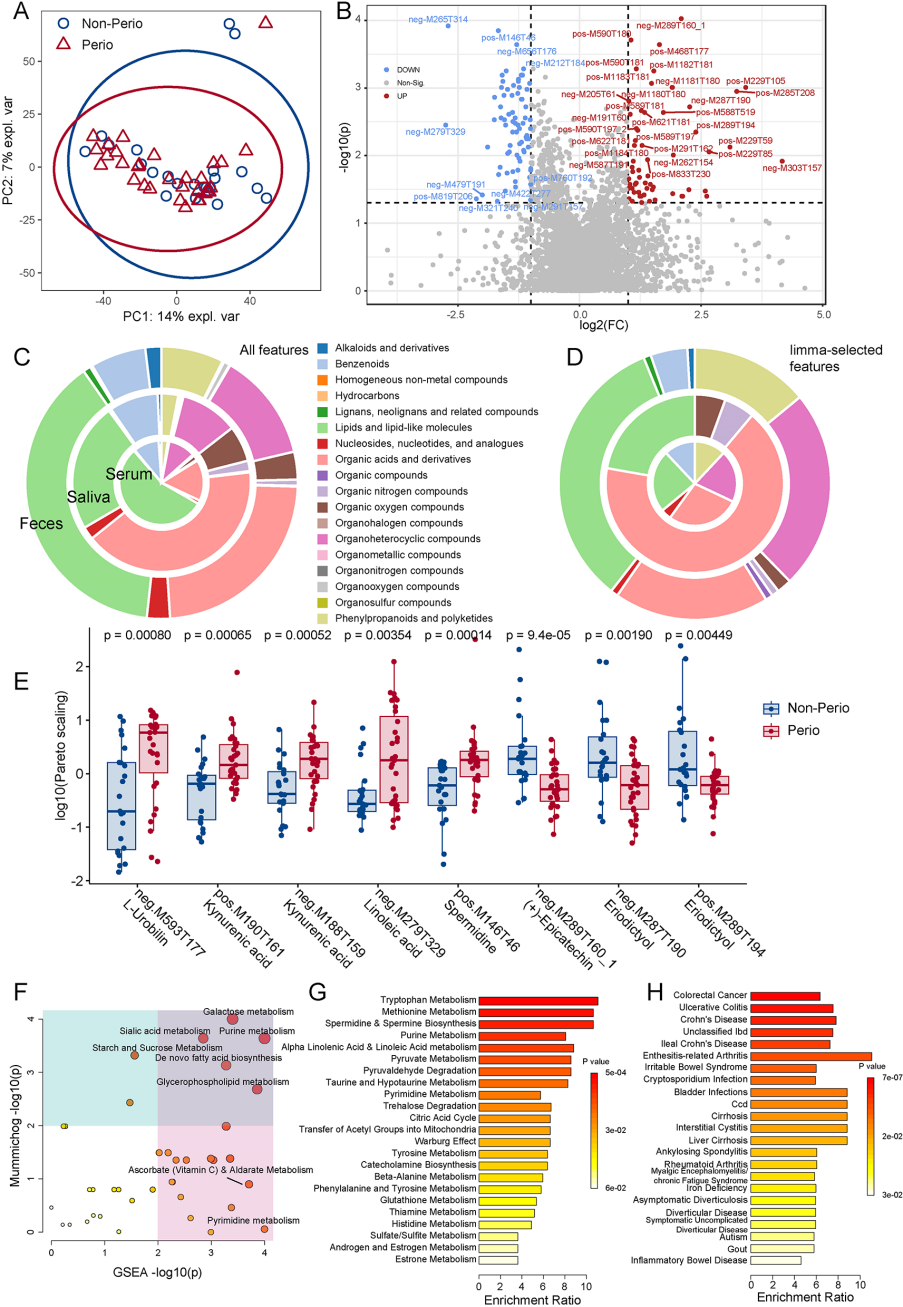
Comparison of fecal metabolites between the pregnant women with Perio (*n* = 31) and Non-Perio (*n* = 23). **A** Principal component analysis score plot. 95% confidence ellipses were shown. **B** Volcano plot revealed the inter-group difference in metabolic features (*n* = 137). Features with |log_2_FC| > 1 and *P* < 0.05 (Wilcoxon signed-rank test) were colored and labelled. Chemical classification of metabolic features in samples (**C**) and limma-identified and BMI-adjusted differentially changed (*P* < 0.05) metabolic features (**D**, *n* = 547) of feces, saliva and serum based on ClassyFire classification. **E** Boxplot of selected differentially changed metabolic features in feces. Wilcoxon rank sum test. **F** Functional enrichment analysis on limma-identified and BMI-adjusted differentially changed (*P* < 0.05) metabolic features in feces (*n* = 547) with integration of mummichog and GSEA algorithms. Quantitative metabolite set enrichment analysis (qMSEA) on MS2- and HMDB-annotated fecal metabolic features (limma-identified BMI-adjusted differentially changed, *P* < 0.05) for pathways (**G**) and disease signatures of HMDB (**H**). The detailed results are presented in Tables S19–S23

Metabolic features (positive and negative modes) were detected in saliva, serum, and feces, respectively (Table S16); with further annotation of secondary ion mass spectra (MS2) (Table S17), filtering and log_10_-transformed Pareto scaling (Table S18). PERMANOVA (Euclidean distances) revealed significant alteration in fecal metabolites (*P* = 0.036, Fig. 5A, Table S19) rather than salivary and serum ones. Volcano plot showed 137 changed fecal features (*P* < 0.05, Wilcoxon signed-rank test; and fold change FC > 2, Fig. 5B), while fewer features were detected in saliva and serum. ‘Lipids & lipid-like molecules’ and ‘Organic acids & derivatives’ were the major superclasses for all features in feces, saliva and serum (Fig. 5C).

We integrated *limma*, generalized linear model (GLM) and sPLS-DA for selecting periodontitis-discriminative metabolic features. The *limma* approach showed that totally 448 fecal features were differentially (*P* < 0.05) altered (Fig. S12, Table S20), mostly belonging to the ‘Lipids & lipid-like molecules’, ‘Organoheterocyclic compounds’ and ‘Organic acids & derivatives’ (Fig. 5D). In serum, the detected sex hormone features were insignificantly different between Non-Perio and Perio (Fig. S13), thus minimizing the influence of pregnancy and sex hormones [9] and reflecting similar gestational weeks of sample collections (Table S4). Notably, three MS2-annotated metabolites were highly significant (FDR < 0.05) including Perio-increased L-urobilin (HMDB0004159, log_2_FC: 0.94) and kynurenic acid (KYNA, HMDB0000715, log_2_FC: 0.64) as well as Perio-decreased norbuprenorphine glucuronide (NBUP-G). Nine more features (FDR < 0.1) existed, such as increased linoleic acid and spermidine, and decreased flavonoids as (+)-epicatechin and eriodictyol (Fig. 5E). In serum, three indole derivatives were enriched in Perio, suggesting the alteration of tryptophan metabolism pathway [26]. Notably, MS1-annotated fecal bilirubin, D-urobilin, D-urobilinogen and I-urobilinogen (mesobilirubinogen) and serum biliverdin, bilirubin and mesobilirubinogen decreased in Perio group (*P* < 0.05).

Functional enrichment analysis revealed significant fecal metabolic pathways like galactose, sialic acid, purine and glycerophospholipid (Fig. 5F, Table S21), with poor enrichment in saliva and serum. Further quantitative metabolite set enrichment analysis (qMSEA) for pathways and disease signatures on annotated fecal metabolic features revealed multiple metabolic pathways amino acids and derivatives (Fig. 5G and Table S22), and yet gastrointestinal diseases (Fig. 5H, Table S23).

Next, GLM found 387 differential metabolic features in feces. Of them, 92 were MS2-annotated, including 56 MS2-annotated metabolites associated with Perio (positive coefficients). Comparingly, much fewer associated metabolites were found in saliva and serum (Table S24). Among the sPLS-DA selected features (Fig. S14, Table S25), the most notable fecal features for Perio were those of KYNA, spermidine and L-urobilin (AUROC_Perio_vs_Non−Perio_ > 0.7, Cliff’s delta_Perio−vs.−Non−Perio_ < − 0.53), while (+)-epicatechin and NBUP-G contributed mostly to Non-Perio. Mummichog and gene set enrichment analysis (GSEA) integrative enrichment revealed significant metabolic pathways (Fig. S15, Table S26). Collectively, 296 (70 MS2-annotated) significant features were enriched via *limma*, GLM and sPLS-DA (Fig. S16, Table S27). Fecal L-urobilin could distinguish IBD from non-IBD [27]; while KYNA has Janus-faced effects on healthy and diseased conditions, including IBD, neurological disorders and pregnancy [28, 29]. Collectively, our findings reflected the marked alterations in fecal metabolites of the first-trimester pregnant women with periodontitis. Additionally, the DIABLO-established network integrating the metabolomes of saliva, serum, and feces (*r* > 0.5, Fig. S17) revealed the pivotal metabolic features, i.e., L-urobilin (feces), hypoxanthine and pyroglutamic acid (serum) with the most Perio-associated links (*r* > 0.6), thereby suggesting a periodontitis-associated oral-gut link among metabolites.

### Integrative analysis revealed the interconnection of fecal microbiota and metabolites as well as clinical determinants

Spearman’s correlation demonstrated notable association of key periodontal parameters with relative abundance of periodontopathogens and commensals in SBP (Fig. S18A, Table S28), and positive intercorrelation of *Tannerella* with BMI. DIABLO-integrated network revealed the Perio cluster of ‘triad’ (*Porphyromonas*, *Fretibacterium* and *Tannerella*) with triglyceride and CRP, and the serum albumin-centered Non-Perio cluster (Fig. S18B). In saliva, *Filifactor* correlated with periodontal parameters and BMI. We further showed the positive correlation between fecal *Coprococcus* (centered log-ratio, clr) and periodontal parameters (FDR < 0.05, Fig. S19, Table S29).

**Fig. 6:**
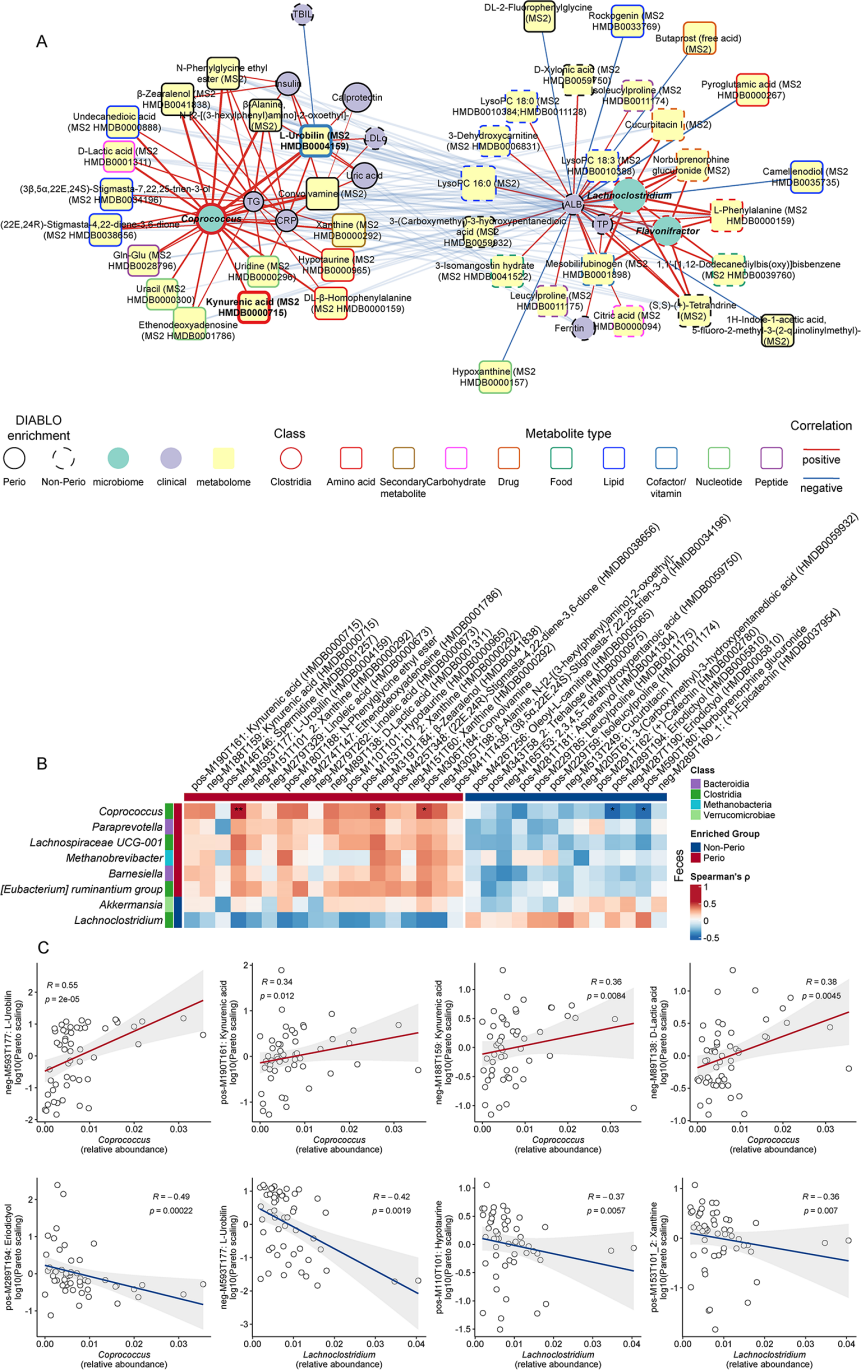
Integration of fecal microbiota, metabolites and systemic clinical determinants in the pregnant women with Perio (*n* = 31) and Non-Perio (*n* = 23). **A** DIABLO-integrated correlation network of fecal microbiome, metabolome and clinical numeric determinants. Nodes of ‘tie’ of sPLS-DA were not shown. The line width represented |*r*|. The correlation coefficient threshold with clinical determinants was |r|>0.3, while the one between microbiome and metabolome was |r|>0.4. Two clusters were observed, and all the inter-cluster links were shown with translucency for clarity. When no HMDB ID was presented, the feature was annotated by an in-house library only. CRP, C-reactive protein; TG, triglyceride; LDL-c, low-density lipoprotein cholesterol; TBIL, total bilirubin; TP, total protein; ALB, serum albumin. **B** Pairwise Spearman’s correlation analysis between DESeq2-enriched genera (relative abundance) in feces and Venn-selected MS2-annotated metabolic features with FDR (Benjamini-Hochberg) adjustment for *P* values. Enriched groups for microbiome were from DESeq2 results, while enriched groups for metabolome were from the sPLS-DA model. Correlation matrices were presented with asterisks representing the significance of FDR-adjusted *P* values: * FDR < 0.05, ** FDR < 0.01. The exact adjusted *P* values (FDR) are provided in Table S30. **c** Scatter plots of Spearman’s correlation between selected fecal genera and metabolic features

Furthermore, DIABLO (Fig. S20) discovered sub-network clusters that could reflect Non-Perio and Perio containing key microbial, metabolic, and clinical indicators (Fig. 6A). Essentially, fecal *Coprococcus*, L-urobilin and serum triglyceride could be proposed as an attributive ‘triad’ for Perio with most links; while for Non-Perio cluster, *Lachnoclostridium* and serum albumin would be the critical players. Further Spearman’s correlation on Perio-enriched microbiome and metabolome (Fig. 6B, C, Table S30) demonstrated strongly positive correlation of *Coprococcus* with L-urobilin (FDR < 0.01) and negative correlation with eriodictyol and NBUP-G (FDR < 0.05). Using clr-transformed abundance, *Lachnoclostridium* showed negatively related metabolic features with FDR significance, e.g., L-urobilin, hypotaurine and xanthine (Fig. S21, Table S31). Taurine and hypotaurine metabolism has been considered to be beneficial for health [30], though its enhancement also connects to IBD, *Clostridioides difficile* infection and even gestational DM (GDM) [27, 31, 32].

## Discussion

Periodontitis closely links with numerous systemic diseases [2], with great socio-economic burdens. Notably, although studies have revealed the connection of periodontitis with APOs, the underlying mechanism and clinical causality remain unclear [3] due to limited multi-omics and multi-body-site studies available. The present study has investigated the impacts of maternal periodontitis in first-trimester pregnant women on the microbiome and metabolome profiles, without substantial regional bias included (Supplementary Text). There is no significant difference in gestational age and serum metabolic features of sex hormones between Non-Perio and Perio subjects, which further minimizes bias brought by pregnancy and sex hormones on both microbiome and metabolome [9]. Additionally, there is no significant alteration of periodontal status between the first and third trimesters [8], suggesting limited influence of pregnancy per se on the periodontal conditions of subjects. Overall, we have found that periodontitis extensively affects subgingival microbiota and fecal metabolites in early pregnant women, echoing the oral-gut interaction on maternal and neonatal health [9].

It is known that periodontopathogens account for most elevated subgingival taxa of pregnant women with periodontitis [2]. Unlike previous studies on common subjects [33], both salivary microbiota and metabolites of pregnant women during their first trimester are barely perturbed by periodontitis, possibly due to the masking effects of natural increase of periodontopathogens in saliva during pregnancy [5]. Surprisingly, we identify that *Coprococcus* genus from Lachnospiraceae could be a key maternal fecal genus responding to periodontal diseases in the first trimester. Lachnospiraceae members (*Coprococcus*, Lachnospiraceae UCG-001, *Lachnoclostridium*, *Sellimonas*, and *Hungatella*) demonstrate most intra-family diverged response to periodontal status, which has also been observed in the study on depression [34]. Possibly due to the moderate periodontitis in Perio subjects, we have not found ectopic colonization of periodontopathogens like *P. gingivalis* in feces as an important factor in aggravating colitis [35]. The decreased function of butyrate synthesis in negative association with periodontitis-enriched *Coprococcus* from Perio group indicates that periodontitis could reshape intestinal milieu to unhealthy state by disrupting butyrogenesis, as fecal microbiome-derived SCFAs like butyrate are critical to gut health and immunity [25] and yet complications of pregnancy like GDM and preeclampsia [9]. Indeed, clinical study shows that *Coprococcus* increases in periodontitis subjects [4]. Moreover, human fecal *C. comes* positively links to monocyte-derived inflammatory (cytokines interleukins 1β and 6) and acute phase protein but negatively to IL-22 [36] though Lachnospiraceae family is usually considered as commensals and beneficial to gut and systemic health, due to its fiber-degradation and butyrogenesis [37].

Most Lachnospiraceae and Ruminococcaceae deplete in feces of periodontitis subjects in early pregnancy, which is consistent with other studies on common diseases like IBD [27], while *Coprococcus* abundance increases, possibly in line with the increased inflammation in pregnant women with periodontitis [3]. The positive correlation of *Coprococcus* with periodontopathogens and periodontal parameters further distinguishes itself with other Lachnospiraceae members. Although there were only three patients with GDM and no hypertension case in our study, *Coprococcus* positively links to Perio-associated triglyceride, CRP, and fasting blood glucose, echoing that *Coprococcus* member links to serum metabolic and corpulence traits as well as clinical comorbidities [38, 39], possibly due to fructose-induced obesity [40]. Actually, in this study, Perio subjects had significantly higher BMI at the third trimester and delivery. *Coprococcus* is positively correlated with increased gestational weight gain and risk of developing GDM [41], and over-represents in pregnant women with Crohn’s disease (CD) [42]. Moreover, most SCFA-producing bacteria including *Coprococcus* deplete in various diseases such as IBD [27], GDM [43] and preeclampsia [44], possibly reflecting the ‘butyrate paradox’ [45] and attributing to lifestyle behaviors like westernized dietary [23]. During pregnancy, *Coprococcus* abundance increases from early to late pregnancy and the onset of GDM may hinder this observation [46], possibly explaining the discrepancy, as our subjects are at the first trimester and it would be interesting to examine those at the third trimester. Nevertheless, we have found the positive link of fecal *Coprococcus* with most periodontopathogens that produce SCFAs detrimental to periodontal tissues [24]; yet the ectopic *Porphyromonas* members *P. gingivalis* and *P. asaccharolytica* in gut may induce cellular senescence via secreting butyrate, thereby promoting colorectal cancer [45].

Periodontitis impacts fecal metabolites more broadly with elevated periodontitis-associated features in feces (e.g., L-urobilin, kynurenic acid, linoleic acid, spermidine, D-lactic acid, hypotaurine and xanthine). These metabolic features account for metabolism pathways concerning heme, tryptophan, arginine and cysteine, and most of which positively correlate with *Coprococcus* and negatively with *Lachnoclostridium*. Urobilin is one of the end-products of gut bacteria-mediated bilirubin (a heme catabolic product and antioxidant [47]) deconjugation, being a part of hepatic function [47]. While lacking significant alteration in serum total bilirubin (TBIL), it is non-Perio-associated and negatively correlated with fecal L-urobilin by sPLS-DA in connection to *Coprococcus*. Actually, TBIL decreases in periodontitis patients [48], and it is negatively associated with diabetes and its complications [49], suggesting an existence of oral-hepatic axis and bilirubin as a causal risk of periodontitis [50]. Regarding our *limma* and GLM findings, fecal L-urobilin (stercobilin) and L-urobilinogen (stercobilinogen) increase in Perio group while fecal bilirubin, D-urobilin, D-urobilinogen and I-urobilinogen (mesobilirubinogen) and serum biliverdin, bilirubin and mesobilirubinogen decrease, revealing a heterogeneously divergent bilirubin-derivative urobilinoid preference. Noticed with similar divergence, fecal L-urobilin depletes in IBD [27, 31], D-urobilin and L-urobilinogen elevate in spontaneous preterm birth patients and link to saturate fat intake [51], and D-urobilinogen increases in miscarriage patients and connects to pro-inflammatory cytokines and interferons [52]. Additionally, fecal L-urobilinogen is associated with obesity, and the levels of multiple urobilinoids rise in murine obesity models [53]. In heme degradation pathway, the released heme during eryptosis is sequentially converted to bilirubin and solubilized via conjugation to glucuronic acid in liver before secreted into bile [47]. In gut, conjugated bilirubin is mainly deconjugated by gut microbial β-glucuronidases (GUS) [54]. GUS can suppress proteolytic activity (PA) in gut, which links to the intestinal health [54]. Periodontitis may reduce GUS activity, as our findings on the decrease of fecal NBUP-G in Perio group. A recent study has identified a missing piece of the reduction of bilirubin to urobilinogen, the gut-microbiota-derived bilirubin reductase BilR [55]. Indeed, BilR is strain-specific and mainly found in the class Clostridia of the phylum Firmicutes. Perio-depleted genera found in this study, including *Lachnoclostridium*, *Catenibacterium*, *Hungatella*, and *Holdemania*, may possess BilR, which to some extent explains the reduced metabolic features for D-urobilinogen and its oxidized product D-urobilin. However, the gut microbial enzyme for sequential reduction of D-urobilinogen to L-urobilinogen remains unknown, which may be the key for solving discrepancy between L-urobilin and D-urobilin. In line with that bilirubin reversely affects bile acids (BAs) [47], the levels of total BA and forms of BAs increases in periodontitis subjects [56]. Additionally, attenuated fecal *bai* operon activity in Perio, which is negatively associated with *Coprococcus*, suggests weakened detoxification of secondary BAs that is harmful to gestation [57].

KYNA is a widely present tryptophan catabolic by-product of kynurenine pathway (KP), GPR35 agonist and aryl hydrocarbon receptor (AhR) ligand [28], and it is also produced by gut microbial enzymes [58]. Increased KYNA is a Janus-faced indicator of health or disease [28, 29]. Elevated blood level of KYNA and its correlation with proteinuria are identified in the first trimester of women with preeclampsia [29], possibly due to kynurenine-promoted NK cytotoxicity [59]. The gut microbiota-induced KYNA could promote encephalitis [58]. Besides KYNA, we also find increased KP by-product picolinic acid, while other tryptophan metabolites are not perturbed. KP exhibits pleiotropic effects on immunosuppression, detrimental tumorigenesis and neuroprotection or neurotoxicity, via indoleamine 2,3-dioxygenase and AhR activities [28]. Oral administration of *P. gingivalis* in mice alters tryptophan metabolism, increasing serum kynurenine as a direct precursor of KYNA while reducing fecal and serum AhR activities [26], in consistence with blood kynurenine and tryptophan metabolism as causal risks for periodontitis [50].

As taurine is a semi-essential amino acid and ubiquitous in eukaryotes, its dietary intake has extensive benefits to health and healthy aging [30]. Decreased levels of taurine and its metabolites in blood are associated with several metabolic disorders, such as obesity, hypertension and T2DM; while their elevated levels in feces relate to IBD and *C. difficile* infection [27, 31]. Notably, taurine is overabundant in maternal blood of GDM patients and meconium of their neonates [32]. Our study shows that *Lachnoclostridium* is negatively correlated with increased fecal hypotaurine in early pregnant women with periodontitis along with a purine metabolite xanthine, which is suggested as a causal risk of loose teeth [50] and over-represented in maternal immune activation casting adverse effects on offsprings [60]. Recently, we also discovered that maternal periodontitis could perturb the leukocyte profile among umbilical cord blood [61] and link to the neonatal adverse outcome − small-for-gestational-age (SGA) newborns [62].

Some limitations of the current study may need to be addressed. Firstly, a group of women with successfully treated periodontitis prior to conception could have been included in the study for warranting the positive rationale and importance of oral/periodontal management during the whole peripregnancy. Secondly, due to the limited number of subjects, there are only a few cases of APOs observed. Thus, the links between periodontitis-associated features with APOs could not be convincingly established. Thirdly, only Han residents in the metropolitan Shenzhen were recruited in the study, thereby restricting the generalization of our current findings. Finally, 16S rRNA amplicon sequencing and untargeted metabolomics may limit the resolution of feature selection and fineness of data integration. As ethnic and regional diversity accounts for disparities in microbiome and metabolome as well as APO-associated alterations of vaginal niche [6, 11], further validation and investigations with large sample-sized, multi-center and longitudinal cohorts and inclusion of periodontal intervention are required. These works would substantiate the current evidence of the possible causality of distal impact of periodontitis, and elucidate the translational implications of preconceptional and peripregnancy periodontal healthcare for enhancing general health and maternal wellbeing.

## Conclusions

In the present study, we have investigated the clinical traits, microbiome and metabolome profiles in pregnant women with or without periodontitis at their first trimester as the key period of early life [7]. Maternal periodontitis increases the fasting blood glucose and decreases the serum albumin levels; yet dramatically alters subgingival microbiota and surprisingly diverges multiple key taxa of Lachnospiraceae family in gut microbiome like elevated *Coprococcus* and decreased *Lachnoclostridium* abundance. Unexpectedly, periodontitis perturbs more fecal metabolites than salivary and serum ones, mainly involving the metabolism pathways of heme, tryptophan, arginine and cysteine. Importantly, the pivotal Perio-triad of *Coprococcus*, triglyceride and L-urobilin during early pregnancy reflects the potential extra-oral impacts of periodontitis through the oral-gut axis. This study highlights the profound importance of proactively promoting integration of oral/periodontal and maternal healthcare via the great teamwork of dental-medical professionals. It also emphasizes the great need of further incorporation of oral healthcare into obstetric and gynecological management scheme in national and global maternal healthcare guidelines and policies [63].

### Electronic supplementary material

Below is the link to the electronic supplementary material.

Supplementary Material 1

Supplementary Material 2

Supplementary Material 3

Supplementary Material 4

Supplementary Material 5

Supplementary Material 6

Supplementary Material 7

Supplementary Material 8

Supplementary Material 9

Supplementary Material 10

Supplementary Material 11

Supplementary Material 12

Supplementary Material 13

Supplementary Material 14

Supplementary Material 15

Supplementary Material 16

Supplementary Material 17

Supplementary Material 18

Supplementary Material 19

Supplementary Material 20

## Data Availability

The raw sequencing and metabolomic datasets reported in this paper have been deposited in the Genome Sequence Archive (GSA) and Open Archive for Miscellaneous Data (OMIX) in National Genomics Data Center, China National Center for Bioinformation/Beijing Institute of Genomics, Chinese Academy of Sciences (BioProject: PRJCA021796, GSA Accession Nos.: CRA014011 and CRA014012, OMIX Accession No.: OMIX006154) that are publicly accessible at https://ngdc.cncb.ac.cn/. Other data in the figures are provided in the Supplementary Tables. The R codes used for analysis and figure are available at: https://github.com/tfccheng/microbiome-metabolome-periodontitis-trimester1.
