# Supplementary material for: Integrative microbiome and metabolome profiles reveal the impacts of periodontitis via oral-gut axis in first-trimester pregnant women
Source: J Transl Med. 2024 Sep 3;22:819. doi: 10.1186/s12967-024-05579-9 (PMC11370083; doi:10.1186/s12967-024-05579-9)
Supplement: Supplementary file 1 — Supplementary Material 1 [file 12967_2024_5579_MOESM1_ESM.pdf]

Supplementary Information

Integrative microbiome and metabolome profiles reveal the impacts of periodontitis via oral-gut axis in first-trimester pregnant women

Tianfan Cheng,[1]* Ping Wen[2], RongYu[2], Feng Zhang[3], Huijun Li[3], Xiaoyi Xu[2,4], Dan Zhao,[1,5] Fang Liu[6], Weilan Su[6], Zheng Zheng[6], Hong Yang[6], Jilong Yao[6], Lijian Jin[1]*

[1]Division of Periodontology & Implant Dentistry, Faculty of Dentistry, The University of Hong Kong, Hong Kong, China;

[2]Institute of Maternal and Child Medicine, Shenzhen Maternity & Child Healthcare Hospital, Shenzhen, China;

[3]Division of Stomatology, Shenzhen Maternity & Child Healthcare Hospital, Shenzhen, China;

[4]Center for Disease Control and Prevention, Shenzhen, China;

[5]Department of Implant Dentistry, Beijing Stomatological Hospital, Capital Medical University, Beijing, China;

[6]Division of Obstetrics & Gynecology, Shenzhen Maternity & Child Healthcare Hospital, Shenzhen, China.

*Correspondence: chengtfc@hku.hk (TC); ljjin@hku.hk (LJ)

Outline

Supplementary figures

Figure S1. Principal component analysis of systemic clinical parameters between Perio (n = 31) and Non-Perio (n = 23) groups.

Figure S2. MixMC analysis shows distinct microbiota from subgingival plaque, saliva, and feces.

Figure S3. Relative abundance of phyla among the microbiota from subgingival plaque, saliva, and feces.

Figure S4. Relative abundance of genera among the microbiota from subgingival plaque, saliva, and feces.

Figure S5. Relative abundance of families among the microbiota from subgingival plaque, saliva, and feces.

Figure S6. Beta diversity of microbiota from subgingival plaque, saliva, and feces.

Figure S7. DESeq2-selected significantly differentially abundant genera in subgingival plaques, saliva, and feces between Perio (n = 31) and Non-Perio (n = 23) groups.

Figure S8. sPLS-DA-selected important genera in saliva between Perio (n = 31) and Non-Perio (n = 23) groups.

Figure S9. Boruta-selected important genera for classification between Perio (n = 31) and Non-Perio (n = 23) groups.

Figure S10. KEGG Ortholog enrichment of significant functional KOs predicted by PICRUSt2.

Figure S11. Fecal genera correlation with PICRUSt2-predicted pathways.

Figure S12. limma analysis of fecal metabolome.

Figure S13. Serum sex hormones.

Figure S14. sPLS-DA-selected important metabolic features in feces of pregnant women.

Figure S15. Functional enrichment analysis on sPLS-DA-selected metabolic features in feces (n = 300) with integration of mummichog and GSEA algorithms.

Figure S16. Venn plots of periodontitis-enriched metabolic features from limma, sPLS-DA, and GLM methods.

Figure S17. DIABLO integration of metabolomes of saliva, serum, and feces.

Figure S18. Relationship between microbiomic genera and clinical numeric determinants.

Figure S19. Relationship between microbiomic genera and clinical numeric determinants.

Figure S20. Integration of fecal microbiota, metabolites, and systemic clinical determinants between Perio (n = 31) and Non-Perio (n = 23) groups using DIABLO.

Figure S21. Spearman's correlation between microbiomic genera and metabolic features in feces.

Supplementary tables

Table S1. The demographic, periodontal and clinical datasets of the 54 subjects

Table S2. Demographic characteristics of the 54 subjects via the questionnaires

Table S3. Other demographic characteristics of the 54 subjects via the questionnaires

Table S4. Summary of clinical parameters

Table S5 XCMS parameters

Table S6 metaX parameters

Table S7. Summary of relative abundance of phyla, families and genera among subgingival plaques, saliva, and feces in both Non-Perio and Perio groups

Table S8. Summary of PERMANOVA (Bray-Curtis distances) of microbiome data

Table S9. Summary of taxa (families, genera, species and ASVs) identified as differentially abundant between Perio and Non-Perio groups using DESeq2 with or without adjustment with BMI

Table S10. Summary of taxa (families, genera and species) identified as differentially abundant between Perio and Non-Perio groups using ANCOM-BC2 with or without adjustment with BMI

Table S11. Summary of taxa (genera, species and ASVs) identified as differential contributors between Perio and Non-Perio groups using sPLS-DA

Table S12. Intra- and inter-correlation of genera between subgingival plaque and feces samples using SECOM (Pearson1 mode)

Table S13. Summary of PICRUSt2-predicted functions identified as differentially abundant between Perio and Non-Perio groups using ANCOM-BC2

Table S14. KEGG enrichment of ANCOM-BC2-selected PICRUSt2-predicted KO terms

Table S15. Summary of Spearman's correlation between DESeq2-selected genera (clr) and ANCOM-BC2-selected PICRUSt2-predicted functions

Table S16 Summary of detected ion features of metabolites in saliva, serum and feces

Table S17. Details of MS2-annotated metabolic features (adducts)

Table S18. Filtered features with log10-transformed Pareto scaling

Table S19. Summary of PERMANOVA (Euclidean distances) of metabolome data

Table S20. limma analysis of metabolic features

Table S21. The enriched pathways predicted jointly from mummichog and GSEA methods of limma-selected fecal features

Table S22. Quantitative metabolite set enrichment analysis of limma-selected fecal features using SMPDB database

Table S23. Quantitative metabolite set enrichment analysis of limma-selected fecal features using database of Disease Signatures in Feces

Table S24. GLM analysis of metabolic features

Table S25. sPLS-DA analysis of metabolic features

Table S26. The enriched pathways predicted jointly from mummichog and GSEA methods of sPLS-DA-selected fecal features

Table S27. Venn plot analysis of the fecal features selected by DESeq2, GLM and sPLS-DA

Table S28. FDR-adjusted P values of Spearman's correlation between relative abundance of genera (subgingival plaque and saliva) and clinical parameters

Table S29. FDR-adjusted P values of Spearman's correlation between clr of genera (feces) and clinical parameters

Table S30. FDR-adjusted P values of Spearman's correlation between relative abundance of genera (feces) and Venn-selected fecal metabolic features

Table S31. FDR-adjusted P values of Spearman's correlation between clr of genera (feces) and Venn-selected fecal metabolic features

Supplementary materials and methods

Clinical examination

Full-mouth periodontal examination was performed, and the details were presented in our recently published work [1]. The medical and biochemical datasets were retrieved from the internal records of SMCHH [2], and these parameters in all participants were included in the downstream analysis.

The subjects were grouped on the basis of diagnosis of periodontitis according to the current classification of periodontal diseases and conditions [3], i.e., 31 Periodontitis subjects (Perio group) and 23 Periodontal Health/Gingivitis subjects (Non-periodontitis/Non-Perio group).

Collection of samples

Unstimulated saliva, subgingival plaque, serum and stool samples were collected within one week at a convenient time for all subjects. Samples were stored at −80°C until further assessment.

1. **Sampling of unstimulated saliva and subgingival plaques**: All subjects were asked not to perform toothbrushing for 16 hours and eat or drink anything except water for 1 hour, prior to sampling. Subjects rinsed their mouth with sterile water, and then 5-min accumulated saliva was collected using a sterile 50 mL tube and immediately transferred into 2mL sterile tubes. Afterwards, subgingival plaques were collected at the 6 index teeth according to the Community Periodontal Index (CPI) system. The sampling sites were isolated by cotton rolls and carefully air-dried, and the supragingival dental plaque was removed by a sterile curette. A sterile absorbent paper point (Beijing Dayading, China) was gently inserted subgingivally, and kept for 15 seconds to collect subgingival plaques. The samples from all the 6 tooth sites were pooled in a 2mL sterile tube containing 750 μL of PowerBead Solution (Qiagen).
2. **Serum sampling**: A total of 5 mL sample of non-fasting peripheral blood was collected in Blood Collection Tubes (367406, BD Vacutainer), stood still for 30 min and then after centrifugation at 2,500 rpm (10 min) the supernatants were transferred into 2 mL sterile tubes.
3. **Fecal sampling**: Subjects were instructed to collect fresh fecal samples using a sterile feces tube, and the screw cap (Sarstedt) was provided by the research team. Samples were immediately transported to the laboratory within a cold pack.

Determination of fecal calprotectin by ELISA

Fecal levels of calprotectin were determined with the human calprotectin (S100A8/S100A9 Heterodimer) ELISA Kits (FineTest, China). Fecal samples were weighted and suspended by

adding 19-fold volume (v/w) of PBS to achieve 20-fold dilution. ELISA was performed following the instructions of the manufacturer.

Microbiome analysis

Microbiome community profiles and core microbiome were evaluated with *microbiome* v1.20.0. The observed counts of Minimally filtered dataset (MFD, singleton-removed) were used to calculate all Alpha indices (23 indices) utilizing the *alpha* function of microbiome v1.20.0 except Faith's phylogenetic diversity, which was calculated utilizing the *estimateDiversity* function of *mia* v1.6.0 package. The overall compositions of microbiota were assessed on relative abundance by principal coordinate analysis (PCoA) with *vegdist* function, and Permutational Multivariate Analysis of Variance (PERMANOVA) of Bray Curtis distances between Non-Perio and Perio was performed with *adonis2* function of *vegan* v2.6-4.

Differentially abundant taxa were firstly identified from the dataset of minima of relative abundance of 0.05% and prevalence of 5% (FD0.05), via the univariate approach of DESeq2 v1.38.1 [4] (Relative Log Expression normalization of counts, Wald test, Benjamini–Hochberg adjustment for multiple comparisons, FDR < 0.2) implemented *run_deseq2* function in microbiomeMarker 1.4.0 [5] and from observed counts in MFD using ANCOM-BC2 v2.0.1 [6] (FDR < 0.2). The nondefault parameters of DESeq2 were set for *run_deseq2* function in microbiomeMarker 1.4.0, as fitType = "parametric", sfType = "poscounts". The nondefault parameters of ANCOM-BC2 were set for *ancombc2* function, as prv_cut = 0, struc_zero = TRUE, neg_lb = TRUE.

Taxon features with large variation were selected using multivariate procedure MixMC framework for sample types and sPLS-DA for disease classification within each type of sample, from FD0.05 with robust centered log-ratio (rclr) transformation of relative abundance (FD0.05-rclr) using mixOmics v6.22.0 [7] with cross-validation setting of folds/repeats (10×10 and 20×50), respectively. Random forest [8] of FD0.05-rclr was trained using *caret* v6.0-93 and employing randomForest v4.7-1.1 for the repeatedly cross-validating optimization of splits per try (mtry) and numbers of trees (ntree), and the importance of variable was evaluated using the varImp function in *caret*, following which important features (overall importance > 40 or 50) were further selected by Boruta v8.0.0 [9] by the settings of the final RF models. To explore the inter- and intra-ecosystem relationships, both the algorithm DIABLO [10] developed for multi-omics integration and the SECOM (Pearson1) method [11] in MicrobiomeAnalyst 2.0 [12] were applied. For integration of DIABLO multi-body sites, FD0.05-rclr was used with

setting block link of the design matrix of 0.1 and folds/repeats (10×50) of cross-validation. The *network* function was used to generate correlation networks, exporting gml files for network visualization by Cytoscape [13] v3.10.0. While for SECOM network, the datasets of SBP and MST were filtered with a minimal 4 count with 20% prevalence and 10% IQR of low variance and scaled using total sum scaling (TSS) in MicrobiomeAnalyst with parameter setting as SECOM (Pearson1) algorithm, Genus level, Permutation (SparCC) of 100, P-value threshold of 0.05 and correlation threshold of 0.3.

Metabolite extraction, separation, and mass spectrum acquisition

Metabolomic analysis was performed using the ultra-performance liquid chromatography (UPLC) with tandem mass spectrometry (MS)/MS by Shenzhen Academy of Metrology and Quality Inspection, China. The metabolite extraction was followed to the modification of an established protocol [14]. Briefly, 100 ml or mg of collected sample were added with 1 mL precooled methanol (50%), vortexed (1 min) and then incubated (10 min) under room temperature. The extracted mixtures were further incubated at −20°C overnight for protein precipitation. The supernatants of extracts were collected by centrifugation at 4,000 g (20 min) and transferred to new 96-well plates for storage (−80°C) until the LC-MS analysis. Additionally, pooled quality check (QC) samples were made ready through combination of each extract (10 μL).

The metabolites in samples were separated and eluted in 0.1% formic acid (A) with the gradient of 0.1% formic acid in acetonitrile (B) at a flow rate of 0.4 ml/min, by the UPLC on an ACQUITY UPLC System (Waters) equipped with an ACQUITY UPLC T3 reversed phase column (100mm×2.1mm, 1.8μm, Waters) kept at 35°C. The elusion gradient was set as 0−0.5 min, 5% B; 0.5−7 min, 5% to 100% B; 7−8 min, 100% B; 8−8.1 min, 100% to 5% B; and 8.1−10 min, 5%B.

Following UPLC separation, the MS/MS of metabolites was carried out on SCIEX as a high-resolution tandem mass spectrometer TripleTOF5600plus in a mode of information-dependent acquisition (IDA), with the setting of the TOF mass range (60−1,200 Da) and survey scan of 150 ms. In each cycle (0.56 s), the 12 most intensive precursor ions (>100 counts/s intensity) in survey scan were chosen for MS/MS. The following ESI sources were set, including curtain gas (30 psi), Gas 1 (60 psi), Gas 2 (60 psi), interface heater temperature (650 °C), ion spray voltage floating (ISVF) (5000) and −4000 V for both modes (positive and negative),

correspondingly. Calibration of the mass accuracy was done every 20 samples, and a QC sample was obtained following every 10 samples.

Untargeted metabolomic data pre-processing and annotation

The MS raw datasets were converted to mzXML format with MSConvert of Proteowizard [15] followed by pre-processing of peak selection and grouping, correction of retention time, 2nd peak grouping, and annotation of isotopes and adducts by the XCMS [16] and CAMERA [17] with metaX [18] toolbox in R (parameters, Tables S5 and S6). Individual ion was recognized by pairing retention time (RT) and *m*/*z* dataset. Peak intensities were documented, and a 3-D matrix was generated for each specimen type with arbitrarily assigned peak indices (retention time−*m*/*z* pairs), name of samples (observations) and ion intensities (variables). Peak (adduct) features were named with the following format M(*m*/*z*)T(RT) where *m*/*z* was rounded to integer and RT was rounded to second.

KEGG [19] (www.genome.jp/kegg/) and HMDB [20] (hmdb.ca) databases annotated the metabolites via matching the exact *m*/*z*, accepting differences < 10 ppm. Further identification and validation of the molecular formula were performed, via the measurements of isotopic distribution. An in-house fragment spectrum library validated the identification of metabolites as well.

Those detectable features (< 50% of QC or 80% of biological samples) were deleted, and the remaining ones were subjected to metaX for further pre-procession. The missing values in the datasets were imputed by the k-nearest neighbor algorithm. Principal component analysis (PCA) was undertaken with the pre-processed data to detect outliers based on expansion of the Hotelling's T2 distribution ellipse [21] and assess batch effects. QC robust LOESS signal correction was fitted to the QC dataset regarding the injection order for minimizing the drift of signal intensity along the timeline.

Supplementary text

Results

Microbiome raw data processing with DADA2 pipeline

The raw data was processed using DADA2 pipeline, with denoising, merging and chimera-

removing. The summary of the generated final chimera-free dataset was shown in the table below and the distribution of library size and rarecurves were shown in the figures below, revealing adequate sequencing depth.

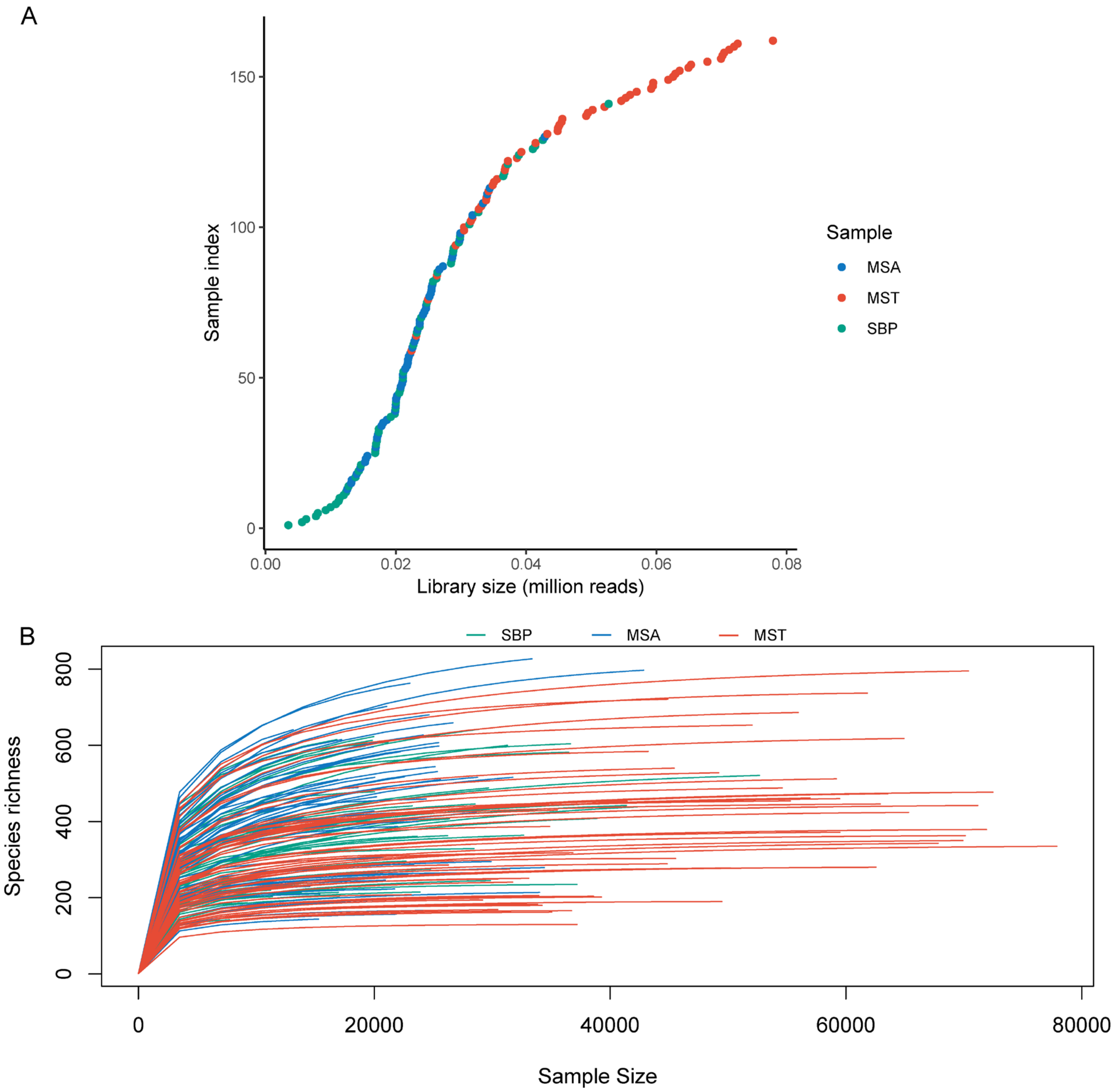

Figure (A) Distribution of library size of samples of the study. The data points are colored with sample types. (B) Rarecurves of all samples, plotted using rarecurve function of vegan package.

Table DADA2 pipeline summary

Sample ID	input	filtered	denoisedF	denoisedR	merged	nonchim
**GCF.T1.01**	94621	79095	76394	76354	40139	29687
**GCF.T1.02**	64613	59315	56408	58635	26394	25709
**GCF.T1.03**	77060	60477	73386	76061	39567	36636

**GCF.T1.04**	60348	53399	52631	52870	11794	11214
**GCF.T1.05**	74311	61529	52959	60593	14773	14641
**GCF.T1.06**	82462	67613	62652	65573	26534	23226
**GCF.T1.09**	73052	59167	50486	58253	20508	20031
**GCF.T1.10**	70029	61256	56260	60245	5759	5598
**GCF.T1.11**	64602	56066	55633	55625	17890	16972
**GCF.T1.13**	71520	63557	63091	63164	12683	11368
**GCF.T1.14**	57437	46244	42655	45791	22681	20673
**GCF.T1.16**	74776	63843	63341	63354	38058	37184
**GCF.T1.17**	55836	49480	44596	48641	8239	8022
**GCF.T1.18**	97060	71571	70890	70990	17851	17382
**GCF.T1.19**	54991	49598	47606	49113	32407	28468
**GCF.T1.21**	97227	72298	60785	71221	14806	14294
**GCF.T1.22**	97311	83175	79454	80729	37251	28877
**GCF.T1.23**	61101	54140	51687	53656	13978	13815
**GCF.T1.24**	87854	75902	68628	74878	28165	26379
**GCF.T1.25**	72057	63558	62290	63127	9358	9232
**GCF.T1.26**	95865	78556	75878	77260	24960	23641
**GCF.T1.27**	87501	65312	63485	64706	11179	10778
**GCF.T1.28**	56420	34383	29646	33318	6306	6231
**GCF.T1.29**	68887	62204	61291	61259	34658	32678
**GCF.T1.30**	92156	82225	75253	81269	42458	38889
**GCF.T1.31**	67091	61078	60170	60672	22232	21074
**GCF.T1.32**	58101	52276	49570	51671	20904	20542
**GCF.T1.33**	98000	74862	72884	74124	17588	17079
**GCF.T1.34**	68755	58289	52656	57779	30454	29860
**GCF.T1.35**	93573	82979	78831	81965	23542	22702
**GCF.T1.36**	83425	65215	50086	63615	12498	11997
**GCF.T1.39**	96970	70465	67520	69581	25427	24774
**GCF.T1.40**	59617	53065	52194	52295	13423	12752
**GCF.T1.41**	80267	60241	57998	59309	10297	9978
**GCF.T1.42**	61953	51796	50485	51268	18360	17394
**GCF.T1.44**	87770	73351	70516	71840	17550	12350
**GCF.T1.45**	86042	72543	71312	71144	30039	28548
**GCF.T1.46**	62566	55712	54581	54727	24995	23893
**GCF.T1.47**	90916	77824	74879	75082	21660	16823
**GCF.T1.49**	98875	78704	74831	77252	17965	16856
**GCF.T1.50**	80574	67819	64648	64613	25188	19962
**GCF.T1.51**	88464	75497	72581	72709	28804	19835
**GCF.T1.52**	87470	75859	74376	73937	51982	42551
**GCF.T1.55**	89732	75623	71848	73025	21631	19230
**GCF.T1.56**	87220	81772	80281	80282	57596	52666
**GCF.T1.57**	87208	77804	72852	76295	45229	41383
**GCF.T1.58**	82940	75019	70765	73985	27895	26222
**GCF.T1.59**	91664	77407	75477	76717	3665	3496
**GCF.T1.60**	92408	83286	81784	81851	44633	41026

**GCF.T1.61**	98921	90613	87722	88518	38053	31303
**GCF.T1.62**	73132	68833	68243	68036	29683	28806
**GCF.T1.63**	70609	65660	64201	63963	21865	21164
**GCF.T1.64**	62858	42443	41716	41484	8328	7736
**GCF.T1.65**	69835	65693	64649	64502	37479	36491
**MSA.T1.01**	89987	80877	77463	78431	36960	24167
**MSA.T1.02**	76320	69249	68394	68525	24621	21450
**MSA.T1.03**	85007	79154	76223	77285	47831	42850
**MSA.T1.04**	69031	64044	63554	63523	19173	17805
**MSA.T1.05**	55515	50823	47550	50386	14799	14545
**MSA.T1.06**	80825	71940	67608	70378	21716	15623
**MSA.T1.09**	69904	64718	64112	64156	22317	20751
**MSA.T1.10**	69152	62558	61338	61857	30899	27225
**MSA.T1.11**	79675	71693	70177	71167	38294	34422
**MSA.T1.13**	69978	62720	61859	62189	39440	29914
**MSA.T1.14**	67373	62084	61485	61628	21865	21076
**MSA.T1.16**	77893	67380	61586	66793	22384	21983
**MSA.T1.17**	66487	61281	60001	60932	16526	15377
**MSA.T1.18**	95726	87322	84587	85537	47070	28762
**MSA.T1.19**	68931	63132	59391	62608	25070	24395
**MSA.T1.21**	84591	77143	75028	74853	38470	26680
**MSA.T1.22**	86129	78285	76099	76174	35245	20049
**MSA.T1.23**	76837	69926	69429	69328	28187	23670
**MSA.T1.24**	88062	80782	78617	78656	40728	25457
**MSA.T1.25**	67326	62619	62285	62084	23556	21872
**MSA.T1.26**	97304	89460	86600	87232	17615	13233
**MSA.T1.27**	81216	74990	73519	73525	29483	20186
**MSA.T1.28**	61094	55076	53957	54647	12872	12548
**MSA.T1.29**	67349	62629	62067	62176	32649	29818
**MSA.T1.30**	85850	78642	75481	74734	46281	33381
**MSA.T1.31**	68368	62370	61971	61899	26155	20947
**MSA.T1.32**	59322	55288	54949	54832	31256	21734
**MSA.T1.33**	94988	77204	65572	75139	15992	13141
**MSA.T1.34**	69940	63899	63384	63380	38835	34020
**MSA.T1.35**	86680	79283	76904	77509	22511	18047
**MSA.T1.36**	94031	85656	82254	83781	44999	25482
**MSA.T1.39**	80523	73254	71527	71828	28937	17114
**MSA.T1.40**	71993	63697	63270	63286	16493	15282
**MSA.T1.41**	82347	75447	73861	73588	38834	25155
**MSA.T1.42**	68679	60589	59909	60256	25263	24624
**MSA.T1.44**	89147	80446	77809	79010	43789	28665
**MSA.T1.45**	90424	78628	74895	75892	27108	23031
**MSA.T1.46**	74804	66518	63184	66092	24390	23344
**MSA.T1.47**	93995	84524	79439	82608	21327	16919
**MSA.T1.49**	93448	85254	83467	83238	48308	25350
**MSA.T1.50**	99339	91672	88389	89425	28748	21062

**MSA.T1.51**	96811	89022	86736	87294	42312	31759
**MSA.T1.52**	80695	74269	72452	72339	28200	19913
**MSA.T1.55**	95270	85544	78665	83452	31988	24654
**MSA.T1.56**	84533	78316	76216	76358	29448	22208
**MSA.T1.57**	85183	71484	67562	68931	20983	17236
**MSA.T1.58**	88586	80665	78156	79173	24879	19983
**MSA.T1.59**	81690	73512	70303	72131	18174	13995
**MSA.T1.60**	87105	78766	76160	77147	26727	22545
**MSA.T1.61**	90542	82921	81292	81033	41947	25626
**MSA.T1.62**	75094	64186	63316	63227	19434	18624
**MSA.T1.63**	71864	63532	62581	62805	24637	21845
**MSA.T1.64**	64403	57554	57037	57037	23613	22847
**MSA.T1.65**	70380	67176	66426	66386	24276	23664
**MST.T1.01**	94711	88537	85249	86169	66799	61816
**MST.T1.02**	66382	55044	54242	53894	41621	30477
**MST.T1.03**	67853	56881	55994	55760	43606	31742
**MST.T1.04**	59876	51381	50363	49872	39308	32738
**MST.T1.05**	56766	51894	51471	51259	41798	36730
**MST.T1.06**	80378	75737	73662	74180	62068	56959
**MST.T1.09**	57431	47713	46509	45997	35700	26301
**MST.T1.10**	60689	49749	48815	48082	34234	24946
**MST.T1.11**	67143	55594	54627	54375	48553	33105
**MST.T1.13**	70423	61887	61217	61112	56094	49482
**MST.T1.14**	61317	51693	50593	50535	40562	29184
**MST.T1.16**	60562	53020	52488	52350	47945	39265
**MST.T1.17**	63323	55892	55349	55259	45920	38593
**MST.T1.18**	80932	75273	73769	74468	68241	62551
**MST.T1.19**	65701	54570	53830	53484	46697	33945
**MST.T1.21**	89756	81964	80082	80607	59044	55292
**MST.T1.22**	90869	85933	84282	84527	43605	41447
**MST.T1.23**	57010	51927	51328	51337	45856	35061
**MST.T1.24**	92553	84942	82433	83708	70850	65311
**MST.T1.25**	61726	55186	54744	54625	41712	33853
**MST.T1.26**	83226	78477	76790	76827	65143	54606
**MST.T1.27**	81247	73173	70226	71825	51970	49217
**MST.T1.28**	53560	47416	46787	46834	36296	30475
**MST.T1.29**	55353	50429	49870	49916	43605	37199
**MST.T1.30**	85857	78564	75876	76990	66061	59187
**MST.T1.31**	67408	58069	56407	56213	43212	35532
**MST.T1.32**	58703	55089	53518	54406	46903	45558
**MST.T1.33**	99762	92698	90023	90124	75040	70396
**MST.T1.34**	52208	48570	48047	47842	41779	31495
**MST.T1.35**	83951	79914	78812	78947	46841	44838
**MST.T1.36**	86621	82536	81308	81479	36146	34874
**MST.T1.39**	93198	86594	84042	85614	76017	71915
**MST.T1.40**	60161	50215	48973	48543	30068	22401

**MST.T1.41**	99032	93927	92580	93141	53927	50207
**MST.T1.42**	62070	50490	49731	49455	38296	23133
**MST.T1.44**	92007	87360	85544	85638	74317	59498
**MST.T1.45**	95187	88088	85873	86467	68563	64917
**MST.T1.46**	56502	49850	49523	49246	40480	34235
**MST.T1.47**	99995	91429	89223	89932	78527	71170
**MST.T1.49**	93273	87508	84677	85754	55299	45114
**MST.T1.50**	82481	74306	71698	73221	63166	59469
**MST.T1.51**	50400	45658	44256	44861	40465	36848
**MST.T1.52**	81157	76504	75327	75284	64848	62910
**MST.T1.55**	99340	94077	92492	92870	78723	69916
**MST.T1.56**	89951	84237	82880	83032	75762	70129
**MST.T1.57**	88518	81806	78508	80503	68507	63554
**MST.T1.58**	98367	92267	89992	91191	80215	77882
**MST.T1.59**	82423	75722	72742	72522	59213	55959
**MST.T1.60**	97401	91876	89841	90264	76796	72474
**MST.T1.61**	85843	81004	79473	79967	71353	67821
**MST.T1.62**	72952	68279	67255	66910	47798	45436
**MST.T1.63**	70833	67589	66806	66345	57095	52033
**MST.T1.64**	65142	61424	60319	60069	48136	44896
**MST.T1.65**	72915	67673	66279	66171	50665	43238

Discussion

The Global Burden of Disease (GBD) study 2017 shows high and increasing rates of severe periodontitis in mainland China from 1990 to 2017, especially among younger ages [22]. This study in Shenzhen metropolitan investigates the impacts of maternal periodontitis in first-trimester pregnant women on the microbiome and metabolome profiles. Considering dramatically narrowed regional urban-rural disparities of periodontal [23], maternal and child conditions [24] along with the socio-economic progress, we assume unsubstantial regional bias included.

References

1. Li HJ, Zhao D, Xu X, Yu R, Zhang F, Cheng T, Zheng Z, Yang H, Yang C, Yao J, et al. Diagnostic performance of the AAP/EFP classification and the CDC/AAP case definition among pregnant women and a practical screening tool for maternal periodontal diseases**.** J Periodontal Res. 2022;57**:**960-968.
2. Zhang F, Zhao D, Xu X, Wen P, Li H, Yu R, Cheng T, Zheng Z, Yang H, Yang C, et al. Periodontitis links to concurrent metabolic disorders and abnormal liver function in pregnant women**.** Oral Dis. 2024;30**:**697-709.

3. Tonetti MS, Greenwell H, Kornman KS. Staging and grading of periodontitis: Framework and proposal of a new classification and case definition. J Clin Periodontol. 2018;45:S149-S161.
4. Love MI, Huber W, Anders S. Moderated estimation of fold change and dispersion for RNA-seq data with DESeq2. Genome Biol. 2014;15:550.
5. Cao Y, Dong Q, Wang D, Zhang P, Liu Y, Niu C. microbiomeMarker: an R/Bioconductor package for microbiome marker identification and visualization. Bioinformatics. 2022;38:4027-4029.
6. Lin H, Peddada SD. Multigroup analysis of compositions of microbiomes with covariate adjustments and repeated measures. Nat Methods. 2024;21:83-91.
7. Rohart F, Gautier B, Singh A, Lê Cao KA. mixOmics: An R package for 'omics feature selection and multiple data integration. PLoS Comput Biol. 2017;13:e1005752.
8. Breiman L. Random forests. Machine Learning. 2001;45:5-32.
9. Kursa MB, Rudnicki WR. Feature selection with the Boruta package. J Stat Softw. 2010;36:1-13.
10. Singh A, Shannon CP, Gautier B, Rohart F, Vacher M, Tebbutt SJ, Le Cao KA. DIABLO: an integrative approach for identifying key molecular drivers from multi-omics assays. Bioinformatics. 2019;35:3055-3062.
11. Lin H, Eggesbo M, Peddada SD. Linear and nonlinear correlation estimators unveil undescribed taxa interactions in microbiome data. Nat Commun. 2022;13:4946.
12. Lu Y, Zhou G, Ewald J, Pang Z, Shiri T, Xia J. MicrobiomeAnalyst 2.0: comprehensive statistical, functional and integrative analysis of microbiome data. Nucleic Acids Res. 2023, 10.1093/nar/gkad407.
13. Shannon P, Markiel A, Ozier O, Baliga NS, Wang JT, Ramage D, Amin N, Schwikowski B, Ideker T. Cytoscape: a software environment for integrated models of biomolecular interaction networks. Genome Res. 2003;13:2498-2504.
14. Dunn WB, Broadhurst D, Begley P, Zelena E, Francis-McIntyre S, Anderson N, Brown M, Knowles JD, Halsall A, Haselden JN, et al. Procedures for large-scale metabolic profiling of serum and plasma using gas chromatography and liquid chromatography coupled to mass spectrometry. Nat Protoc. 2011;6:1060-1083.
15. Chambers MC, Maclean B, Burke R, Amodei D, Ruderman DL, Neumann S, Gatto L, Fischer B, Pratt B, Egertson J, et al. A cross-platform toolkit for mass spectrometry and proteomics. Nat Biotechnol. 2012;30:918-920.
16. Smith CA, Want EJ, O'Maille G, Abagyan R, Siuzdak G. XCMS: processing mass spectrometry data for metabolite profiling using nonlinear peak alignment, matching, and identification. Anal Chem. 2006;78:779-787.
17. Kuhl C, Tautenhahn R, Bottcher C, Larson TR, Neumann S. CAMERA: an integrated strategy for compound spectra extraction and annotation of liquid chromatography/mass spectrometry

data sets. Anal Chem. 2012;84:283-289.

18. Wen B, Mei Z, Zeng C, Liu S. metaX: a flexible and comprehensive software for processing metabolomics data. BMC Bioinformatics. 2017;18:183.

19. Kanehisa M, Goto S. KEGG: Kyoto Encyclopedia of Genes and Genomes. Nucleic Acids Res. 2000;28:27-30.

20. Wishart DS, Guo A, Oler E, Wang F, Anjum A, Peters H, Dizon R, Sayeeda Z, Tian S, Lee BL, et al. HMDB 5.0: the Human Metabolome Database for 2022. Nucleic Acids Res. 2022;50:D622-D631.

21. Edmands WM, Barupal DK, Scalbert A. MetMSLine: an automated and fully integrated pipeline for rapid processing of high-resolution LC-MS metabolomic datasets. Bioinformatics. 2015;31:788-790.

22. Luo LS, Luan HH, Wu L, Shi YJ, Wang YB, Huang Q, Xie WZ, Zeng XT. Secular trends in severe periodontitis incidence, prevalence and disability-adjusted life years in five Asian countries: A comparative study from 1990 to 2017. J Clin Periodontol. 2021;48:627-637.

23. Jiao J, Jing W, Si Y, Feng X, Tai B, Hu D, Lin H, Wang B, Wang C, Zheng S, et al. The prevalence and severity of periodontal disease in Mainland China: Data from the Fourth National Oral Health Survey (2015-2016). J Clin Periodontol. 2021;48:168-179.

24. Qiao J, Wang Y, Li X, Jiang F, Zhang Y, Ma J, Song Y, Ma J, Fu W, Pang R, et al. A Lancet Commission on 70 years of women's reproductive, maternal, newborn, child, and adolescent health in China. Lancet. 2021;397:2497-2536.

Supplementary figures

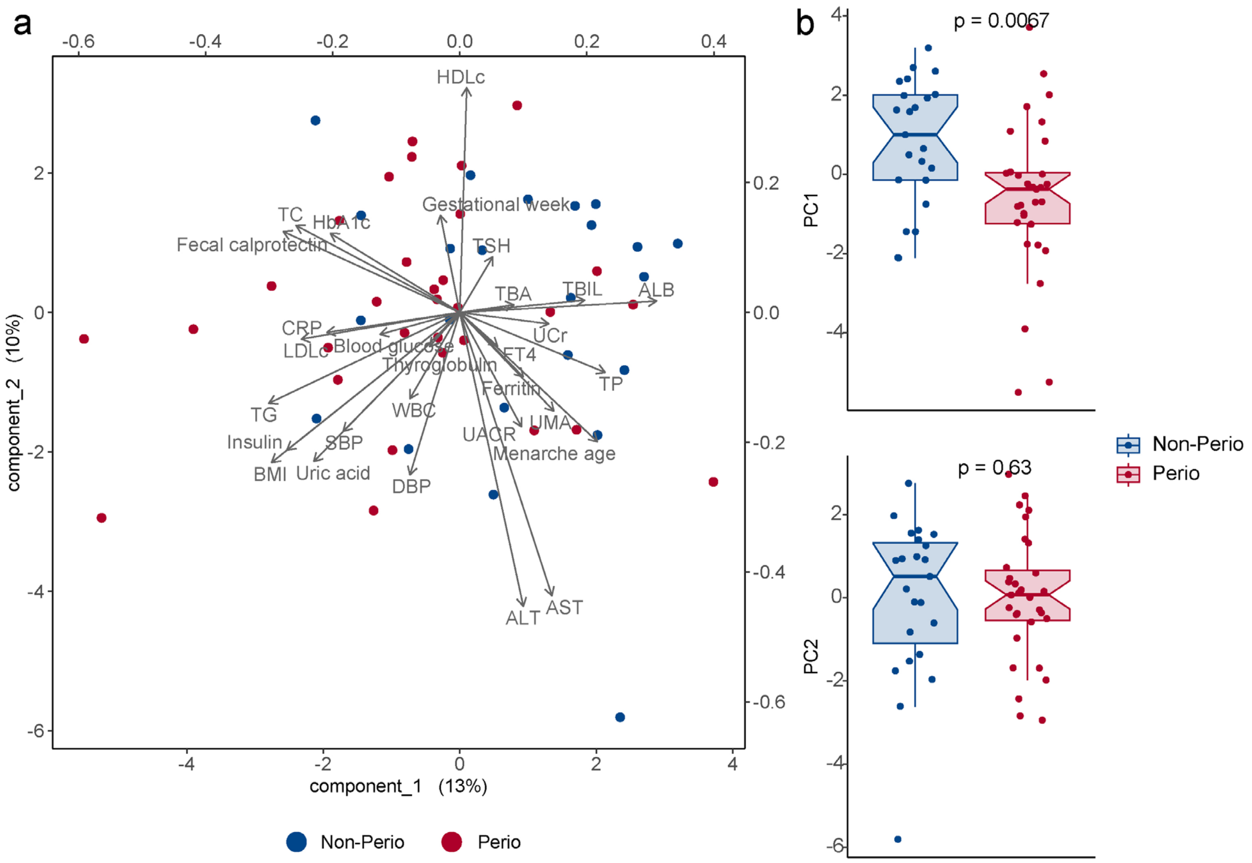

Figure S1. Principal component analysis of systemic clinical parameters between Perio (n = 31) and Non-Perio (n = 23) groups.

(A**)** Principal component analysis biplot. (B) Wilcoxon rank sum test of two components.

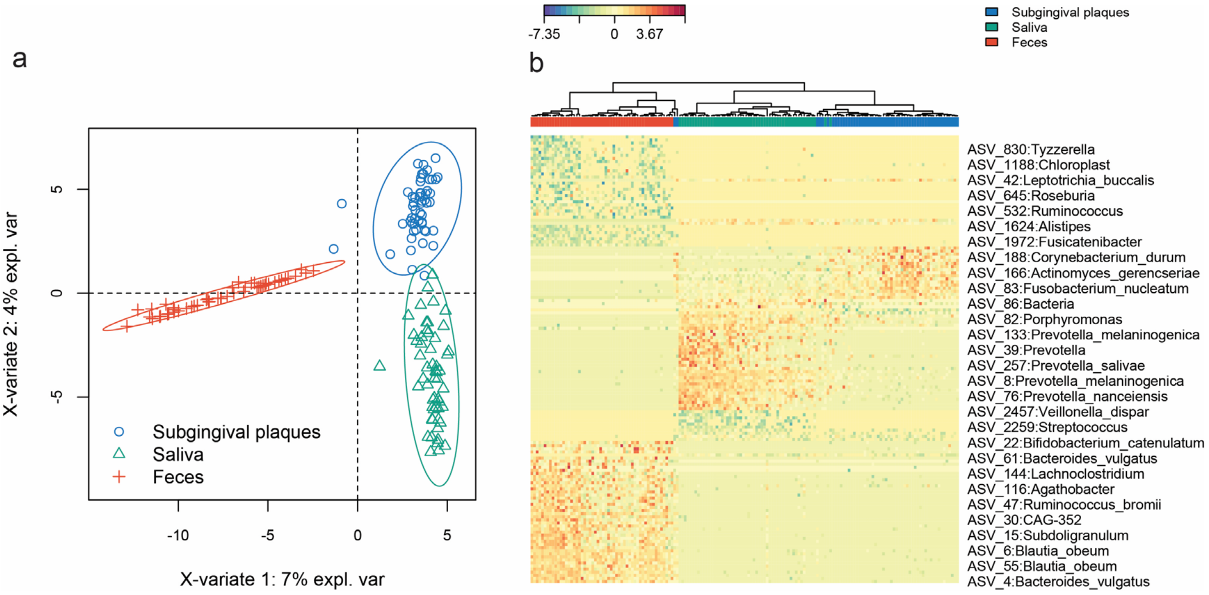

Figure S2. MixMC analysis shows distinct microbiota from subgingival plaque, saliva, and feces. (A**)** sPLS-DA projected plot of the first and second components of the three types of samples. (B**)** Clustered heatmap of MixMC-selected ASV features from the and second components of the three types of samples after robust centered log-ratio transformation.

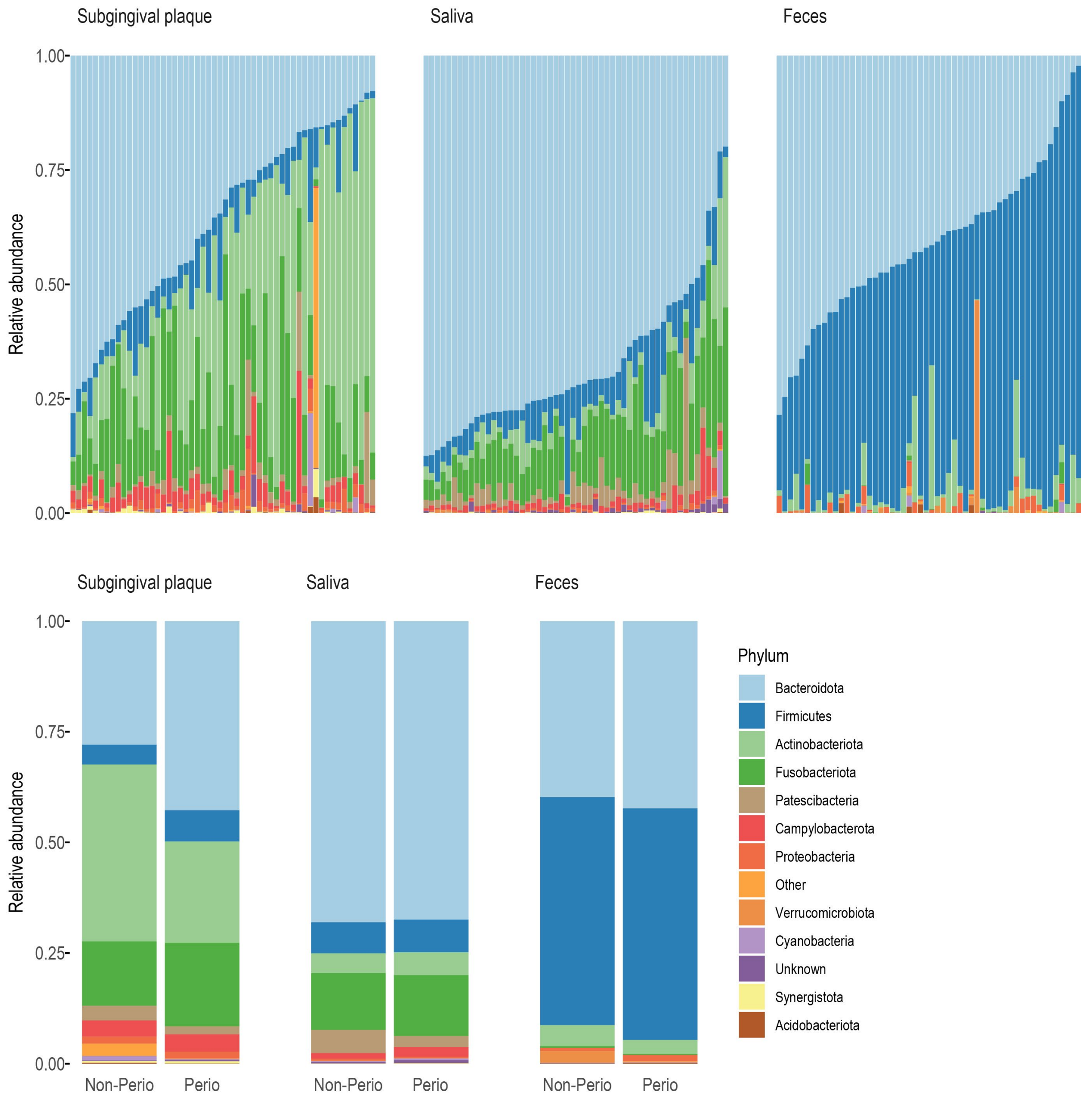

Figure S3. Relative abundance of phyla among the microbiota from subgingival plaque, saliva, and feces.

(A**)** Individual bar plot of phyla with descending Bacteroidota. (B**)** Average relative abundance of phyla of Non-Perio and Perio groups. Detection threshold: 0.01; prevalence threshold: 1%.

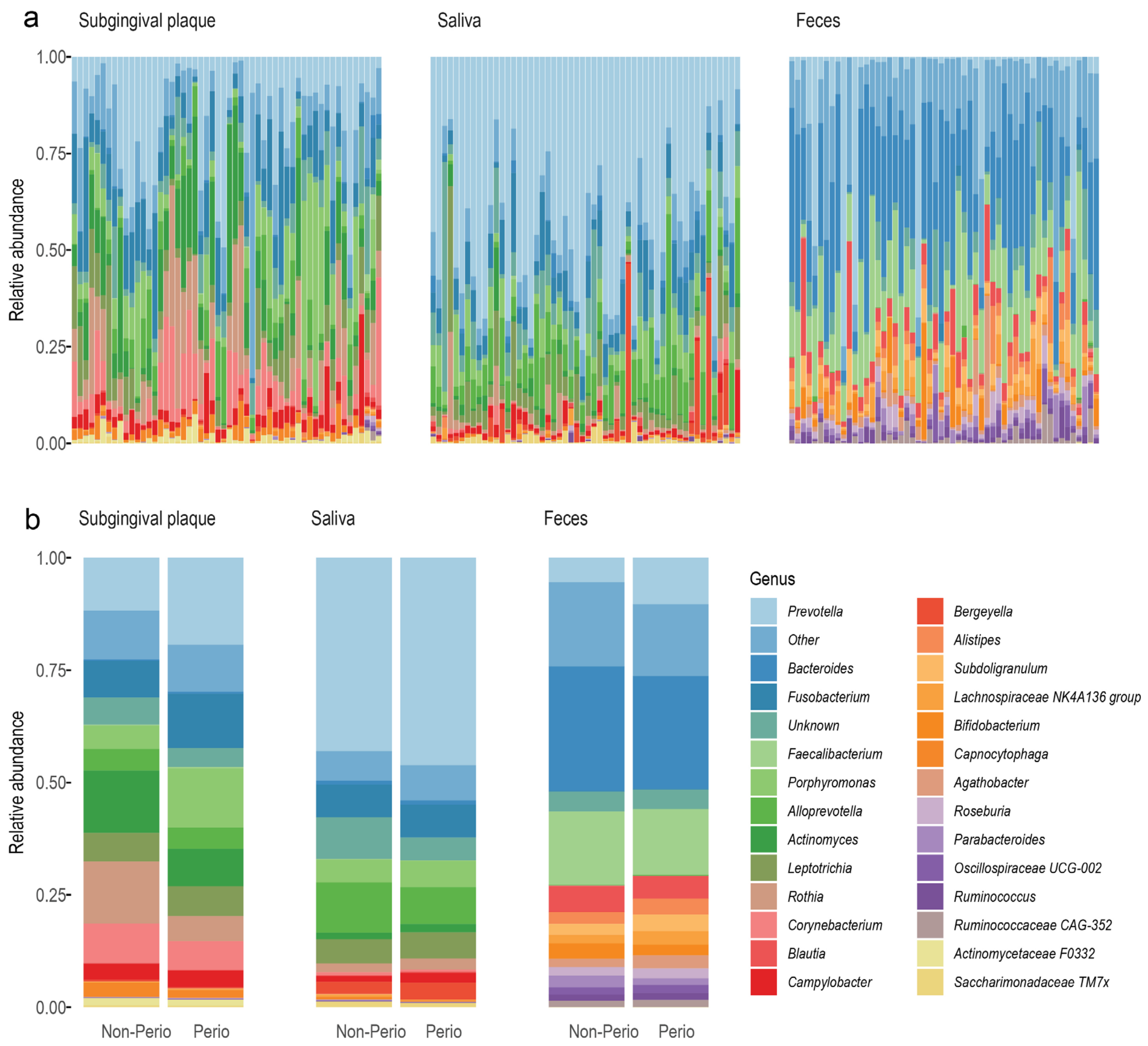

Figure S4. Relative abundance of genera among the microbiota from subgingival plaque, saliva, and feces.

(A**)** Individual bar plot of genera. (B**)** Average relative abundance of genera of Non-Perio and Perio groups. Detection threshold: 0.01; prevalence threshold: 10%.

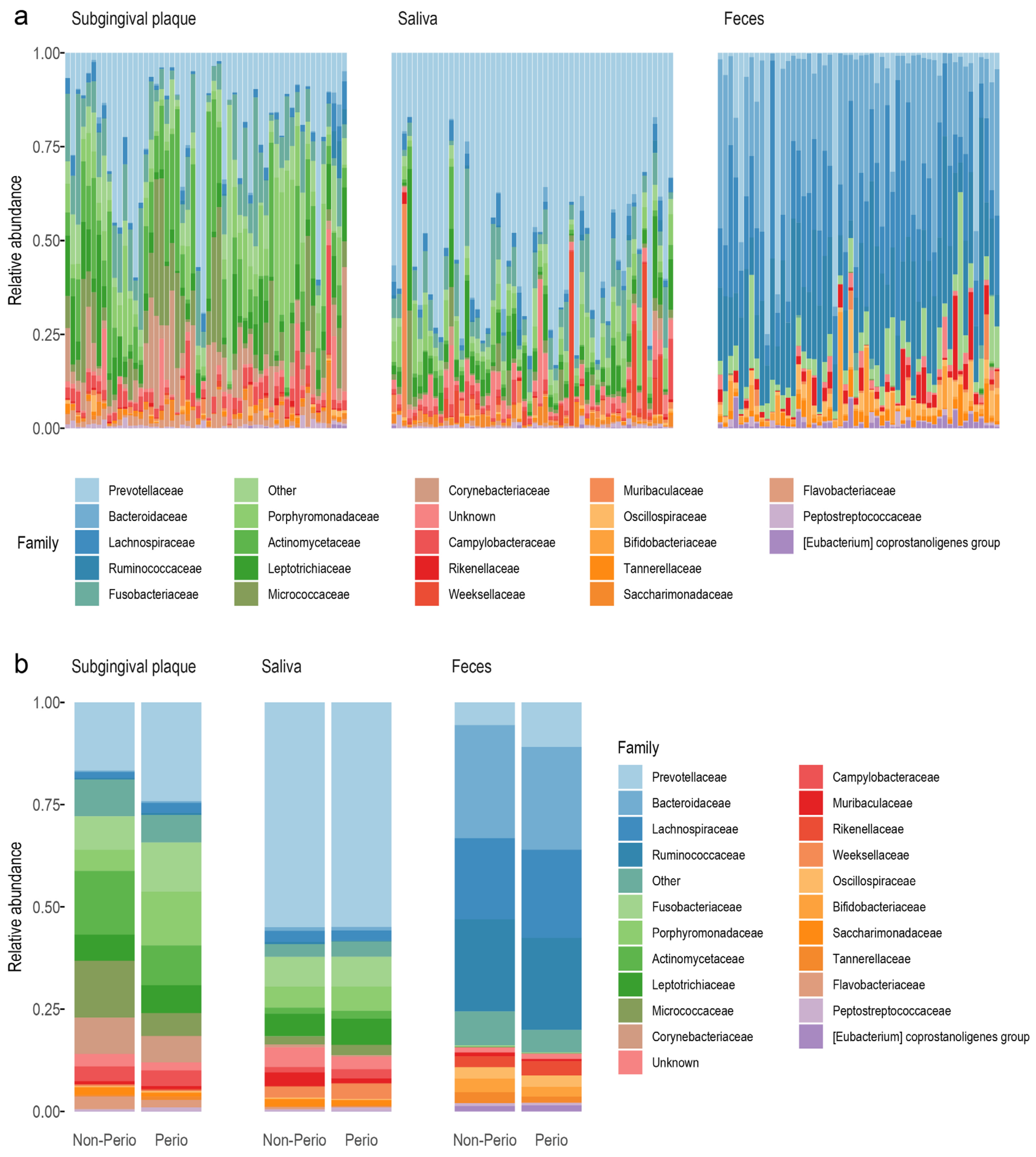

Figure S5. Relative abundance of families among the microbiota from subgingival plaque, saliva, and feces.

(A**)** Individual bar plot of families. (B**)** Average relative abundance of families of Non-Perio and Perio groups. Detection threshold: 0.01; prevalence threshold: 10%.

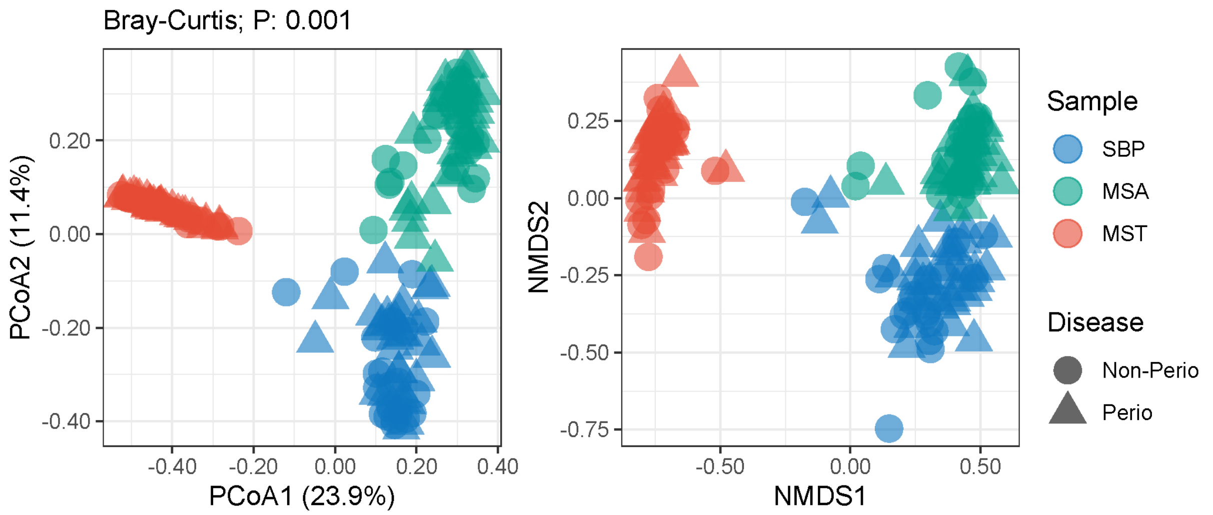

Figure S6. Beta diversity of microbiota from subgingival plaque, saliva, and feces.
PCoA and NMDS plots with Bray-Curtis dissimilarity. Overall PERMANOVA by sample types were significant ($P = 0.001$).

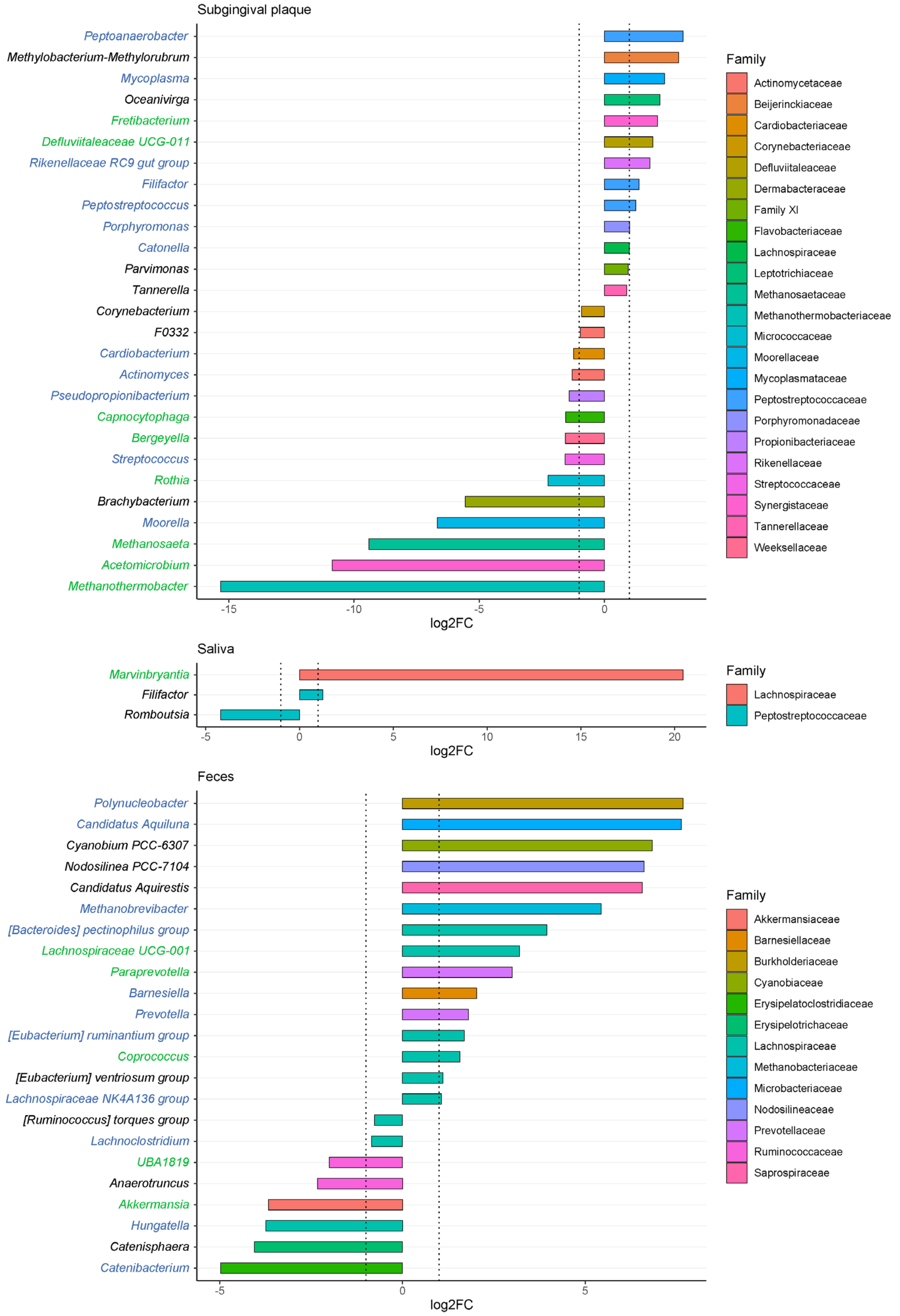

Figure S7. DESeq2-selected significantly differentially abundant genera in subgingival plaques, saliva, and feces between Perio (n = 31) and Non-Perio (n = 23) groups.

P values from Wald test (two-sided) implemented with DESeq2 with incorporating BMI covariate, with Benjamini-Hochberg adjustment for multiple comparisons. Green labels of genera: FDR < 0.05; blue: FDR < 0.1. Detailed lists are provided in Table S9.

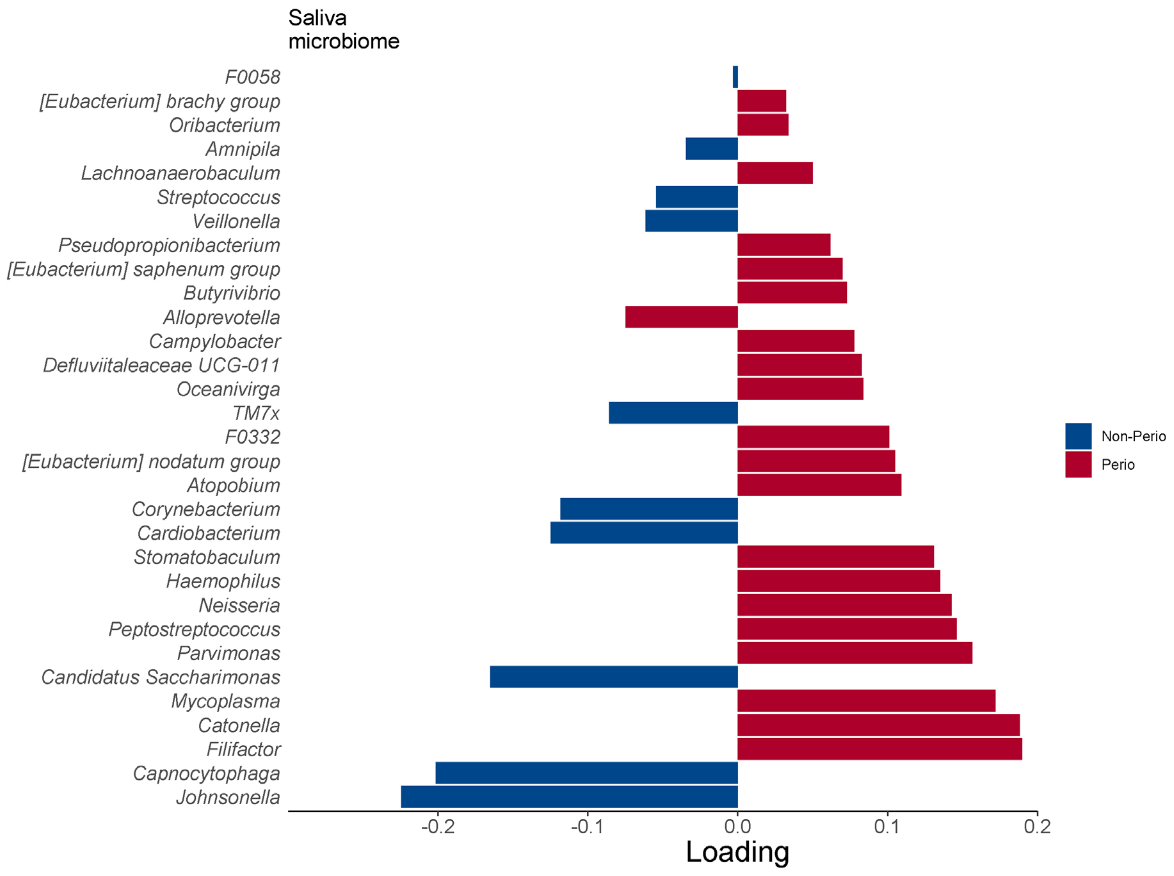

Figure S8. sPLS-DA-selected important genera in saliva between Perio (n = 31) and Non-Perio (n = 23) groups.

Selected genera in component 1. Genera of "tie" were not shown. The x-axis represents the loading importance.

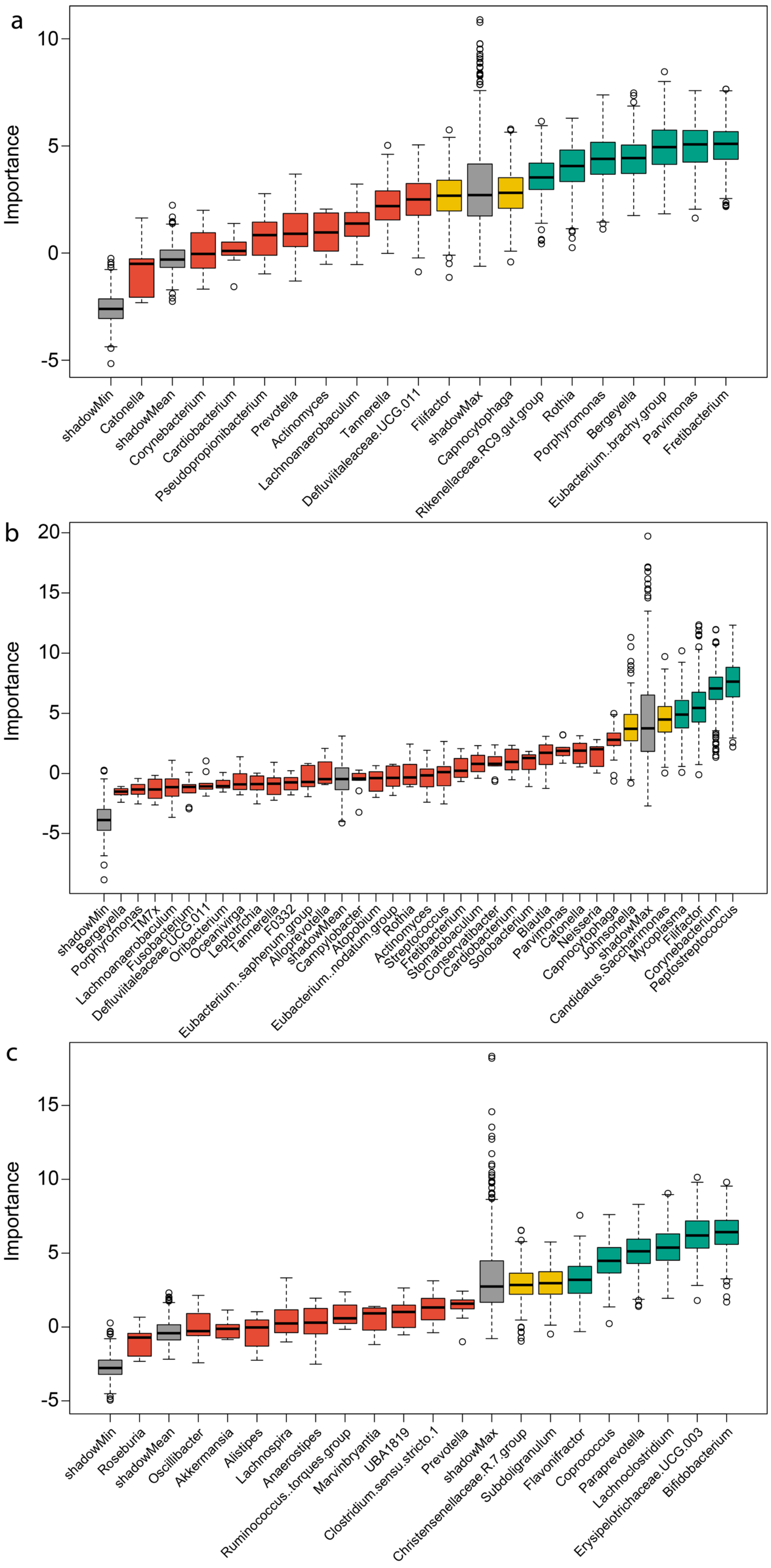
a
b
c
Importance
shadowMin
Catonella
shadowMean
Corynebacterium
Cardiobacterium
Pseudopropionibacterium
Prevotella
Actinomyces
Lachnoanaerobaculum
Tannerella
Defluviitaleaceae.UCG.011
Filifactor
shadowMax
Capnocytophaga
Rikenellaceae.RC9.gut.group
Rothia
Porphyromonas
Bergeyella
Eubacterium..brachy.group
Parvimonas
Fretibacterium
Bergeyella
Porphyromonas
TM7x
Lachnoanaerobaculum
Fusobacterium
Defluviitaleaceae.UCG.011
Oribacterium
Oceanivirga
Leptotrichia
Tannerella
F0332
Eubacterium..saphenum.group
Alloprevotella
shadowMean
Campylobacter
Atopobium
Eubacterium..nodatum.group
Rothia
Actinomyces
Streptococcus
Fretibacterium
Stomatobaculum
Conservatibacter
Cardiobacterium
Solobacterium
Blautia
Parvimonas
Catonella
Neisseria
Capnocytophaga
Johnsonella
shadowMax
Candidatus.Saccharimonas
Mycoplasma
Filifactor
Corynebacterium
Peptostreptococcus
Roseburia
Oscillibacter
Akkermansia
Alistipes
Lachnospira
Anaerostipes
Ruminococcus..torques.group
Marvinbryantia
UBA1819
Clostridium.sensu.stricto.1
Prevotella
Christensenellaceae.R.7.group
Subdoligranulum
Flavonifractor
Coprococcus
Paraprevotella
Lachnoclostridium
Erysipelotrichaceae.UCG.003
Bifidobacterium

Figure S9. Boruta-selected important genera for classification between Perio (n = 31) and Non-Perio (n = 23) groups.

Variable importance plot for classification performance determined using a Boruta feature selection algorithm on three types of samples. Selected genera in (A**)** subgingival plaques, (B**)** saliva, and (C**)** feces. Gray boxplots correspond to minimum, average, and maximum of Z-score of the mean decrease accuracy (importance) of shadow attributes. Red, yellow, and green boxplots represent the importance of respectively rejected and confirmed attributes. The boxplots with whiskers represent the median, the first and third quartiles of the data. Outliers are shown as circles.

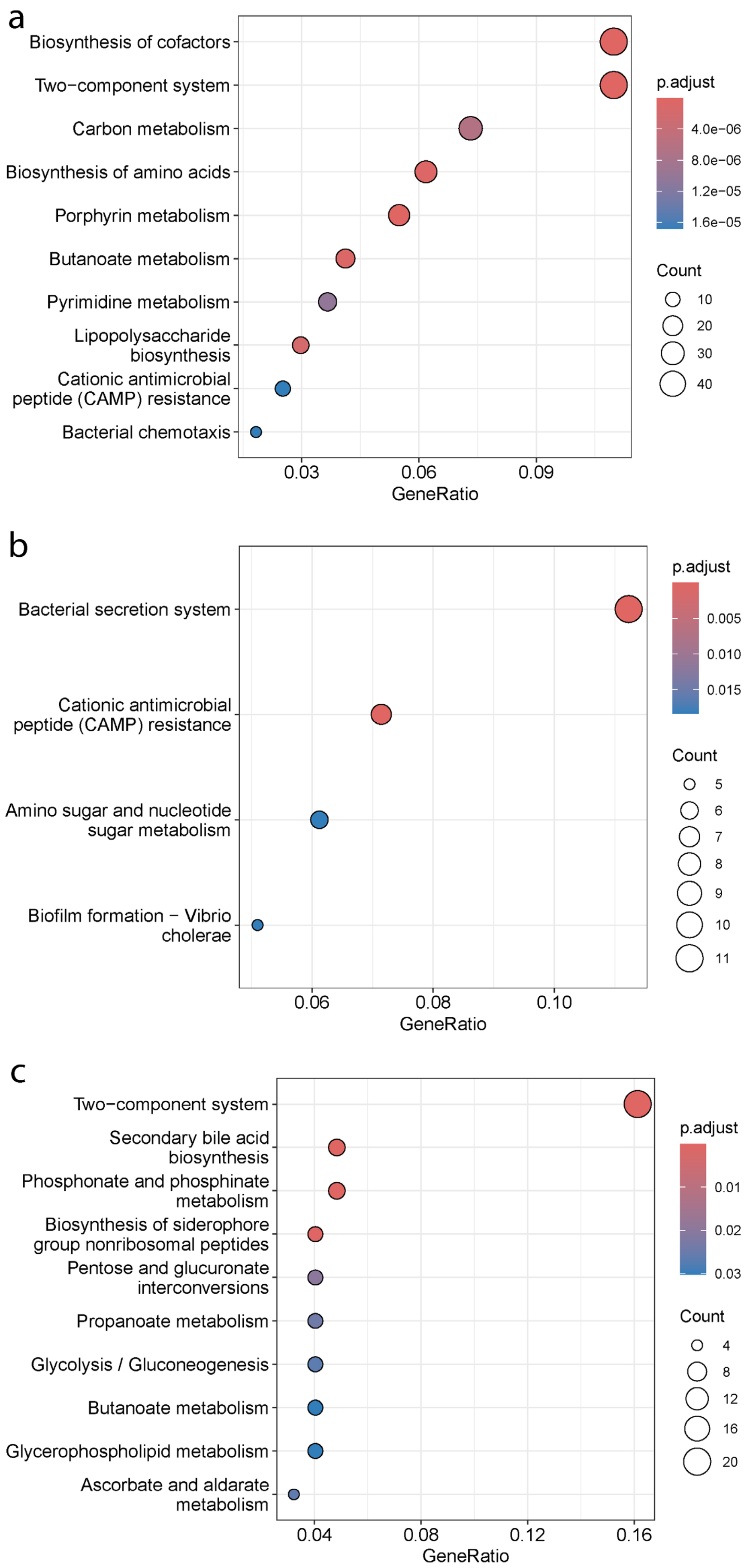
a
Biosynthesis of cofactors
Two−component system
Carbon metabolism
Biosynthesis of amino acids
Porphyrin metabolism
Butanoate metabolism
Pyrimidine metabolism
Lipopolysaccharide biosynthesis
Cationic antimicrobial peptide (CAMP) resistance
Bacterial chemotaxis
0.03
0.06
0.09
GeneRatio
p.adjust
4.0e−06
8.0e−06
1.2e−05
1.6e−05
Count
10
20
30
40
b
Bacterial secretion system
Cationic antimicrobial peptide (CAMP) resistance
Amino sugar and nucleotide sugar metabolism
Biofilm formation − Vibrio cholerae
0.06
0.08
0.10
GeneRatio
p.adjust
0.005
0.010
0.015
Count
5
6
7
8
9
10
11
c
Two−component system
Secondary bile acid biosynthesis
Phosphonate and phosphinate metabolism
Biosynthesis of siderophore group nonribosomal peptides
Pentose and glucuronate interconversions
Propanoate metabolism
Glycolysis / Gluconeogenesis
Butanoate metabolism
Glycerophospholipid metabolism
Ascorbate and aldarate metabolism
0.04
0.08
0.12
0.16
GeneRatio
p.adjust
0.01
0.02
0.03
Count
4
8
12
16
20

Figure S10. KEGG Ortholog enrichment of significant functional KOs predicted by PICRUSt2. PICRUSt2-predicted KOs were investigated with their dependence to periodontal status between Perio (n = 31) and Non-Perio (n = 23) groups. Significantly differentially abundant KO terms were subjected to MicrobiomeProfiler for enrichment analysis. Dot plots of top 10 enriched pathways in (A**)** subgingival plaque, (B**)** saliva, and (C**)** feces. Detailed lists are provided in Table S14.

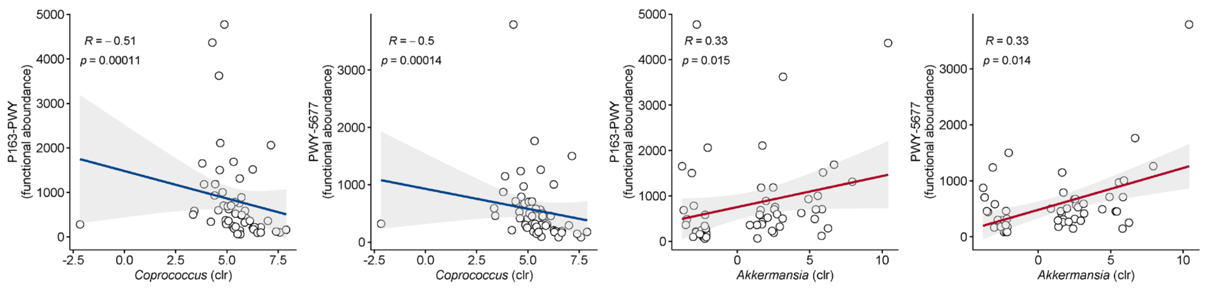

Figure S11. Fecal genera correlation with PICRUSt2-predicted pathways.

Scatter plots of Spearman's correlation between selected fecal genera *Coprococcus* and *Akkermansia* and two butyrate pathways.

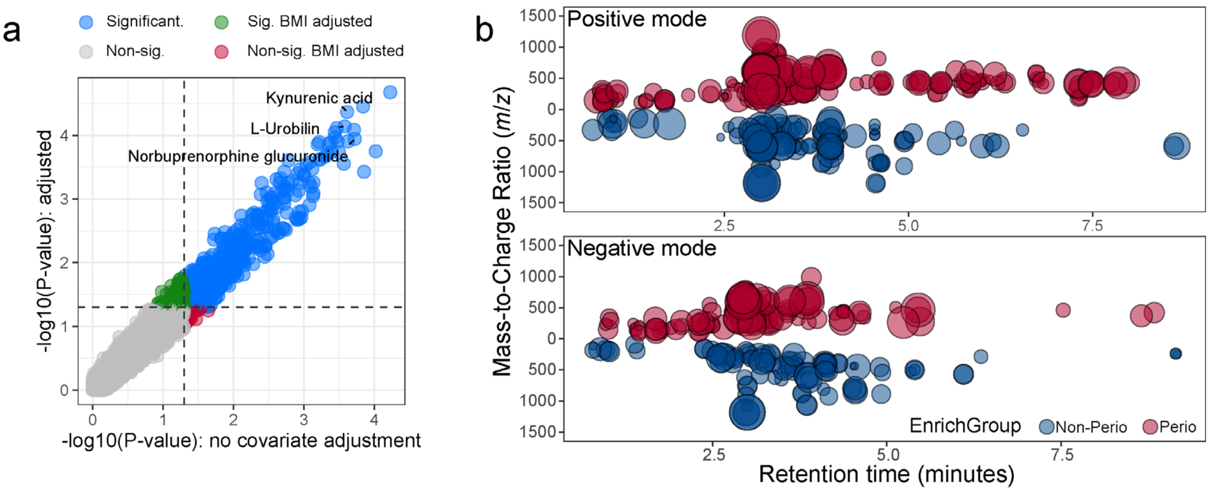

Figure S12. limma analysis of fecal metabolome.

(A) Differentially changed metabolic features in feces between the two groups were identified using limma analysis with and without adjustment of BMI (P < 0.05). Three MS2- and HMDB-annotated features with FDR < 0.05 were labelled. 448 significant features; 99 significant features when BMI adjusted; and 41 non-significant features when BMI adjusted. (B) Distribution of retention time and mass-to-charge ratio of limma-identified and BMI-adjusted differentially changed (P < 0.05) metabolic features in feces (n=547) of both positive and negative modes in cloud plots. The circle size represents the absolute $\log_2$FC.

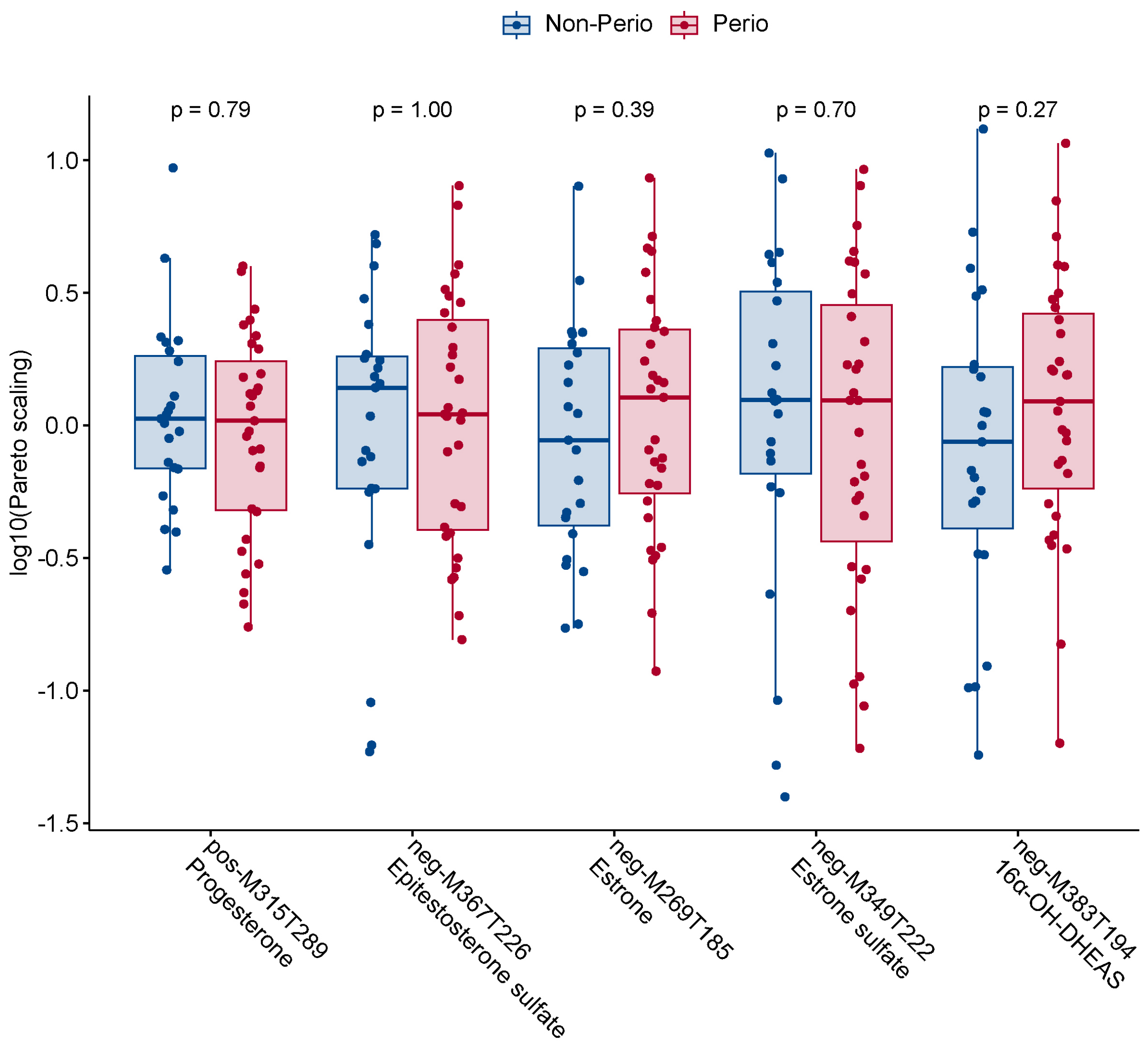

Figure S13. Serum sex hormones.

Boxplots of detected metabolic features for sex hormones in serum between Perio (n = 31) and Non-Perio (n = 23) groups. Wilcoxon rank sum test.

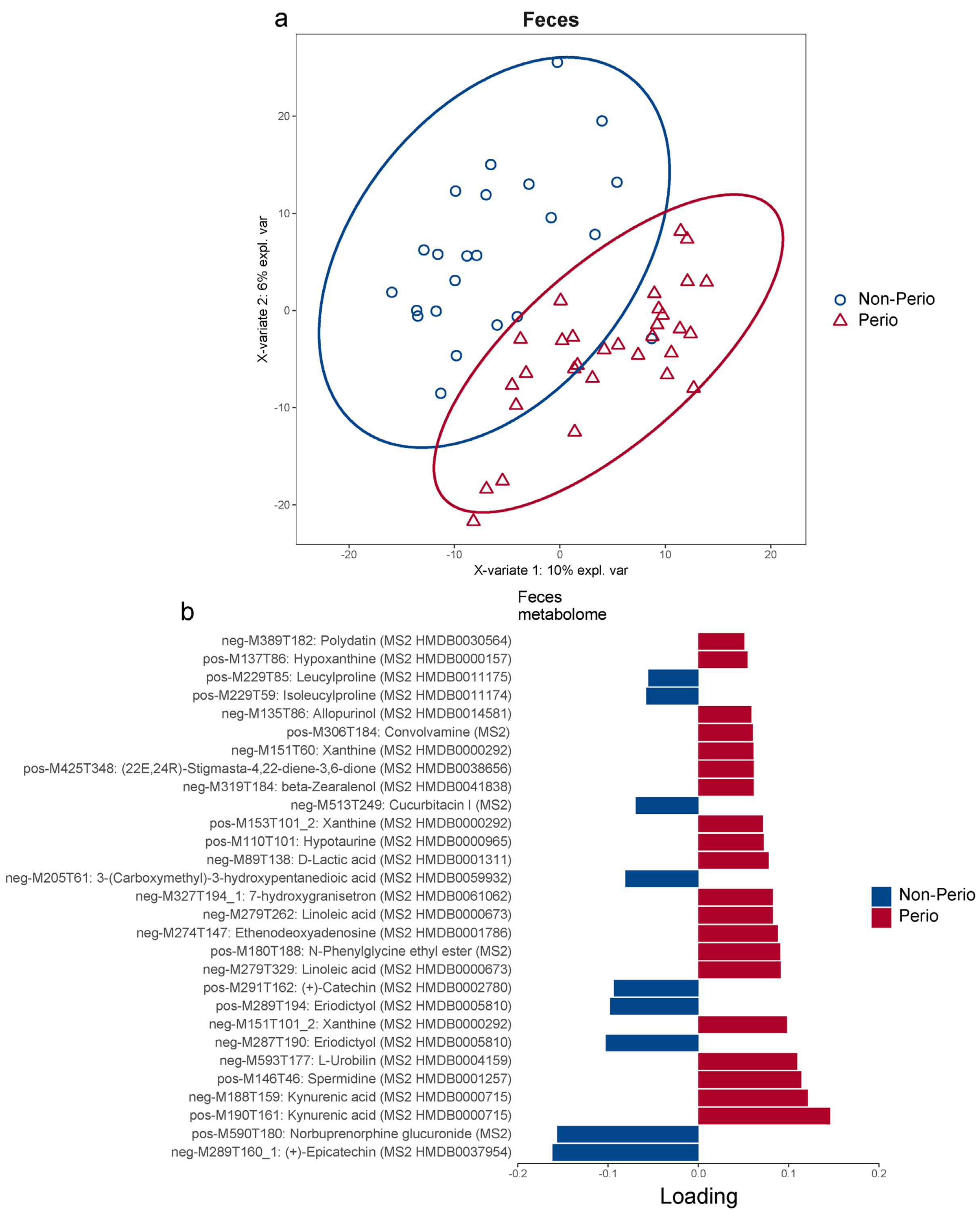

Figure S14. sPLS-DA-selected important metabolic features in feces of pregnant women. (A**)** sPLS-DA plot of the final model of metabolic features in feces between Perio (n = 31) and Non-Perio (n = 23) groups. (B**)** Selected MS2-annotated metabolic features in component 1 with loading importance larger than 0.05 were shown for conciseness. When no HMDB ID was presented, the feature was annotated by an in-house library only. The x-axis represents the loading importance. A detailed list is provided in Table S25.

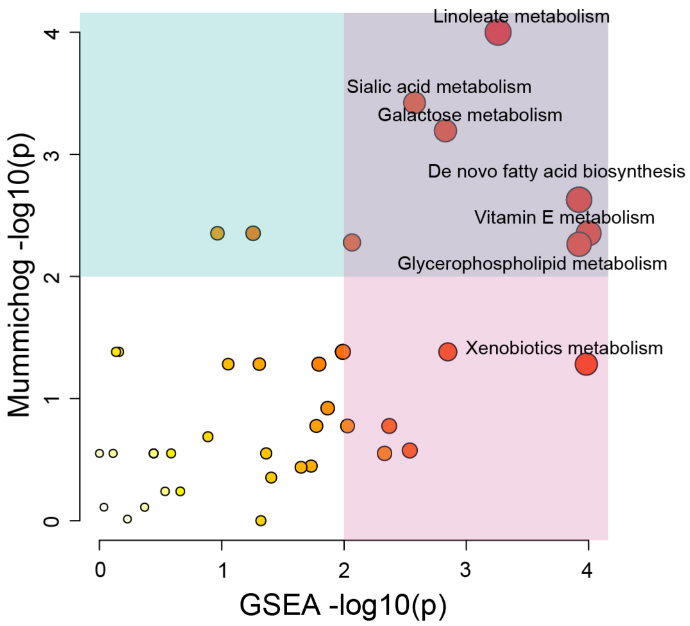

Figure S15. Functional enrichment analysis on sPLS-DA-selected metabolic features in feces (n = 300) with integration of mummichog and GSEA algorithms.

mummichog was set with *P*-value cutoff of 0.01. The enrichment was performed against human MFN pathway library implemented in MetaboAnalystR. Detailed result is provided in Table S26.

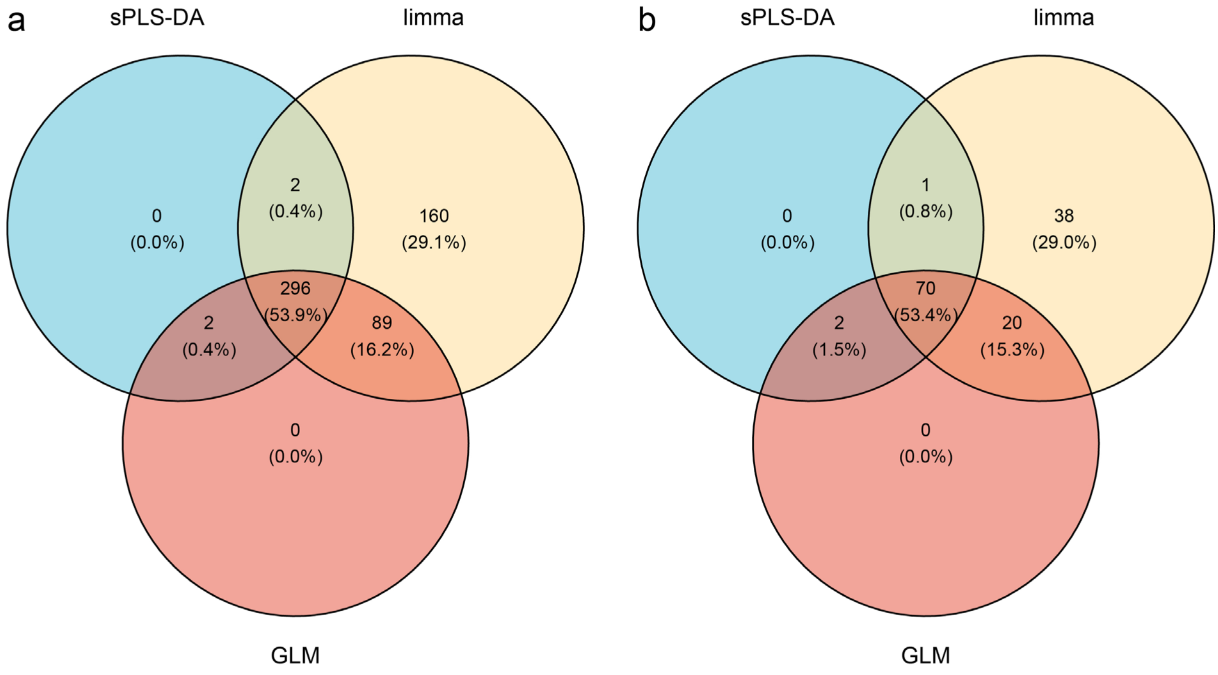

Figure S16. Venn plots of periodontitis-enriched metabolic features from limma, sPLS-DA, and GLM methods.

(A**)** Venn plot of all metabolic features. (B**)** Venn plot of MS2-annotated metabolic features. A detailed list is provided in Table S27.

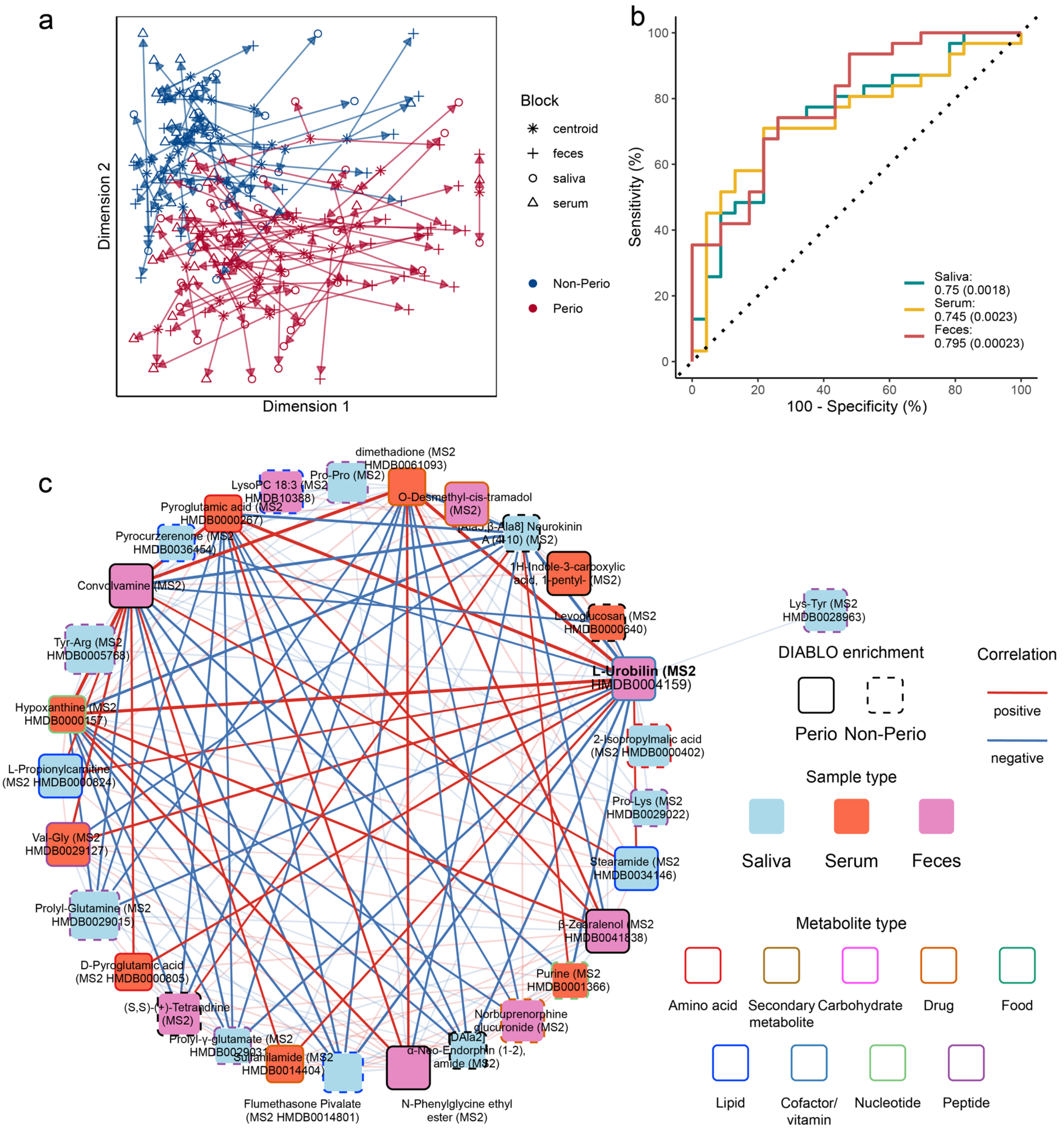

Figure S17. DIABLO integration of metabolomes of saliva, serum, and feces.

(A**)** Arrow plot of metabolome from saliva, serum, and feces of the final DIABLO model for discriminating periodontitis. (B**)** Receiver operating characteristic (ROC) curves of selection of metabolic features in saliva, serum, and feces for classification of periodontitis. **c** Inter-sample correlation network of MS2-annotated metabolic features among saliva, serum, and feces, with a correlation coefficient threshold of (r = 0.5). The highlighted edges were with absolute correlation coefficients |r| larger than 0.6. When no HMDB ID was presented, the feature was annotated by an in-house library only. L-urobilin in feces was highlighted in bold.

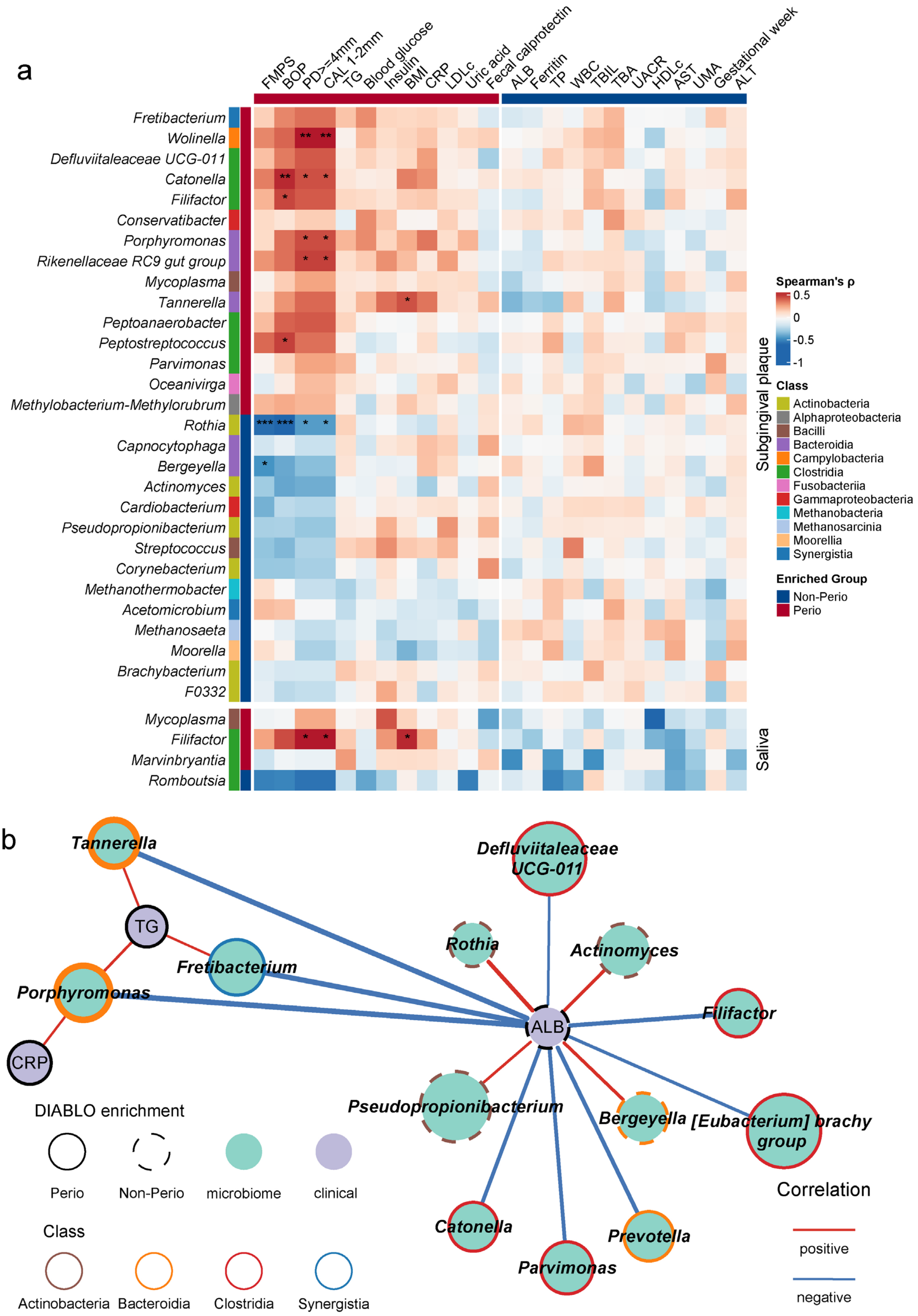

Figure S18. Relationship between microbiomic genera and clinical numeric determinants.

(A) DESeq2-enriched genera (relative abundance) in subgingival plaques and saliva with sPLS-DA-

selected clinical numeric determinants (|loading| > 0.1) plus periodontal parameters were included for pairwise Spearman's correlation analysis with FDR (Benjamini-Hochberg) adjustment for *P* values. Enriched groups for microbiome were from DESeq2 results, while enriched groups for clinical determinants were from the sPLS-DA model. Correlation matrices were shown with asterisks representing the significance of FDR-adjusted *P* values: *, FDR < 0.05; **, FDR < 0.01, ***, FDR < 0.001. The exact adjusted *P* values (FDR) are provided in Table S28. (B**)** DIABLO-integrated correlation network (|r| > 0.3) between microbial genera in subgingival plaques and clinical numeric determinants. Nodes of 'tie' genera were not shown. The line width represented |*r*|. TG, triglyceride; CRP, C-reactive protein; ALB, serum albumin.

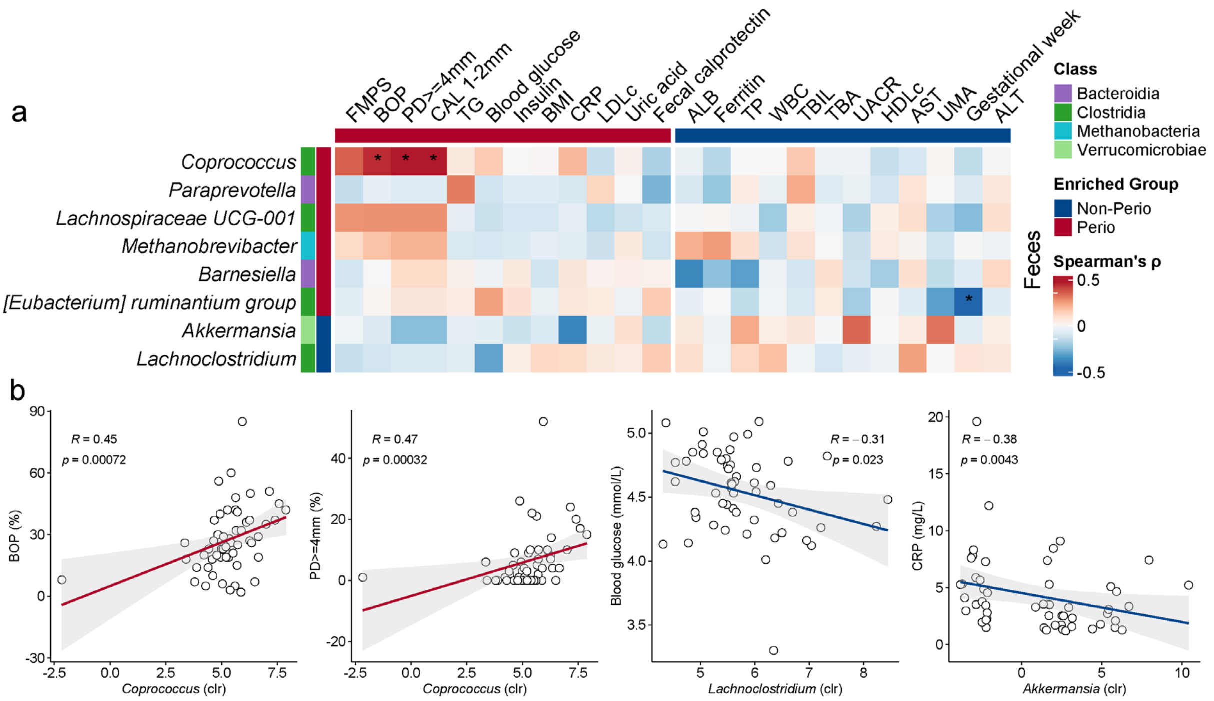

Figure S19. Relationship between fecal microbiomic genera and clinical numeric determinants. (A**)** DESeq2-enriched genera (clr) in feces with sPLS-DA-selected clinical numeric determinants (|loading| > 0.1) plus periodontal parameters were included for pairwise Spearman's correlation analysis with FDR (Benjamini-Hochberg) adjustment for *P* values. Enriched groups for microbiome were from DESeq2 results, while enriched groups for clinical determinants were from the sPLS-DA model. Correlation matrices were shown with asterisks representing the significance of FDR-adjusted *P* values: *, FDR < 0.05. The exact adjusted *P* values (FDR) are provided in Table S29. (B**)** Scatter plots of Spearman's correlation between selected fecal genera and clinical parameters.

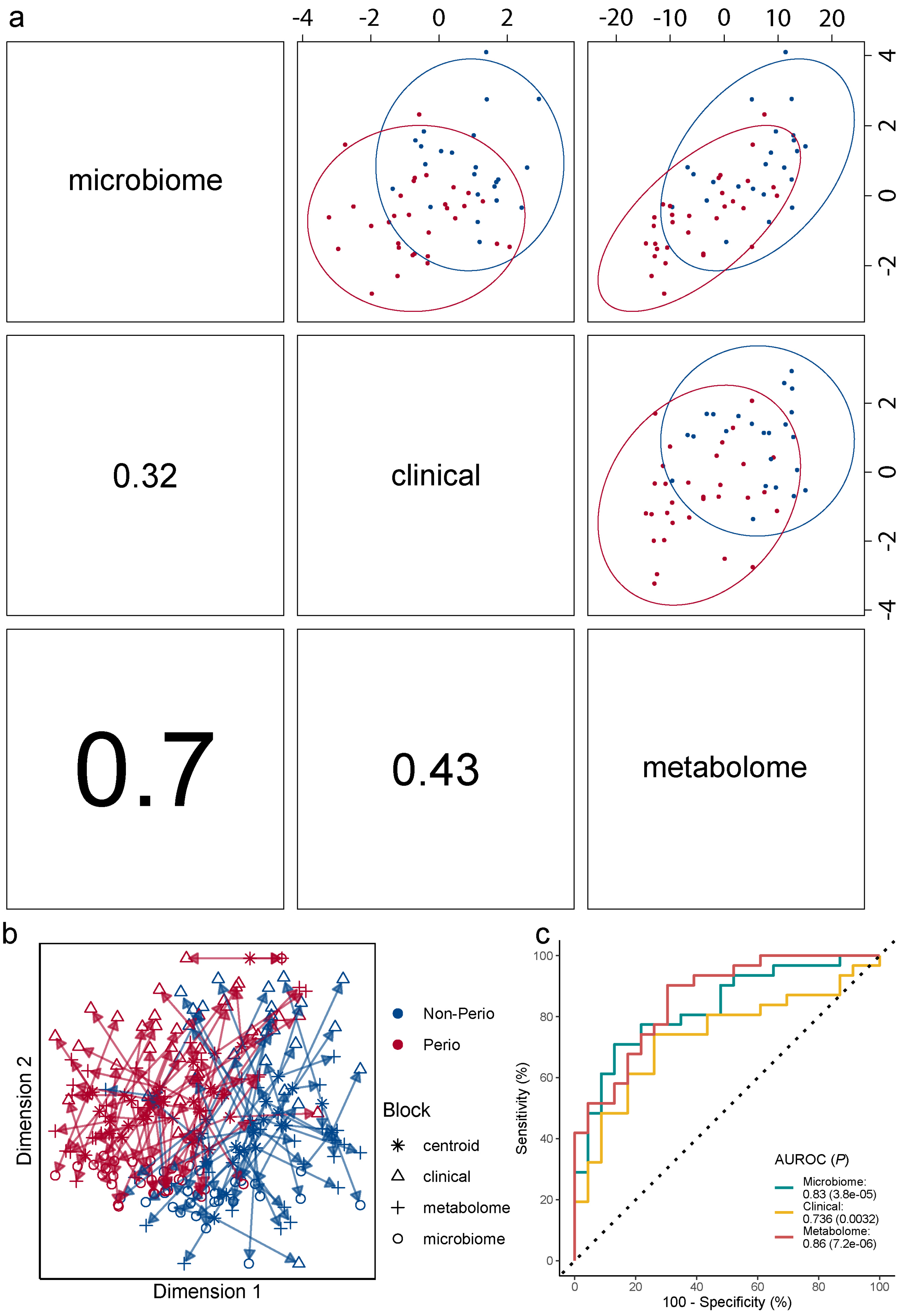
a
microbiome
0.32
clinical
0.7
0.43
metabolome
-4
-2
0
2
-20
-10
0
10
20
4
2
0
-2
2
0
-2
-4
b
Dimension 2
Dimension 1
Non-Perio
Perio
Block
centroid
clinical
metabolome
microbiome
c
Sensitivity (%)
100 - Specificity (%)
100
80
60
40
20
0
0
20
40
60
80
100
AUROC (P)
Microbiome:
0.83 (3.8e-05)
Clinical:
0.736 (0.0032)
Metabolome:
0.86 (7.2e-06)

Figure S20. Integration of fecal microbiota, metabolites, and systemic clinical determinants between Perio (n = 31) and Non-Perio (n = 23) groups using DIABLO.

(A**)** DIABLO plot of Component 1 of the integration of fecal microbiome, metabolome, and clinical numeric determinants with multiblock sPLS-DA plots and correlation coefficients shown. 95% confidence ellipses were shown in multiblock sPLS-DA plots. (B**)** Arrow plot of the final DIABLO model for discriminating periodontitis. (C**)** ROC curves of selection of fecal microbiomic genera, metabolic features, and clinical determinants in for classification of periodontitis by the final DIABLO model. AUROC and Wilcoxon test *P* values are shown.

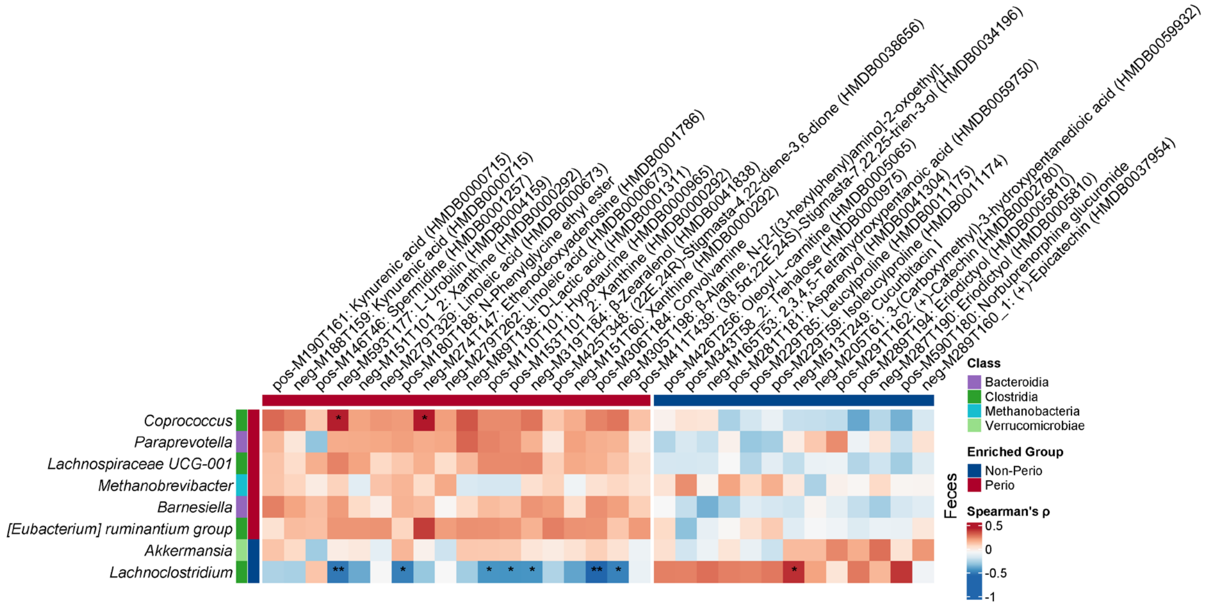

Figure S21. Spearman's correlation between microbiomic genera and metabolic features in feces. Pairwise Spearman's correlation analysis between DESeq2-enriched genera (clr) in feces and Venn-selected MS2-annotated metabolic features with FDR (Benjamini-Hochberg) adjustment for P values. Correlation matrices were shown with asterisks representing the significance of FDR-adjusted P values: *, FDR < 0.05; **, FDR < 0.01. The exact adjusted P values (FDR) are provided in Table S31.

Supplementary tables

Table S1. The demographic, periodontal and clinical datasets of the 54 subjects

Presented as a separate Excel file.

Abbreviations of table headers:

FMPS: full-mouth plaque score; SUP: suppuration; BOP: bleeding on probing; PD: probing depth; CAL: clinical attachment loss; M: tooth mobility; FI: furcation involvement; AAP/EFP(CAL): AAP/EFP 2018 classification based on CAL; GA: gestational age; Biochemical: biochemical pregnancy; Missed: missed abortion; Induced: induced abortion; Other: other abortion; NCDs: noncommunicable diseases; Medicine: use of immunosuppressants, bisphosphosphonate medications for osteoporosis treatment or steroids and other hormone- and immune-related drugs; Periodontal Tx: periodontal treatment; BOB: bleeding on brushing; BMI: body mass index at first trimester; SBP: systolic blood pressure; DBP: diastolic blood pressure; WBC: white blood cell; CRP: C-reactive protein; UMA: urine microalbumin; UCr: urinary creatinine; UACR: urinary albumin/creatinine ratio; TBIL: total bilirubin; TBA: total bile acid; ALT: alanine aminotransferase; AST: aspartate transaminase; TP: total protein; TC: total cholesterol; TG: triglyceride; HDLc: high-density lipoprotein cholesterol; LDLc: low-density lipoprotein cholesterol; HbA1c: glycosylated hemoglobin; TSH: thyroid-stimulating hormone; T3: triiodothyronine; T4: thyroxine; Tg: thyroglobulin; hCG: human chorionic gonadotropin; GDM: gestational diabetes mellitus; H. pylori: *Helicobacter pylori*; ECG: electrocardiogram; PCOS: polycystic ovary syndrome; ROH: region of homozygosity; HSV: herpes simplex virus; HPV: human papillomavirus; LSIL: low-grade squamous intraepithelial lesion.

Disease Group: Non-Perio (Healthy/Gingivitis) and Perio (Periodontitis), based on AAP/EFP(CAL).

Biochemical parameters are obtained from the first-time obstetric registration records of pregnant women at SMCHH.

Table S2. Demographic characteristics of the 54 subjects via the questionnaires

Characteristics	Total (N=54)	Healthy & gingivitis (N=23)	Periodontitis (N=31)	p-Value
**Age (yrs)**	29.7±2.6	29.4±2.3	29.8±2.9	0.543
**Educational level**				0.826
Primary and middle school	4 (7.4)	1 (4.3)	3 (9.7)	
High school	15 (27.8)	7 (30.4)	8 (25.8)	
University level or higher	35 (64.8)	15 (65.2)	20 (64.5)	
**Household monthly income (RMB)**				0.620
<9,000	11 (20.4)	6 (26.1)	5 (16.1)	
9,000 to 19,999	15 (27.8)	5 (21.7)	10 (32.3)	
≥20,000	28 (51.9)	12 (52.2)	16 (51.6)	
**Smoking**				NA
No	54 (100.0)	23 (100.0)	31 (100.0)	
Yes	0 (0.0)	0 (0.0)	0 (0.0)	
**Passive smoking**				1.00
No	47 (87.0)	20 (87.0)	27 (87.1)	
Yes	7 (13.0)	3 (13.0)	4 (12.9)	
**Alcohol consumption**				1.00
No	52 (96.3)	22 (95.7)	29 (96.8)	
Yes	2 (3.7)	1 (4.3)	1 (3.2)	
**Dietary type**				NA
Chinese (balanced)	54 (100.0)	23 (100.0)	31 (100.0)	
**Salting food (within 3 mo.)**				0.784
Never	20 (37.0)	9 (39.1)	11 (35.5)	
Sometimes	34 (63.0)	14 (60.9)	20 (64.5)	
**Smoking food (within 3 mo.)**				0.697
Never	36 (66.7)	16 (69.6)	20 (64.5)	
Sometimes	18 (33.3)	7 (30.4)	11 (35.5)	
**Vegetables & fruits (within 3 mo.)**				0.675
Never	1 (1.9)	1 (4.3)	0 (0.0)	
Sometimes	1 (1.9)	0 (0.0)	1 (3.2)	

Almost everyday	52 (96.3)	22 (95.7)	30 (96.8)	
**Grains & beans (within 3 mo.)**				0.192
Never	4 (7.4)	1 (4.3)	3 (9.7)	
Sometimes	25 (46.3)	14 (60.9)	11 (35.5)	
Almost everyday	25 (46.3)	8 (34.8)	17 (54.8)	
**High carbohydrate & fat food (within 3 mo.)**				0.903
Never	21 (38.9)	10 (43.5)	11 (35.5)	
Sometimes	30 (55.6)	12 (52.2)	18 (58.1)	
Almost everyday	3 (5.6)	1 (4.3)	2 (6.5)	
**Probiotics (within 3 mo.)**				NA
No	54 (100.0)	23 (100.0)	31 (100.0)	
Yes	0 (0.0)	0 (0.0)	0 (0.0)	
**Yogurt (within 3 mo.)**				0.730
≤ Once per week	20 (37.0)	10 (43.5)	10 (32.3)	
1−3 times per week	22 (40.7)	8 (34.8)	14 (45.2)	
4−6 times per week	4 (7.4)	1 (4.3)	3 (9.7)	
Everyday	8 (14.8)	4 (17.4)	4 (12.9)	
**Fertilization**				0.623
Natural	38 (70.4)	17 (73.9)	21 (67.7)	
Assisted	16 (29.6)	6 (26.1)	10 (32.3)	
**History of parity**				0.981
0	40 (74.1)	17 (73.9)	23 (74.2)	
1	14 (25.9)	6 (26.1)	8 (25.8)	
**History of abortion**				0.684
No	33 (61.1)	13 (56.5)	20 (64.5)	
1	12 (22.2)	5 (21.7)	7 (22.6)	
≥2	9 (16.7)	5 (21.7)	4 (12.9)	

Presented as Number (%) or Mean ± SD or Median (IQR). Pearson's χ^2 test or Student's *t*-test.

Abbreviations: SD, standard difference; IQR, interquartile range.

Table S3. Other demographic characteristics of the 54 subjects via the questionnaires

Characteristics	Total (N=54)	Healthy & gingivitis (N=23)	Periodontitis (N=31)
**Biochemical abortion***			
No	44 (81.5)	19 (82.6)	25 (80.6)
Yes	4 (7.4)	1 (4.3)	3 (9.7)
**Missed abortion***			
No	47 (87.0)	19 (82.6)	28 (90.3)
Yes	1 (1.9)	1 (4.3)	0 (0.0)
**Stillbirth***			
No	44 (81.5)	18 (78.3)	26 (83.9)
Yes	4 (7.4)	2 (8.7)	2 (6.5)
**Chromosomal defects***			
No	48 (88.9)	20 (87.0)	28 (90.3)
Yes	0 (0.0)	0 (0.0)	0 (0.0)
**Induced abortion***			
No	46 (85.2)	20 (87.0)	26 (83.9)
Yes	2 (3.7)	0 (0.0)	2 (6.5)
**Other abortion***			
No	46 (85.2)	18 (78.3)	28 (90.3)
Yes	2 (3.7)	2 (8.7)	0 (0.0)
**Regular menstruation**			
No	12 (22.2)	7 (30.4)	5 (16.1)
Yes	42 (77.8)	16 (69.6)	26 (83.9)
**NCDs**			
No	52 (96.3)	21 (91.3)	31 (100.0)
Other	2 (3.7)	2 (8.7)	0 (0.0)
**Genetic disorders**			
No	54 (100.0)	23 (100.0)	31 (100.0)
Yes	0 (0.0)	0 (0.0)	0 (0.0)
**Congenital disorders**			
No	54 (100.0)	23(100.0)	31 (100.0)

Yes	0 (0.0)	0 (0.0)	0 (0.0)
**Pulmonary disease**			
No	54 (100.0)	23 (100.0)	31 (100.0)
Yes	0 (0.0)	0 (0.0)	0 (0.0)
**Kidney disease**			
No	54 (100.0)	23 (100.0)	31 (100.0)
Yes	0 (0.0)	0 (0.0)	0 (0.0)
**Gastric disease**			
No	52 (96.3)	22 (95.7)	30 (96.8)
Yes	2 (3.7)	1 (4.3)	1 (3.2)
**Other disease**			
No	53 (98.1)	22 (95.7)	31 (100)
Yes	1 (1.9)	1 (4.3)	0 (0.0)
**Medicine**[§]			
No	50 (92.6)	20 (87.0)	30 (96.8)
Yes	4 (7.4)	3 (13.0)	1 (3.2)
**Antibiotics (within 3 mo.)**			
No	54 (100.0)	23 (100.0)	31 (100.0)
Yes	0 (0.0)	0 (0.0)	0 (0.0)
**Hazardous substance**			
No	52 (96.3)	23 (100.0)	29 (93.5)
Yes	2 (3.7)	0 (0.0)	2 (6.5)
**Periodontal Tx (in 12 mo.)**			
No	46(85.2)	20 (87.0)	26 (83.9)
Yes	8 (14.8)	3 (13.0)	5 (16.1)
**Regular Dental Visit**			
Never	32 (59.3)	12 (52.2)	20 (64.5)
Once per 2 yrs	6 (11.1)	5 (21.7)	1 (3.2)
Once per yr	14 (25.9)	5 (21.7)	9 (29.0)
Twice per yr	1 (1.9)	0 (0.0)	1 (3.2)
> Twice per yr	1 (1.9)	1 (4.3)	0 (0.0)
**Periodontitis Family History**			

No	25 (46.3)	14 (60.9)	11 (35.5)
Yes	9 (16.7)	4 (17.4)	5 (16.1)
Unknown	20 (37.0)	5 (21.7)	15 (48.4)
**Toothbrushing (per day)**			
Once	2 (3.7)	1 (4.3)	1 (3.2)
Twice	48 (88.9)	20 (87.0)	28 (90.3)
> Twice	4 (7.4)	2 (8.7)	2 (6.5)
**Bleeding on brushing**			
No	27 (50.0)	17 (73.9)	10 (32.3)
Yes	27 (50.0)	6 (26.1)	21 (67.7)
**Dental flossing**			
Never	34 (63.0)	13 (56.5)	21 (67.7)
Floss	19 (35.2)	10 (43.5)	9 (29.0)
Interdental brush	1 (1.9)	0 (0.0)	1 (3.2)
**Other dental cleaning**			
No	42 (77.8)	18 (78.3)	24 (77.4)
Yes	12 (22.2)	5 (21.7)	7 (22.6)

Data are presented as Number (%) or Mean ± SD or Median (IQR).

Abbreviations: SD, standard difference; IQR, interquartile range.

*The number may not add up to the total sample size due to missing values.

§ Continuous usage of immunosuppressants, bisphosphosphonate medications for osteoporosis treatment or steroids and other hormone- and immune-related drugs.

Table S4. Summary of clinical parameters

A. Major clinical parameters (7−13 gestational weeks)

Characteristics	Total (N = 54)	Healthy/Gingivitis (N = 23)	Periodontitis (N = 31)	P value	Test	Missing (N)
FMPS, % of tooth sites	82.00(75.00–93.00)	79.00(64.00–90.00)	86.00(79.50–99.00)	**0.0118**	Wilcoxon rank sum	
BOP, % of tooth sites	26.00(18.25–37.00)	18.00(7.00–20.50)	32.00(25.50–42.00)	**<0.0001**	Wilcoxon rank sum	
PD>=4mm, % of tooth sites	3.50(0.00–9.00)	0.00(0.00–0.00)	8.00(4.00–14.50)	**<0.0001**	Wilcoxon rank sum	
CAL 1-2mm, % of tooth sites	3.50(0.00–9.00)	0.00(0.00–0.00)	8.00(4.00–14.50)	**<0.0001**	Wilcoxon rank sum	
Gestational week at exam	10.85(8.93–12.00)	12.00(8.95–12.00)	10.20(8.95–12.00)	0.4582	Wilcoxon rank sum	
Menarche age (yr)	13.00(12.00–14.00)	13.00(13.00–14.00)	13.00(12.00–13.50)	0.0690	Wilcoxon rank sum	
BMI, kg/m^2	20.70(19.44–23.06)	20.42(19.17–21.79)	21.16(20.08–23.43)	0.1487	Wilcoxon rank sum	1
SBP, mmHg	110.49±10.23	108.59±9.32	111.84±10.77	0.2473	Student's t	1
DBP, mmHg	65.04±6.07	65.23±7.39	64.90±5.06	0.8596	Student's t	1
WBC, $\times 10^9$ cells/L	8.97±2.76	9.48±3.38	8.59±2.18	0.2772	Student's t	
CRP, mg/L	3.40(2.12–5.29)	3.09(2.21–5.15)	3.55(2.03–5.78)	0.3360	Wilcoxon rank sum	
UMA, mg/L	11.05(6.89–16.21)	11.17(9.41–16.05)	10.67(6.14–16.77)	0.5816	Wilcoxon rank sum	
UCr, mmol/L	21.62±8.46	21.69±8.19	21.57±8.79	0.9568	Student's t	
UACR, mg/mmol	0.57(0.36–0.77)	0.58(0.39–0.85)	0.55(0.32–0.72)	0.5230	Wilcoxon rank sum	
Uric acid, µmol/L	227.00(208.00–255.25)	224.00(203.50–245.50)	233.00(219.50–264.50)	0.1616	Wilcoxon rank sum	
TBIL, µmol/L	10.00(7.20–12.40)	10.70(7.30–13.10)	9.70(7.15–12.25)	0.4260	Wilcoxon rank sum	
TBA, µmol/L	1.50(1.10–2.60)	1.60(1.05–2.60)	1.50(1.20–2.68)	0.7058	Wilcoxon rank sum	1
ALT, U/L	15.00(11.00–22.75)	15.00(11.50–23.50)	14.00(11.00–21.00)	0.6357	Wilcoxon rank sum	
AST, U/L	18.00(16.00–21.00)	18.00(16.00–21.50)	17.00(16.00–21.00)	0.4987	Wilcoxon rank sum	
TP, g/L	68.83±3.83	69.60±3.49	68.25±4.02	0.1939	Student's t	
ALB, g/L	37.97±2.63	38.83±2.46	37.34±2.60	**0.0357**	Student's t	
TC, mmol/L	4.47±0.62	4.42±0.72	4.51±0.55	0.6241	Student's t	1
TG, mmol/L	1.15(0.88–1.33)	1.01(0.84–1.29)	1.28(0.95–1.47)	0.0710	Wilcoxon rank sum	1

HDLc, mmol/L	1.69±0.33	1.75±0.39	1.65±0.28	0.3118	Student's t	1
LDLc, mmol/L	2.18±0.47	2.09±0.48	2.25±0.46	0.2403	Student's t	1
Ferritin, mg/L	55.80(27.75–90.47)	65.30(48.05–108.50)	41.00(25.10–89.00)	0.1216	Wilcoxon rank sum	
Insulin, pmol/L	29.25(18.60–42.77)	23.21(18.61–36.23)	36.72(20.77–43.51)	0.1075	Wilcoxon rank sum	
Blood glucose, mmol/L	4.58(4.33–4.78)	4.48(4.25–4.62)	4.73(4.36–4.85)	**0.0381**	Wilcoxon rank sum	
HbA1c, %	5.10±0.23	5.06±0.25	5.13±0.21	0.3012	Student's t	
TSH, mIU/L	1.30±0.82	1.31±0.63	1.29±0.96	0.9286	Student's t	1
FT4, pmol/L	11.25(10.63–12.82)	11.81(10.71–12.86)	11.16(10.50–12.70)	0.5299	Wilcoxon rank sum	1
Thyroglobulin, ng/ml	0.60(0.40–1.92)	0.60(0.40–1.17)	0.55(0.40–2.02)	0.9296	Wilcoxon rank sum	4
Fecal calprotectin, ng/ml	359.72(120.96–1148.00)	371.84(159.70–707.09)	347.59(89.80–1720.33)	0.7259	Wilcoxon rank sum	4

The normality was tested using Shapiro-Wilk's test, to decide the choice of Student's t or Wicoxon rank sum test.

Data are presented as mean±SD or median (IQR). Boldface is used to show the significance when P<0.05.

Note: SD, standard difference; IQR, interquartile range; BMI, body mass index; SBP, systolic blood pressure; DBP, diastolic blood pressure; WBC, white blood cell; CRP, C-reactive protein; UMA, urine microalbumin; UCr, urinary creatinine; UACR, urinary albumin/creatinine ratio; TBIL, total bilirubin; TBA, total bile acid; ALT, alanine aminotransferase; AST, aspartate transaminase; TP, total protein; ALB, serum albumin; TC, total cholesterol; TG, triglyceride; HDL-c, high-density lipoprotein cholesterol; LDL-c, low-density lipoprotein cholesterol; HbA1c, hemoglobin A1c ; TSH, thyroid-stimulating hormone; FT4, free thyroxine.

Healthy/Gingivitis: Non-Perio; Periodontitis: Perio

B. Other clinical parameters

Other Characteristics	Total (N = 54)	Healthy/Gingivitis (N = 23)	Periodontitis (N = 31)	P value	Test	Missing (N)
Menstrual duration, d	6.00(5.00–7.00)	6.00(5.00–7.00)	6.00(5.00–7.00)	0.4744	Wilcoxon rank sum	12
Menstrual cycle, d	30.00(30.00–30.00)	30.00(30.00–30.00)	30.00(29.00–30.00)	0.6343	Wilcoxon rank sum	12
Height, cm	157.56±5.30	157.20±4.85	157.84±5.67	0.6559	Student's t	
Weight at exam, kg	51.75(47.85–58.17)	49.30(47.10–55.00)	52.50(49.50–60.00)	0.1710	Wilcoxon rank sum	
Waist Circumference, cm	75.40±9.27	74.20±10.05	76.50±8.56	0.4209	Student's t	10
Hip Circumference, cm	90.50±5.89	89.71±5.50	91.22±6.26	0.4013	Student's t	10
Waist-hip ratio (WHR)	0.83±0.07	0.83±0.08	0.84±0.07	0.5651	Student's t	10
Weight before pregnancy (kg)	52.70±7.30	51.41±7.56	53.66±7.07	0.2730	Student's t	
Weight (kg, 7-13 wk)	53.05±7.43	51.37±7.30	54.29±7.39	0.1538	Student's t	
Weight (kg, 34-36 wk)	64.62±7.48	62.39±7.14	66.08±7.47	0.1133	Student's t	11
Weight at delivery (kg)	64.93±7.86	61.58±6.96	67.08±7.77	**0.0241**	Student's t	13
TMPRSS2 (pg/ml)	3.70(3.01–4.61)	3.79(3.41–4.99)	3.48(2.87–4.46)	0.7977	Wilcoxon rank sum	
ACE2 (pg/ml)	48.06(40.88–58.24)	43.16(40.80–56.98)	49.64(43.14–57.99)	0.8002	Wilcoxon rank sum	
MMP-8 (pg/ml)	29902.04(8288.17–54351.84)	17410.27(9591.77–46317.24)	37035.27(7840.50–57903.59)	0.2818	Wilcoxon rank sum	3
IL-1β (pg/ml)	240.49(79.65–529.47)	238.75(114.71–351.52)	242.22(72.22–732.68)	0.3273	Wilcoxon rank sum	2
Weight gain (kg, 7-13 wk)	0.00(0.00–0.22)	0.00(-0.40–0.15)	0.00(0.00–0.50)	0.0832	Wilcoxon rank sum	
Weight gain (kg, 34-36 wk)	11.74±3.50	10.58±3.25	12.49±3.52	0.0765	Student's t	11
Weight gain at delivery (kg)	13.50(10.50–15.70)	11.90(9.60–14.22)	14.00(11.00–15.80)	0.1418	Wilcoxon rank sum	13

BMI before pregnancy, kg/m^2	20.53(19.31–23.02)	20.13(19.23–21.98)	20.72(19.46–23.48)	0.3213	Wilcoxon rank sum	
BMI (34-36 wk), kg/m2	26.09(23.83–27.90)	24.92(23.40–26.36)	26.51(24.83–28.03)	**0.0431**	Wilcoxon rank sum	11
BMI at delivery, kg/m^2	26.44±3.34	25.13±3.02	27.28±3.32	**0.0397**	Student's t	13

Table S5 XCMS parameters

Item	Parameter
method	centWave
minfrac	0.5
snthr	6
ppm	30
peakwidth	5,25
bw2	5
mzwid	0.015
mzdiff	0.01
profStep.OBIWarp	0.1

Table S6 metaX parameters

MS1 identification

Item	Parameter
adduct ion	pos: [M+H]+,[M+Na]+,[M+K]+,[M+NH4]+<br>neg: [M-H]-,[M+NH4-2H]-,[M+2Cl]2-,[2M-3H]3-
ms1 mass tolerance	10 ppm

MS2 identification

Item	Parameter
ms1 mass tolerance	0.01 Da
ms2 mass tolerance	0.05 Da
identification score cut off	75%
database	in-house,Massbank,HMDB,Lipidblast

Quantification of metabolites

Item	Parameter
missing value imputation	knn
scaling	pareto
normalization	pqn

Table S7. Summary of relative abundance of phyla, families and genera among subgingival plaques, saliva, and feces in both Non-Perio and Perio groups

Phylum	Sample	Rel. Abundance	Group
Bacteroidota	MSA.NPD	0.680449579	Saliva
Fusobacteriota	MSA.NPD	0.128228855	Saliva
Firmicutes	MSA.NPD	0.070167735	Saliva
Patescibacteria	MSA.NPD	0.052263017	Saliva
Actinobacteriota	MSA.NPD	0.044801259	Saliva
Campylobacterota	MSA.NPD	0.01333432	Saliva
Proteobacteria	MSA.NPD	0.003815047	Saliva
Unknown	MSA.NPD	0.003644508	Saliva
Cyanobacteria	MSA.NPD	0.001536847	Saliva
Verrucomicrobiota	MSA.NPD	0.000950895	Saliva
Synergistota	MSA.NPD	0.000558649	Saliva
Other	MSA.NPD	0.000191209	Saliva
Acidobacteriota	MSA.NPD	5.81E-05	Saliva
Bacteroidota	MSA.PD	0.674497057	Saliva
Fusobacteriota	MSA.PD	0.137684858	Saliva
Firmicutes	MSA.PD	0.073872469	Saliva
Actinobacteriota	MSA.PD	0.051435648	Saliva
Patescibacteria	MSA.PD	0.024813443	Saliva
Campylobacterota	MSA.PD	0.021922889	Saliva
Unknown	MSA.PD	0.006946403	Saliva
Proteobacteria	MSA.PD	0.003496436	Saliva
Cyanobacteria	MSA.PD	0.003457934	Saliva
Synergistota	MSA.PD	0.001310122	Saliva
Verrucomicrobiota	MSA.PD	0.000337983	Saliva
Other	MSA.PD	0.000127332	Saliva
Acidobacteriota	MSA.PD	9.74E-05	Saliva
Firmicutes	MST.NPD	0.514798879	Feces
Bacteroidota	MST.NPD	0.397989443	Feces
Actinobacteriota	MST.NPD	0.047606098	Feces
Verrucomicrobiota	MST.NPD	0.025868658	Feces
Proteobacteria	MST.NPD	0.007178069	Feces
Fusobacteriota	MST.NPD	0.003598606	Feces
Cyanobacteria	MST.NPD	0.001125344	Feces
Acidobacteriota	MST.NPD	0.000752208	Feces
Other	MST.NPD	0.000444269	Feces
Unknown	MST.NPD	0.000219035	Feces
Patescibacteria	MST.NPD	0.000215221	Feces
Campylobacterota	MST.NPD	0.000155335	Feces
Synergistota	MST.NPD	4.88E-05	Feces
Firmicutes	MST.PD	0.523292761	Feces
Bacteroidota	MST.PD	0.423008844	Feces
Actinobacteriota	MST.PD	0.032017222	Feces

Proteobacteria	MST.PD	0.013490012	Feces
Fusobacteriota	MST.PD	0.00204193	Feces
Acidobacteriota	MST.PD	0.001741643	Feces
Verrucomicrobiota	MST.PD	0.00143979	Feces
Cyanobacteria	MST.PD	0.001384085	Feces
Other	MST.PD	0.001171883	Feces
Patescibacteria	MST.PD	0.00015578	Feces
Unknown	MST.PD	0.000130476	Feces
Campylobacterota	MST.PD	7.29E-05	Feces
Synergistota	MST.PD	5.26E-05	Feces
Actinobacteriota	SBP.NPD	0.399631849	Subgingival plaque
Bacteroidota	SBP.NPD	0.279404367	Subgingival plaque
Fusobacteriota	SBP.NPD	0.14540796	Subgingival plaque
Firmicutes	SBP.NPD	0.044577152	Subgingival plaque
Campylobacterota	SBP.NPD	0.036710556	Subgingival plaque
Patescibacteria	SBP.NPD	0.03303813	Subgingival plaque
Other	SBP.NPD	0.027508181	Subgingival plaque
Proteobacteria	SBP.NPD	0.016057318	Subgingival plaque
Cyanobacteria	SBP.NPD	0.009996356	Subgingival plaque
Synergistota	SBP.NPD	0.003732613	Subgingival plaque
Acidobacteriota	SBP.NPD	0.002199788	Subgingival plaque
Unknown	SBP.NPD	0.001464131	Subgingival plaque
Verrucomicrobiota	SBP.NPD	0.000271599	Subgingival plaque
Bacteroidota	SBP.PD	0.427432612	Subgingival plaque
Actinobacteriota	SBP.PD	0.228984948	Subgingival plaque
Fusobacteriota	SBP.PD	0.188472967	Subgingival plaque
Firmicutes	SBP.PD	0.070559329	Subgingival plaque
Campylobacterota	SBP.PD	0.039282984	Subgingival plaque
Patescibacteria	SBP.PD	0.018034021	Subgingival plaque
Proteobacteria	SBP.PD	0.015580049	Subgingival plaque
Synergistota	SBP.PD	0.004922699	Subgingival plaque
Unknown	SBP.PD	0.003522951	Subgingival plaque
Cyanobacteria	SBP.PD	0.001415681	Subgingival plaque
Verrucomicrobiota	SBP.PD	0.000697181	Subgingival plaque
Other	SBP.PD	0.00063245	Subgingival plaque
Acidobacteriota	SBP.PD	0.000462128	Subgingival plaque

Family	Sample	Rel. Abundance	Group
Prevotellaceae	MSA.NPD	0.549664755	Saliva
Fusobacteriaceae	MSA.NPD	0.072795599	Saliva
Leptotrichiaceae	MSA.NPD	0.055433256	Saliva
Porphyromonadaceae	MSA.NPD	0.051415361	Saliva
Unknown	MSA.NPD	0.048565639	Saliva
Muribaculaceae	MSA.NPD	0.032987146	Saliva
Other	MSA.NPD	0.030660574	Saliva

Lachnospiraceae	MSA.NPD	0.027243779	Saliva
Weeksellaceae	MSA.NPD	0.026416817	Saliva
Micrococcaceae	MSA.NPD	0.019850428	Saliva
Saccharimonadaceae	MSA.NPD	0.017228411	Saliva
Actinomycetaceae	MSA.NPD	0.015022981	Saliva
Campylobacteraceae	MSA.NPD	0.012901074	Saliva
Bacteroidaceae	MSA.NPD	0.009027925	Saliva
Corynebacteriaceae	MSA.NPD	0.006720954	Saliva
Ruminococcaceae	MSA.NPD	0.005355329	Saliva
Flavobacteriaceae	MSA.NPD	0.005146531	Saliva
Peptostreptococcaceae	MSA.NPD	0.004455167	Saliva
Oscillospiraceae	MSA.NPD	0.003429573	Saliva
Tannerellaceae	MSA.NPD	0.002506357	Saliva
Rikenellaceae	MSA.NPD	0.001938116	Saliva
[Eubacterium] coprostanoligenes group	MSA.NPD	0.000814856	Saliva
Bifidobacteriaceae	MSA.NPD	0.000419373	Saliva
Prevotellaceae	MSA.PD	0.548704377	Saliva
Fusobacteriaceae	MSA.PD	0.073503781	Saliva
Leptotrichiaceae	MSA.PD	0.064181077	Saliva
Porphyromonadaceae	MSA.PD	0.058868032	Saliva
Weeksellaceae	MSA.PD	0.037674915	Saliva
Other	MSA.PD	0.037360214	Saliva
Unknown	MSA.PD	0.030792323	Saliva
Micrococcaceae	MSA.PD	0.024919531	Saliva
Lachnospiraceae	MSA.PD	0.024532681	Saliva
Campylobacteraceae	MSA.PD	0.021778618	Saliva
Actinomycetaceae	MSA.PD	0.01928926	Saliva
Saccharimonadaceae	MSA.PD	0.013070092	Saliva
Muribaculaceae	MSA.PD	0.011807258	Saliva
Bacteroidaceae	MSA.PD	0.008918165	Saliva
Peptostreptococcaceae	MSA.PD	0.007849784	Saliva
Corynebacteriaceae	MSA.PD	0.003890721	Saliva
Flavobacteriaceae	MSA.PD	0.002958338	Saliva
Tannerellaceae	MSA.PD	0.00256411	Saliva
Oscillospiraceae	MSA.PD	0.002352885	Saliva
Ruminococcaceae	MSA.PD	0.002188812	Saliva
Rikenellaceae	MSA.PD	0.001470871	Saliva
[Eubacterium] coprostanoligenes group	MSA.PD	0.001040726	Saliva
Bifidobacteriaceae	MSA.PD	0.000283431	Saliva
Bacteroidaceae	MST.NPD	0.276500976	Feces
Ruminococcaceae	MST.NPD	0.225414516	Feces
Lachnospiraceae	MST.NPD	0.198380641	Feces
Other	MST.NPD	0.08261374	Feces
Prevotellaceae	MST.NPD	0.055530388	Feces
Bifidobacteriaceae	MST.NPD	0.033967618	Feces
Oscillospiraceae	MST.NPD	0.027491959	Feces

Rikenellaceae	MST.NPD	0.02679191	Feces
Tannerellaceae	MST.NPD	0.026158561	Feces
[Eubacterium] coprostanoligenes group	MST.NPD	0.013392069	Feces
Unknown	MST.NPD	0.012196664	Feces
Muribaculaceae	MST.NPD	0.009291328	Feces
Peptostreptococcaceae	MST.NPD	0.006647423	Feces
Fusobacteriaceae	MST.NPD	0.003147447	Feces
Micrococcaceae	MST.NPD	0.001028951	Feces
Leptotrichiaceae	MST.NPD	0.000451158	Feces
Corynebacteriaceae	MST.NPD	0.000341144	Feces
Actinomycetaceae	MST.NPD	0.000216069	Feces
Saccharimonadaceae	MST.NPD	0.000215221	Feces
Campylobacteraceae	MST.NPD	8.50E-05	Feces
Porphyromonadaceae	MST.NPD	6.82E-05	Feces
Flavobacteriaceae	MST.NPD	5.20E-05	Feces
Weeksellaceae	MST.NPD	1.70E-05	Feces
Bacteroidaceae	MST.PD	0.251204836	Feces
Ruminococcaceae	MST.PD	0.224480262	Feces
Lachnospiraceae	MST.PD	0.216286493	Feces
Prevotellaceae	MST.PD	0.108835716	Feces
Other	MST.PD	0.05501819	Feces
Rikenellaceae	MST.PD	0.034911015	Feces
Oscillospiraceae	MST.PD	0.02804973	Feces
Bifidobacteriaceae	MST.PD	0.02332105	Feces
Tannerellaceae	MST.PD	0.014942097	Feces
[Eubacterium] coprostanoligenes group	MST.PD	0.014664868	Feces
Unknown	MST.PD	0.012881446	Feces
Peptostreptococcaceae	MST.PD	0.006310848	Feces
Muribaculaceae	MST.PD	0.005652304	Feces
Fusobacteriaceae	MST.PD	0.001848428	Feces
Micrococcaceae	MST.PD	0.000526895	Feces
Corynebacteriaceae	MST.PD	0.000261963	Feces
Leptotrichiaceae	MST.PD	0.000193502	Feces
Porphyromonadaceae	MST.PD	0.000175074	Feces
Flavobacteriaceae	MST.PD	0.000123626	Feces
Saccharimonadaceae	MST.PD	0.000119691	Feces
Actinomycetaceae	MST.PD	8.52E-05	Feces
Campylobacteraceae	MST.PD	7.14E-05	Feces
Weeksellaceae	MST.PD	3.53E-05	Feces
Prevotellaceae	SBP.NPD	0.16669461	Subgingival plaque
Actinomycetaceae	SBP.NPD	0.156224792	Subgingival plaque
Micrococcaceae	SBP.NPD	0.138862367	Subgingival plaque
Other	SBP.NPD	0.089917022	Subgingival plaque
Corynebacteriaceae	SBP.NPD	0.088477756	Subgingival plaque
Fusobacteriaceae	SBP.NPD	0.081743994	Subgingival plaque
Leptotrichiaceae	SBP.NPD	0.063663966	Subgingival plaque

Porphyromonadaceae	SBP.NPD	0.051464828	Subgingival plaque
Campylobacteraceae	SBP.NPD	0.036225654	Subgingival plaque
Flavobacteriaceae	SBP.NPD	0.031285114	Subgingival plaque
Unknown	SBP.NPD	0.030797705	Subgingival plaque
Saccharimonadaceae	SBP.NPD	0.018639795	Subgingival plaque
Lachnospiraceae	SBP.NPD	0.014806188	Subgingival plaque
Muribaculaceae	SBP.NPD	0.007347562	Subgingival plaque
Peptostreptococcaceae	SBP.NPD	0.004773171	Subgingival plaque
Ruminococcaceae	SBP.NPD	0.004033856	Subgingival plaque
Weeksellaceae	SBP.NPD	0.003399192	Subgingival plaque
Bacteroidaceae	SBP.NPD	0.003351134	Subgingival plaque
Tannerellaceae	SBP.NPD	0.003026994	Subgingival plaque
Oscillospiraceae	SBP.NPD	0.002586328	Subgingival plaque
Rikenellaceae	SBP.NPD	0.001278328	Subgingival plaque
Bifidobacteriaceae	SBP.NPD	0.000720373	Subgingival plaque
[Eubacterium] coprostanoligenes group	SBP.NPD	0.000679271	Subgingival plaque
Prevotellaceae	SBP.PD	0.241017807	Subgingival plaque
Porphyromonadaceae	SBP.PD	0.131612836	Subgingival plaque
Fusobacteriaceae	SBP.PD	0.120466432	Subgingival plaque
Actinomycetaceae	SBP.PD	0.09730484	Subgingival plaque
Leptotrichiaceae	SBP.PD	0.068006535	Subgingival plaque
Other	SBP.PD	0.067326588	Subgingival plaque
Corynebacteriaceae	SBP.PD	0.064544039	Subgingival plaque
Micrococcaceae	SBP.PD	0.056421883	Subgingival plaque
Campylobacteraceae	SBP.PD	0.037536254	Subgingival plaque
Lachnospiraceae	SBP.PD	0.024346454	Subgingival plaque
Unknown	SBP.PD	0.019338307	Subgingival plaque
Flavobacteriaceae	SBP.PD	0.017661222	Subgingival plaque
Peptostreptococcaceae	SBP.PD	0.009326456	Subgingival plaque
Saccharimonadaceae	SBP.PD	0.009193085	Subgingival plaque
Tannerellaceae	SBP.PD	0.008418736	Subgingival plaque
Muribaculaceae	SBP.PD	0.007505802	Subgingival plaque
Ruminococcaceae	SBP.PD	0.005084557	Subgingival plaque
Bacteroidaceae	SBP.PD	0.004543045	Subgingival plaque
Oscillospiraceae	SBP.PD	0.003742717	Subgingival plaque
Rikenellaceae	SBP.PD	0.003483352	Subgingival plaque
Weeksellaceae	SBP.PD	0.001971784	Subgingival plaque
[Eubacterium] coprostanoligenes group	SBP.PD	0.000807661	Subgingival plaque
Bifidobacteriaceae	SBP.PD	0.000339607	Subgingival plaque

Genus	Sample	Rel. Abundance	Group
Prevotella	MSA.NPD	0.430183126	Saliva
Alloprevotella	MSA.NPD	0.111655525	Saliva
Unknown	MSA.NPD	0.092600785	Saliva
Fusobacterium	MSA.NPD	0.07220981	Saliva
Other	MSA.NPD	0.065680543	Saliva

Leptotrichia	MSA.NPD	0.053935214	Saliva
Porphyromonas	MSA.NPD	0.051415361	Saliva
Bergeyella	MSA.NPD	0.026416817	Saliva
Rothia	MSA.NPD	0.019828263	Saliva
Actinomyces	MSA.NPD	0.014353803	Saliva
Campylobacter	MSA.NPD	0.012901074	Saliva
TM7x	MSA.NPD	0.011918663	Saliva
Bacteroides	MSA.NPD	0.009027925	Saliva
Corynebacterium	MSA.NPD	0.006720954	Saliva
Capnocytophaga	MSA.NPD	0.00513283	Saliva
Lachnospiraceae NK4A136 group	MSA.NPD	0.004780245	Saliva
Ruminococcus	MSA.NPD	0.001959231	Saliva
Blautia	MSA.NPD	0.001656466	Saliva
Faecalibacterium	MSA.NPD	0.001613485	Saliva
Alistipes	MSA.NPD	0.001486816	Saliva
Roseburia	MSA.NPD	0.001198807	Saliva
Parabacteroides	MSA.NPD	0.000795754	Saliva
Subdoligranulum	MSA.NPD	0.000669355	Saliva
Agathobacter	MSA.NPD	0.000614133	Saliva
F0332	MSA.NPD	0.000573084	Saliva
Bifidobacterium	MSA.NPD	0.000393332	Saliva
CAG-352	MSA.NPD	0.000174089	Saliva
UCG-002	MSA.NPD	0.000104512	Saliva
Prevotella	MSA.PD	0.461222494	Saliva
Alloprevotella	MSA.PD	0.082232365	Saliva
Other	MSA.PD	0.078945061	Saliva
Fusobacterium	MSA.PD	0.073493781	Saliva
Porphyromonas	MSA.PD	0.058868032	Saliva
Leptotrichia	MSA.PD	0.058455119	Saliva
Unknown	MSA.PD	0.050824521	Saliva
Bergeyella	MSA.PD	0.037674915	Saliva
Rothia	MSA.PD	0.024846568	Saliva
Campylobacter	MSA.PD	0.021778618	Saliva
Actinomyces	MSA.PD	0.018549091	Saliva
TM7x	MSA.PD	0.009057881	Saliva
Bacteroides	MSA.PD	0.008918165	Saliva
Corynebacterium	MSA.PD	0.003890721	Saliva
Capnocytophaga	MSA.PD	0.002922436	Saliva
Blautia	MSA.PD	0.002750688	Saliva
Ruminococcus	MSA.PD	0.001340071	Saliva
Lachnospiraceae NK4A136 group	MSA.PD	0.001198777	Saliva
Alistipes	MSA.PD	0.000778969	Saliva
F0332	MSA.PD	0.000585206	Saliva
Parabacteroides	MSA.PD	0.00056534	Saliva
Faecalibacterium	MSA.PD	0.00047479	Saliva
Roseburia	MSA.PD	0.000431889	Saliva
Bifidobacterium	MSA.PD	0.000113001	Saliva
Agathobacter	MSA.PD	4.39E-05	Saliva

Subdoligranulum	MSA.PD	3.76E-05	Saliva
UCG-002	MSA.PD	0	Saliva
CAG-352	MSA.PD	0	Saliva
Bacteroides	MST.NPD	0.276500976	Feces
Other	MST.NPD	0.187902415	Feces
Faecalibacterium	MST.NPD	0.163278582	Feces
Blautia	MST.NPD	0.057539092	Feces
Prevotella	MST.NPD	0.053869171	Feces
Unknown	MST.NPD	0.044274488	Feces
Bifidobacterium	MST.NPD	0.033967618	Feces
Alistipes	MST.NPD	0.02663245	Feces
Parabacteroides	MST.NPD	0.026113178	Feces
Subdoligranulum	MST.NPD	0.024095313	Feces
Lachnospiraceae NK4A136 group	MST.NPD	0.019617564	Feces
Roseburia	MST.NPD	0.019533345	Feces
Agathobacter	MST.NPD	0.018205498	Feces
UCG-002	MST.NPD	0.015984495	Feces
CAG-352	MST.NPD	0.01423628	Feces
Ruminococcus	MST.NPD	0.013326992	Feces
Fusobacterium	MST.NPD	0.001962918	Feces
Rothia	MST.NPD	0.001008538	Feces
Alloprevotella	MST.NPD	0.000591148	Feces
Leptotrichia	MST.NPD	0.000451158	Feces
Corynebacterium	MST.NPD	0.000341144	Feces
Actinomyces	MST.NPD	0.000166078	Feces
TM7x	MST.NPD	0.00013435	Feces
Campylobacter	MST.NPD	8.50E-05	Feces
Porphyromonas	MST.NPD	6.82E-05	Feces
Capnocytophaga	MST.NPD	5.20E-05	Feces
F0332	MST.NPD	5.00E-05	Feces
Bergeyella	MST.NPD	1.20E-05	Feces
Bacteroides	MST.PD	0.251204836	Feces
Other	MST.PD	0.159489373	Feces
Faecalibacterium	MST.PD	0.146439739	Feces
Prevotella	MST.PD	0.103364754	Feces
Blautia	MST.PD	0.049935249	Feces
Unknown	MST.PD	0.043058148	Feces
Subdoligranulum	MST.PD	0.037537451	Feces
Alistipes	MST.PD	0.034907747	Feces
Lachnospiraceae NK4A136 group	MST.PD	0.030179868	Feces
Agathobacter	MST.PD	0.028697516	Feces
Bifidobacterium	MST.PD	0.02332105	Feces
Roseburia	MST.PD	0.022706303	Feces
UCG-002	MST.PD	0.017438355	Feces
Ruminococcus	MST.PD	0.015798839	Feces
CAG-352	MST.PD	0.015657648	Feces
Parabacteroides	MST.PD	0.014912107	Feces
Alloprevotella	MST.PD	0.002110202	Feces

Fusobacterium	MST.PD	0.001755979	Feces
Rothia	MST.PD	0.000508255	Feces
Corynebacterium	MST.PD	0.000258997	Feces
Leptotrichia	MST.PD	0.000193502	Feces
Porphyromonas	MST.PD	0.000175074	Feces
Capnocytophaga	MST.PD	0.000123626	Feces
Campylobacter	MST.PD	7.14E-05	Feces
TM7x	MST.PD	5.49E-05	Feces
Actinomyces	MST.PD	4.91E-05	Feces
F0332	MST.PD	3.61E-05	Feces
Bergeyella	MST.PD	1.38E-05	Feces
Actinomyces	SBP.NPD	0.138772493	Subgingival plaque
Rothia	SBP.NPD	0.138150124	Subgingival plaque
Prevotella	SBP.NPD	0.117772767	Subgingival plaque
Other	SBP.NPD	0.108029804	Subgingival plaque
Corynebacterium	SBP.NPD	0.088464102	Subgingival plaque
Fusobacterium	SBP.NPD	0.081642218	Subgingival plaque
Leptotrichia	SBP.NPD	0.063153674	Subgingival plaque
Unknown	SBP.NPD	0.060565889	Subgingival plaque
Porphyromonas	SBP.NPD	0.051464828	Subgingival plaque
Alloprevotella	SBP.NPD	0.048429037	Subgingival plaque
Campylobacter	SBP.NPD	0.036225654	Subgingival plaque
Capnocytophaga	SBP.NPD	0.031285114	Subgingival plaque
F0332	SBP.NPD	0.015986589	Subgingival plaque
TM7x	SBP.NPD	0.004061414	Subgingival plaque
Bergeyella	SBP.NPD	0.003399192	Subgingival plaque
Bacteroides	SBP.NPD	0.003351134	Subgingival plaque
Faecalibacterium	SBP.NPD	0.002233329	Subgingival plaque
Lachnospiraceae NK4A136 group	SBP.NPD	0.001321437	Subgingival plaque
Blautia	SBP.NPD	0.000935013	Subgingival plaque
Alistipes	SBP.NPD	0.000744573	Subgingival plaque
Bifidobacterium	SBP.NPD	0.000701311	Subgingival plaque
UCG-002	SBP.NPD	0.000624992	Subgingival plaque
Ruminococcus	SBP.NPD	0.000580121	Subgingival plaque
Parabacteroides	SBP.NPD	0.000535754	Subgingival plaque
Roseburia	SBP.NPD	0.00050526	Subgingival plaque
Agathobacter	SBP.NPD	0.000372224	Subgingival plaque
Subdoligranulum	SBP.NPD	0.00034841	Subgingival plaque
CAG-352	SBP.NPD	0.00034354	Subgingival plaque
Prevotella	SBP.PD	0.193509229	Subgingival plaque
Porphyromonas	SBP.PD	0.131612836	Subgingival plaque
Fusobacterium	SBP.PD	0.120427618	Subgingival plaque
Other	SBP.PD	0.104577553	Subgingival plaque
Actinomyces	SBP.PD	0.083442655	Subgingival plaque
Leptotrichia	SBP.PD	0.066505376	Subgingival plaque
Corynebacterium	SBP.PD	0.064502103	Subgingival plaque
Rothia	SBP.PD	0.055565433	Subgingival plaque
Alloprevotella	SBP.PD	0.047075012	Subgingival plaque

Unknown	SBP.PD	0.042382629	Subgingival plaque
Campylobacter	SBP.PD	0.037536254	Subgingival plaque
Capnocytophaga	SBP.PD	0.017661222	Subgingival plaque
F0332	SBP.PD	0.013226238	Subgingival plaque
Bacteroides	SBP.PD	0.004543045	Subgingival plaque
Faecalibacterium	SBP.PD	0.00323773	Subgingival plaque
TM7x	SBP.PD	0.0029995	Subgingival plaque
Bergeyella	SBP.PD	0.001929448	Subgingival plaque
Lachnospiraceae NK4A136 group	SBP.PD	0.001878896	Subgingival plaque
Alistipes	SBP.PD	0.001516938	Subgingival plaque
Parabacteroides	SBP.PD	0.001382953	Subgingival plaque
Blautia	SBP.PD	0.001104804	Subgingival plaque
CAG-352	SBP.PD	0.00090776	Subgingival plaque
Roseburia	SBP.PD	0.000881153	Subgingival plaque
Ruminococcus	SBP.PD	0.000453949	Subgingival plaque
UCG-002	SBP.PD	0.000429494	Subgingival plaque
Bifidobacterium	SBP.PD	0.000296526	Subgingival plaque
Subdoligranulum	SBP.PD	0.000294109	Subgingival plaque
Agathobacter	SBP.PD	0.000119539	Subgingival plaque

Table S8. Summary of PERMANOVA (Bray-Curtis distances) of microbiome data

By sample types

	Df	SumOfSqs	R2	F	Pr(>F)	
Sample	2	19.422	0.29704	33.593	0.001	***
Residual	159	45.963	0.70296			
Total	161	65.385	1			

Signif. codes: 0 '***' 0.001 '**' 0.01 '*' 0.05 '.' 0.1 ' ' 1

By disease

Subgingival plaque

	Df	SumOfSqs	R2	F	Pr(>F)	
Disease	1	0.5841	0.03227	1.7342	0.004	**
Residual	52	17.5151	0.96773			
Total	53	18.0992	1			

Signif. codes: 0 '***' 0.001 '**' 0.01 '*' 0.05 '.' 0.1 ' ' 1

Saliva

	Df	SumOfSqs	R2	F	Pr(>F)
Disease	1	0.2597	0.01913	1.014	0.409
Residual	52	13.3178	0.98087		
Total	53	13.5775	1		

Feces

	Df	SumOfSqs	R2	F	Pr(>F)
Disease	1	0.2619	0.01833	0.9709	0.473
Residual	52	14.0246	0.98167		
Total	53	14.2865	1		

PERMANOVA with 999 permutations

Table S9. Summary of taxa (families, genera, species and ASVs) identified as differentially abundant between Perio and Non-Perio groups using DESeq2 with or without adjustment with BMI

Presented as a separate Excel file.

P values from Wald test (two-sided) implemented with DESeq2 with Benjamini-Hochberg adjustment for multiple comparisons. FDR cutoff: 0.2.

Table headers:

ef_logFC: log2 (Perio to Non-Perio Fold change); padj: FDR-adjusted P

Table S10. Summary of taxa (families, genera and species) identified as differentially abundant between Perio and Non-Perio groups using ANCOM-BC2 with or without adjustment with BMI
Presented as separate Excel files (A-C).

Table headers:

DiseasePD: Perio.

q values, Benjamini-Hochberg adjustment for multiple comparisons. q (FDR-adjusted P) cutoff: 0.2.

lfc: natural log(Perio to Non-Perio Fold change) as ANCOM-BC2 algorithm. Therefore |lfc| > 0.69 is |log2(Perio to Non-Perio Fold change)| > 1.

passed_ss: passed sensitivity analysis score (ss) for the pseudo-count addition to zero counts.

se: Standard Error.

W: Wilcoxon test statistics.

Table S11. Summary of taxa (genera, species and ASVs) identified as differential contributors between Perio and Non-Perio groups using sPLS-DA
Presented as a separate Excel file.

Table headers:

importance: loading coefficient weight of selected taxa.

Table S12. Intra- and inter-correlation of genera between subgingival plaque and feces samples using SECOM (Pearson1 mode)
Presented as a separate Excel file.

Table S13. Summary of PICRUSt2-predicted functions identified as differentially abundant between Perio and Non-Perio groups using ANCOM-BC2
Presented as a separate Excel file.

Table S14. KEGG enrichment of ANCOM-BC2-selected PICRUSt2-predicted KO terms

geneID: KO terms.

Feces

ID	Description	GeneRatio	BgRatio	pvalue	p.adjust	qvalue	geneID	Count
map00121	Secondary bile acid biosynthesis	6/124	15/13584	2.40E-09	1.80E-07	1.46E-07	K15873/K15870/K15871/K15872/K15868/K15869	6
map02020	Two-component system	20/124	499/13584	2.37E-08	8.90E-07	7.24E-07	K05966/K15739/K07710/K18345/K10914/K11692/K13927/K07650/K07770/K02106/K11691/K11618/K07719/K07678/K11616/K07642/K07654/K11614/K00245/K11688	20
map00440	Phosphonate and phosphinate metabolism	6/124	44/13584	2.72324E-06	6.80809E-05	5.54203E-05	K06162/K05780/K06163/K06164/K06166/K19670	6
map01053	Biosynthesis of siderophore group nonribosomal peptides	5/124	36/13584	1.75675E-05	0.00032939	0.000268135	K02363/K12239/K04783/K12240/K04784	5
map00040	Pentose and glucuronate interconversions	5/124	89/13584	0.001316205	0.01974308	0.01607156	K08323/K08322/K05351/K17818/K00880	5
map00640	Propanoate metabolism	5/124	97/13584	0.001928822	0.024110275	0.01962661	K13921/K13919/K13920/K01699/K09788	5
map00053	Ascorbate and aldarate metabolism	4/124	62/13584	0.002452576	0.026277595	0.021390884	K01706/K01630/K01708/K00880	4
map00010	Glycolysis / Gluconeogenesis	5/124	106/13584	0.002842422	0.026647702	0.021692165	K13979/K16306/K13953/K01792/K02791	5
map00650	Butanoate metabolism	5/124	114/13584	0.003887053	0.030259533	0.024632321	K18118/K18120/K01640/K01027/K00245	5
map00564	Glycerophospholipid metabolism	5/124	115/13584	0.004034604	0.030259533	0.024632321	K07029/K00980/K03735/K03736/K04019	5

Subgingival plaque

ID	Description	GeneRatio	BgRatio	pvalue	p.adjust	qvalue	geneID	Count
map01240	Biosynthesis of cofactors	48/437	375/13584	1.70E-16	2.83E-14	2.07E-14	K05979/K02169/K09698/K09789/K02190/K02226/K03394/K00226/K02188/K00949/K09680/K00610/K17828/K02823/K02230/K02231/K03517/K00767/K01665/K00097/K00278/K00798/K06897/K03150/K04032/K03473/K02189/K05934/K03147/K00595/K02227/K03151/K00652/K02232/K02304/K02224/K00768/K03399/K02191/K01077/K02233/K02372/K13950/K02303/K13421/K19222/K03153/K01556	48
map02020	Two-component system	48/437	499/13584	9.18E-12	7.66E-10	5.60E-10	K07717/K07665/K07710/K07777/K01034/K01035/K02584/K07787/K01051/K07652/K03740/K03367/K14188/K03739/K19081/K19082/K01991/K09474/K08641/K07783/K11692/K07719/K07704/K18344/K07667/K07813/K02398/K07646/	48

							K06282/K06281/K07668/K01910/K03412/K11636/K11635/K00575/K01646/K05964/K02556/K01643/K05966/K03092/K07718/K01077/K07720/K12340/K07636/K00692	
map00860	Porphyrin metabolism	24/437	139/13584	1.41E-11	7.84E-10	5.73E-10	K16651/K04720/K02190/K02226/K03394/K02188/K02230/K02231/K00798/K03405/K04032/K02189/K05934/K00595/K02227/K02232/K02304/K02224/K00768/K03399/K02191/K02233/K02303/K00510	24
map01230	Biosynthesis of amino acids	27/437	238/13584	1.26E-08	5.28E-07	3.86E-07	K11645/K09758/K11358/K09011/K15635/K00290/K01960/K04516/K00651/K05827/K05828/K05829/K05830/K05831/K02203/K00821/K13853/K06209/K02502/K01089/K04486/K01620/K00832/K15633/K01914/K00266/K00548	27
map00650	Butanoate metabolism	18/437	114/13584	2.24E-08	7.49E-07	5.48E-07	K18119/K18120/K01034/K01035/K14534/K18122/K01715/K00248/K01615/K00175/K00174/K03737/K17865/K01039/K01040/K07250/K18369/K03366	18
map00540	Lipopolysaccharide biosynthesis	13/437	62/13584	6.54E-08	1.82025E-06	1.33091E-06	K19353/K16363/K03270/K02850/K00912/K00748/K00677/K03269/K06041/K01627/K00979/K02527/K02536	13
map01200	Carbon metabolism	32/437	365/13584	2.72E-07	6.49289E-06	4.7474E-06	K11261/K01848/K01849/K00844/K11645/K18119/K18120/K14534/K18122/K15022/K01715/K00248/K15635/K00625/K01676/K00177/K00176/K00175/K00174/K03737/K01960/K01610/K01637/K17865/K02203/K07404/K05606/K01846/K01847/K15633/K00029/K00018	32
map00240	Pyrimidine metabolism	16/437	111/13584	4.94E-07	1.03145E-05	7.54168E-06	K09913/K08722/K00226/K00610/K01119/K17828/K02823/K01493/K00757/K08693/K00756/K09769/K03787/K00876/K13421/K09019	16
map01503	Cationic antimicrobial peptide (CAMP) resistance	11/437	54/13584	9.25E-07	1.68607E-05	1.2328E-05	K08589/K10012/K03740/K03367/K14188/K03739/K12962/K00677/K12340/K01448/ K03585	11
map02030	Bacterial chemotaxis	8/437	26/13584	1.01E-06	1.68607E-05	1.2328E-05	K03409/K03410/K03411/K03412/K00575/K02410/K02556/K02557	8
map00520	Amino sugar and nucleotide sugar metabolism	18/437	156/13584	2.72597E-06	4.13851E-05	3.02595E-05	K13016/K10012/K00844/K17716/K07106/K02377/K01711/K01787/K01639/K12373/K13020/K13018/K08678/K02793/K01209/K01709/K02474/K02564	18
map00330	Arginine and proline metabolism	14/437	107/13584	8.18286E-06	0.000109959	8.03989E-05	K10794/K02626/K10793/K11358/K01581/K12251/K13747/K00657/K18123/K00294/K00819/K03343/K01473/K01474	14
map02040	Flagellar assembly	10/437	55/13584	8.55971E-06	0.000109959	8.03989E-05	K02414/K02413/K02398/K02418/K02396/K02422/K02410/K02556/K03092/K02557	10
map00300	Lysine biosynthesis	9/437	48/13584	1.86867E-05	0.000222906	0.000162982	K05825/K00290/K05827/K05828/K05829/K05830/K05831/K15792/K00821	9
map00720	Carbon fixation pathways in prokaryotes	14/437	117/13584	2.30908E-05	0.000257077	0.000187967	K01848/K01849/K14534/K15022/K00625/K01676/K00177/K00176/K00175/K00174/K03737/K01960/K05606/K01847	14
map00470	D-Amino acid metabolism	9/437	55/13584	5.8046E-05	0.000605856	0.000442983	K17898/K17899/K01844/K18011/K01753/K10794/K10793/K03367/K14188	9

map00250	Alanine, aspartate and glutamate metabolism	10/437	70/13584	7.54894E-05	0.000741573	0.000542215	K09758/K11358/K00610/K00278/K00294/K00260/K01914/K00266/K15371/K07250	10
map00620	Pyruvate metabolism	14/437	133/13584	9.63041E-05	0.000893488	0.000653291	K02594/K00625/K01676/K00175/K03778/K00174/K03737/K01960/K01610/K13954/K01026/K01571/K01069/K00029	14
map00340	Histidine metabolism	8/437	47/13584	0.00011233	0.000987324	0.000721901	K00603/K01468/K01712/K01745/K17363/K02502/K01089/K04486	8
map00010	Glycolysis / Gluconeogenesis	12/437	106/13584	0.000150679	0.001258167	0.000919933	K00844/K11645/K15635/K04041/K00175/K00174/K03737/K01610/K13954/K15633/K01785/K01222	12
map00640	Propanoate metabolism	11/437	97/13584	0.000277867	0.002209703	0.001615667	K01848/K01849/K08325/K00248/K00625/K13921/K01026/K01734/K05606/K01847/K07250	11
map00220	Arginine biosynthesis	8/437	60/13584	0.000634697	0.004817924	0.003522718	K11358/K05828/K05829/K05830/K05831/K00821/K00260/K15371	8
map00260	Glycine, serine and threonine metabolism	11/437	109/13584	0.000759678	0.00551592	0.004033071	K01753/K15635/K02203/K01620/K17103/K15633/K00018/K00639/K00130/K00302/K00303	11
map00730	Thiamine metabolism	6/437	36/13584	0.000918922	0.006394166	0.004675217	K00949/K03150/K03147/K03151/K01077/K03153	6
map00310	Lysine degradation	10/437	98/13584	0.001198862	0.007935813	0.005802422	K01844/K18011/K18012/K18014/K01034/K01035/K18013/K01843/K00290/K07250	10
map01210	2-Oxocarboxylic acid metabolism	9/437	82/13584	0.001245506	0.007935813	0.005802422	K05825/K11358/K09011/K05827/K05828/K05829/K05830/K05831/K00821	9
map01250	Biosynthesis of nucleotide sugars	16/437	211/13584	0.001325678	0.007935813	0.005802422	K13016/K00844/K17716/K02377/K01711/K03270/K13020/K13018/K08678/K01709/K02474/K06041/K01627/K00979/K00067/K17947	16
map00020	Citrate cycle (TCA cycle)	8/437	67/13584	0.001330555	0.007935813	0.005802422	K01676/K00177/K00176/K00175/K00174/K03737/K01960/K01610	8
map00790	Folate biosynthesis	9/437	84/13584	0.001478896	0.008516402	0.006226931	K06920/K09457/K01665/K06897/K10026/K13818/K07141/K01077/K13950	9
map00630	Glyoxylate and dicarboxylate metabolism	10/437	104/13584	0.001884289	0.010489207	0.007669385	K01848/K01849/K01637/K18123/K00015/K17865/K05606/K01846/K01847/K00018	10
map01232	Nucleotide metabolism	11/437	123/13584	0.002044538	0.011014122	0.008053187	K09913/K01486/K08722/K03816/K01493/K00757/K08693/K01241/K00756/K03787/K00876	11
map00360	Phenylalanine metabolism	8/437	74/13584	0.002529921	0.013203026	0.009653647	K11358/K00832/K02609/K02610/K02611/K02612/K02618/K15866	8
map03430	Mismatch repair	6/437	44/13584	0.002682314	0.013574137	0.009924991	K07456/K03763/K03555/K03572/K07462/K06223	6
map00541	O-Antigen nucleotide sugar biosynthesis	9/437	99/13584	0.004563238	0.02241355	0.016388098	K13016/K17716/K02377/K01711/K13020/K13018/K01709/K02474/K00067	9
map00511	Other glycan degradation	4/437	22/13584	0.004876423	0.023267503	0.017012483	K01206/K15923/K12373/K01192	4

map00190	Oxidative phosphorylation	15/437	223/13584	0.005744401	0.026647639	0.019483934	K02120/K02117/K02124/K02121/K02118/K02123/K15986/K02122/K02119/K02274/K02275/K02276/K03889/K03890/K03891	15
map00910	Nitrogen metabolism	7/437	68/13584	0.006078276	0.02743438	0.020059175	K02586/K05601/K00260/K00266/K15371/K00362/K00363	7
map00270	Cysteine and methionine metabolism	10/437	124/13584	0.006692789	0.028835147	0.021083372	K09758/K01761/K12960/K08963/K11358/K00651/K00899/K00832/K00558/K00548	10
map00780	Biotin metabolism	4/437	24/13584	0.006733956	0.028835147	0.021083372	K02169/K09789/K00652/K02372	4
map00643	Styrene degradation	4/437	25/13584	0.007816591	0.031864147	0.023298084	K01026/K01039/K01040/K01555	4
map00040	Pentose and glucuronate interconversions	8/437	89/13584	0.007822934	0.031864147	0.023298084	K01804/K01051/K01812/K00041/K01815/K03077/K01685/K09988	8
map00552	Teichoic acid biosynthesis	5/437	41/13584	0.009726527	0.038674526	0.028277624	K03740/K03429/K03367/K14188/K03739	5
map00051	Fructose and mannose metabolism	9/437	112/13584	0.010074323	0.03912586	0.028607625	K01813/K00844/K11645/K01218/K04041/K02377/K01711/K02793/K19355	9
map00531	Glycosaminoglycan degradation	3/437	15/13584	0.011276819	0.042346848	0.030962713	K01205/K12373/K01197	3
map00760	Nicotinate and nicotinamide metabolism	8/437	95/13584	0.011410827	0.042346848	0.030962713	K03742/K03517/K00767/K00278/K08693/K00324/K03787/K12410	8

Sliva

ID	Description	GeneRatio	BgRatio	pvalue	p.adjust	qvalue	geneID	Count
map03070	Bacterial secretion system	11/98	74/13584	4.45E-12	3.25E-10	2.39E-10	K11003/K11004/K02459/K02460/K02458/K02461/K03203/K03198/K03197/K11907/K11891	11
map01503	Cationic antimicrobial peptide (CAMP) resistance	7/98	54/13584	1.10E-07	4.01239E-06	2.95071E-06	K07640/K07662/K19227/K19228/K19229/K19230/K19226	7
map00520	Amino sugar and nucleotide sugar metabolism	6/98	156/13584	0.00091939	0.018350936	0.013495281	K13016/K15856/K13017/K02793/K00884/K00523	6
map05111	Biofilm formation - Vibrio cholerae	5/98	106/13584	0.001005531	0.018350936	0.013495281	K02459/K02460/K02458/K02461/K10941	5

Table S15. Summary of Spearman's correlation between DESeq2-selected genera (clr) and ANCOM-BC2-selected PICRUSt2-predicted functions

Presented as a separate Excel file.

Table S16 Summary of detected ion features of metabolites in saliva, serum and feces

Saliva

mode	All	MS2	HMDB	KEGG	Annotated
negative	5689	513	2342	1980	2839
positive	6672	463	2325	1820	2735

Serum

mode	All	MS2	HMDB	KEGG	Annotated
negative	11152	549	3974	2919	4774
positive	11708	519	4529	3352	5304

Feces

mode	All	MS2	HMDB	KEGG	Annotated
negative	11732	1237	6239	5258	7288
positive	13492	1363	7128	6151	8206

MS2: MS2-identified features

HMDB: HMDB-annotated MS1-identified features

KEGG: KEGG-annotated MS1-identified features

Annotated: Annotated MS1- and MS2-identified features

Table S17. Details of MS2-annotated metabolic features (adducts)

Presented as a separate Excel file.

Table S18. Filtered features with $\log_{10}$-transformed Pareto scaling

Presented as a separate Excel file.

Table S19. Summary of PERMANOVA (Euclidean distances) of metabolome data

Feces

	Df	SumOfSqs	R2	F	Pr(>F)	
Disease	1	3073	0.02835	1.5172	0.036	*
Residual	52	105341	0.97165			
Total	53	108414	1			

Signif. codes: 0 '***' 0.001 '**' 0.01 '*' 0.05 '.' 0.1 ' ' 1

Saliva

	Df	SumOfSqs	R2	F	Pr(>F)
Disease	1	2139	0.02135	1.1344	0.27
Residual	52	98054	0.97865		
Total	53	100193	1		

Serum

	Df	SumOfSqs	R2	F	Pr(>F)
Disease	1	793	0.01745	0.9234	0.519
Residual	52	44667	0.98255		
Total	53	45461	1		

PERMANOVA with 999 permutations

Table S20. limma analysis of metabolic features

Presented as a separate Excel file.

Table S21. The enriched pathways predicted jointly from mummichog and GSEA methods of limma-selected fecal features

Feces

	Total_Size	Hits	Sig_Hits	Mummichog_Pvals	GSEA_Pvals	Combined_Pvals
Purine metabolism	80	4	3	0.05172	0.03448	0.01307
Galactose metabolism	41	17	16	0.03895	0.05556	0.01544
Glycerophospholipid metabolism	156	8	6	0.1098	0.03846	0.02731
Sialic acid metabolism	107	11	10	0.05172	0.08621	0.02859
De novo fatty acid biosynthesis	106	1	1	0.07703	0.06122	0.02998
Di-unsaturated fatty acid beta-oxidation	26	1	1	0.07703	0.06122	0.02998
Fatty acid activation	74	1	1	0.07703	0.06122	0.02998
Heparan sulfate degradation	34	2	2	0.1896	0.06122	0.06333
Keratan sulfate degradation	68	2	2	0.1896	0.06122	0.06333
N-Glycan Degradation	16	3	3	0.1896	0.06122	0.06333
Starch and Sucrose Metabolism	33	10	10	0.06656	0.2407	0.08225
Linoleate metabolism	46	12	4	0.3059	0.05769	0.0889
Ascorbate (Vitamin C) and Aldarate Metabolism	29	8	2	0.4471	0.04348	0.09604
Fatty Acid Metabolism	63	2	1	0.3127	0.07407	0.1104
Tryptophan metabolism	94	6	3	0.3059	0.07692	0.1118
Pyrimidine metabolism	70	6	1	0.8673	0.03448	0.1349
Phosphatidylinositol phosphate metabolism	59	5	4	0.1338	0.2586	0.151
Fructose and mannose metabolism	33	5	5	0.3127	0.1111	0.1514
Porphyrin metabolism	43	2	1	0.6306	0.0566	0.1546
Beta-Alanine metabolism	20	3	2	0.3127	0.1296	0.1704
Glycosylphosphatidylinositol(GPI)-anchor biosynthesis	6	1	1	0.2803	0.1458	0.1715
Hyaluronan Metabolism	8	1	1	0.2803	0.1667	0.1899
Androgen and estrogen biosynthesis and metabolism	95	13	11	0.4319	0.1364	0.2257
Glycolysis and Gluconeogenesis	49	5	4	0.4319	0.1379	0.2276
Glycine, serine, alanine and threonine metabolism	88	4	2	0.5398	0.1207	0.2431
Aspartate and asparagine metabolism	114	12	2	0.9062	0.07692	0.2554
Carnitine shuttle	72	8	3	0.7365	0.1042	0.2738

Xenobiotics metabolism	110	3	2	0.4836	0.1837	0.3039
Propanoate metabolism	31	3	3	0.1896	0.6792	0.3927
Hexose phosphorylation	20	6	6	0.1896	0.6981	0.4
Squalene and cholesterol biosynthesis	55	8	1	0.5689	0.25	0.4196
Aminosugars metabolism	69	3	2	0.4836	0.3061	0.4308
Vitamin D3 (cholecalciferol) metabolism	16	4	2	0.4836	0.3273	0.45
Chondroitin sulfate degradation	37	1	1	0.4836	0.449	0.5487
Glycosphingolipid biosynthesis - ganglioseries	62	2	2	0.4836	0.449	0.5487
Glycosphingolipid biosynthesis - globoseries	16	1	1	0.4836	0.449	0.5487
Glycosphingolipid metabolism	67	4	4	0.4836	0.449	0.5487
N-Glycan biosynthesis	48	2	2	0.4836	0.449	0.5487
Pyruvate Metabolism	20	2	2	0.4836	0.449	0.5487
Lipoate metabolism	8	2	1	0.4836	0.4727	0.5659
C21-steroid hormone biosynthesis and metabolism	112	26	6	0.9007	0.3051	0.6298
Bile acid biosynthesis	82	31	13	0.7181	0.4035	0.6487
Vitamin E metabolism	54	16	1	0.7087	0.4464	0.6805
Tyrosine metabolism	160	10	2	0.7764	0.4902	0.7483
Caffeine metabolism	11	6	2	0.8127	0.6364	0.8582
Pentose phosphate pathway	37	2	1	0.6306	0.8367	0.865
Urea cycle/amino group metabolism	85	5	1	0.8127	0.7069	0.8929

Saliva

	Total_Size	Hits	Sig_Hits	Mummichog_Pvals	GSEA_Pvals	Combined_Pvals
Fructose and mannose metabolism	33	1	1	0.07692	0.01639	0.00968
Arachidonic acid metabolism	95	4	4	0.5385	0.08197	0.1819
Prostaglandin formation from arachidonate	78	2	2	0.5385	0.08197	0.1819
Putative anti-Inflammatory metabolites formation from EPA	27	5	5	0.5385	0.08197	0.1819
Porphyrin metabolism	43	1	1	0.3077	0.1509	0.189
Vitamin E metabolism	54	2	1	0.7063	0.127	0.306

Leukotriene metabolism	92	5	3	0.8238	0.7333	0.9086

Serum

	Total_Size	Hits	Sig_Hits	Mummichog_Pvals	GSEA_Pvals	Combined_Pvals
Porphyrin metabolism	43	4	1	0.3493	0.3091	0.3483
Vitamin A (retinol) metabolism	67	5	1	0.2712	0.4894	0.4008

Table S22. Quantitative metabolite set enrichment analysis of limma-selected fecal features using SMPDB database

	Total Cmpd	Hits	Statistic Q	Expected Q	Raw p	Holm p	FDR	
Tryptophan Metabolism	60	1	20.899	1.8868	0.000511	0.011743	0.005185	Kynurenic acid
Methionine Metabolism	43	1	20.091	1.8868	0.000676	0.01488	0.005185	Spermidine
Spermidine and Spermine Biosynthesis	18	1	20.091	1.8868	0.000676	0.01488	0.005185	Spermidine
Purine Metabolism	74	2	15.232	1.8868	0.001847	0.036932	0.008438	Hypoxanthine;Xanthine
Alpha Linolenic Acid and Linoleic Acid Metabolism	19	1	16.659	1.8868	0.002185	0.041522	0.008438	Linoleic acid
Pyruvate Metabolism	48	1	16.18	1.8868	0.002568	0.046228	0.008438	D-Lactic acid
Pyruvaldehyde Degradation	10	1	16.18	1.8868	0.002568	0.046228	0.008438	D-Lactic acid
Taurine and Hypotaurine Metabolism	12	1	15.623	1.8868	0.003096	0.049527	0.008899	Hypotaurine
Pyrimidine Metabolism	59	2	10.808	1.8868	0.006511	0.097657	0.015236	Uridine; Uracil
Trehalose Degradation	11	1	12.691	1.8868	0.008193	0.1147	0.015236	Trehalose
Citric Acid Cycle	32	1	12.54	1.8868	0.008612	0.1147	0.015236	Citric acid
Transfer of Acetyl Groups into Mitochondria	22	1	12.54	1.8868	0.008612	0.1147	0.015236	Citric acid
Warburg Effect	58	1	12.54	1.8868	0.008612	0.1147	0.015236	Citric acid
Tyrosine Metabolism	72	1	12.02	1.8868	0.010219	0.1147	0.015669	Dopamine
Catecholamine Biosynthesis	20	1	12.02	1.8868	0.010219	0.1147	0.015669	Dopamine
Beta-Alanine Metabolism	34	1	11.211	1.8868	0.013331	0.1147	0.019163	Uracil
Phenylalanine and Tyrosine Metabolism	28	1	10.918	1.8868	0.014676	0.1147	0.019856	L-Phenylalanine
Glutathione Metabolism	21	1	10.092	1.8868	0.01924	0.11544	0.024584	Pyroglutamic acid
Thiamine Metabolism	9	1	9.7564	1.8868	0.021479	0.11544	0.026001	Thiamine
Histidine Metabolism	43	1	9.2525	1.8868	0.025337	0.11544	0.029137	Methylimidazoleacetic acid
Sulfate/Sulfite Metabolism	22	1	6.8326	1.8868	0.056232	0.1687	0.056232	Estrone sulfate
Androgen and Estrogen Metabolism	33	1	6.8326	1.8868	0.056232	0.1687	0.056232	Estrone sulfate
Estrone Metabolism	24	1	6.8326	1.8868	0.056232	0.1687	0.056232	Estrone sulfate

Holm p significant; FDR significant

Table S23. Quantitative metabolite set enrichment analysis of limma-selected fecal features using database of Disease Signatures in Feces

	Total Cmpd	Hits	Statistic Q	Expected Q	Raw p	Holm p	FDR	
Colorectal Cancer	827	23	11.996	1.8868	7.12E-07	1.64E-05	1.64E-05	Linoleic acid; L-Phenylalanine; Uridine; Citric acid; Hypoxanthine; Pyroglutamic acid; Xanthine; Uracil; Azelaic acid; Undecanedioic acid; Spermidine; 1,3-Dimethyluric acid; Naringenin; p-Cresol sulfate; 5-(2-Hydroxyethyl)-4-methylthiazole; 2-Hydroxyglutarate; Kynurenic acid; Thiamine; Catechin; Eriodictyol; Luteolin; Oleoylcarnitine; Sinapic acid
Ulcerative Colitis	304	11	14.205	1.8868	4.02E-06	8.84E-05	4.62E-05	L-Phenylalanine; Uridine; Undecanedioic acid; Hypoxanthine; Xanthine; Uracil; Kynurenic acid; Spermidine; Citric acid; Linoleic acid; Pyroglutamic acid
Crohn's Disease	233	7	14.731	1.8868	1.62E-05	0.000341	0.000125	L-Phenylalanine; Hypoxanthine; Xanthine; Uracil; Kynurenic acid; Spermidine; Citric acid
Unclassified Ibd	84	5	14.185	1.8868	0.000134	0.002672	0.000768	Hypoxanthine; L-Phenylalanine; Xanthine; Uracil; Spermidine;
Ileal Crohn's Disease	19	2	13.705	1.8868	0.000313	0.005947	0.00144	L-Phenylalanine; Linoleic acid;
Enthesitis-related Arthritis	23	1	20.899	1.8868	0.000511	0.00919	0.001957	Kynurenic acid
Irritable Bowel Syndrome	93	5	11.304	1.8868	0.000628	0.01067	0.002062	Hypoxanthine; Pyroglutamic acid; L-Phenylalanine; Uridine; Citric acid;
Cryptosporidium Infection	29	3	11.174	1.8868	0.001889	0.03023	0.003865	L-Phenylalanine; Pyroglutamic acid; Trehalose;
Bladder Infections	6	1	16.66	1.8868	0.002185	0.032771	0.003865	Linoleic acid
Ccd	17	1	16.66	1.8868	0.002185	0.032771	0.003865	Linoleic acid
Cirrhosis	12	1	16.66	1.8868	0.002185	0.032771	0.003865	Linoleic acid
Interstitial Cystitis	6	1	16.66	1.8868	0.002185	0.032771	0.003865	Linoleic acid
Liver Cirrhosis	12	1	16.66	1.8868	0.002185	0.032771	0.003865	Linoleic acid
Ankylosing Spondylitis	21	2	11.423	1.8868	0.003631	0.036313	0.005568	Hypoxanthine; L-Phenylalanine

Rheumatoid Arthritis	21	2	11.423	1.8868	0.003631	0.036313	0.005568	Hypoxanthine; L-Phenylalanine
Myalgic Encephalomyelitis/chronic Fatigue Syndrome	22	2	11.011	1.8868	0.006223	0.049786	0.008946	L-Phenylalanine; Uracil
Iron Deficiency	25	1	11.253	1.8868	0.013149	0.092041	0.01533	Undecanedioic acid
Asymptomatic Diverticulosis	22	1	11.211	1.8868	0.013331	0.092041	0.01533	Uracil
Diverticular Disease	22	1	11.211	1.8868	0.013331	0.092041	0.01533	Uracil
Symptomatic Uncomplicated Diverticular Disease	22	1	11.211	1.8868	0.013331	0.092041	0.01533	Uracil
Autism	102	1	10.918	1.8868	0.014676	0.092041	0.015343	L-Phenylalanine
Gout	14	1	10.918	1.8868	0.014676	0.092041	0.015343	L-Phenylalanine
Inflammatory Bowel Disease	17	1	8.699	1.8868	0.030383	0.092041	0.030383	1,3-Dimethyluric acid

Holm p significant; FDR significant

Table S24. GLM analysis of metabolic features

Presented as a separate Excel file.

Table S25. sPLS-DA analysis of metabolic features

Presented as a separate Excel file.

Table S26. The enriched pathways predicted jointly from mummichog and GSEA methods of sPLS-DA-selected fecal features

Feces

	Total_Size	Hits	Sig_Hits	Mummichog_Pvals	GSEA_Pvals	Combined_Pvals
Linoleate metabolism	46	4	4	0.08622	0.03846	0.02225
De novo fatty acid biosynthesis	106	1	1	0.1981	0.02	0.02587
Di-unsaturated fatty acid beta-oxidation	26	1	1	0.1981	0.02	0.02587
Fatty acid activation	74	1	1	0.1981	0.02	0.02587
Vitamin E metabolism	54	2	1	0.234	0.01852	0.02792
Glycerophospholipid metabolism	156	7	6	0.2471	0.02	0.03118
Galactose metabolism	41	16	16	0.1406	0.05882	0.04792
Xenobiotics metabolism	110	2	2	0.4483	0.01887	0.04883
Sialic acid metabolism	107	10	10	0.1224	0.07547	0.05252
Fatty Acid Metabolism	63	2	1	0.4218	0.05769	0.1148
Purine metabolism	80	4	3	0.2449	0.125	0.1374
Tryptophan metabolism	94	6	3	0.6884	0.07843	0.2116
Fructose and mannose metabolism	33	5	5	0.6095	0.09259	0.2187
Heparan sulfate degradation	34	2	2	0.4218	0.1346	0.2196
Keratan sulfate degradation	68	2	2	0.4218	0.1346	0.2196
N-Glycan Degradation	16	3	3	0.4218	0.1346	0.2196
Phosphatidylinositol phosphate metabolism	59	4	4	0.234	0.2778	0.2427
Carnitine shuttle	72	5	3	0.6985	0.09615	0.2485
Porphyrin metabolism	43	1	1	0.4483	0.1633	0.2646
Beta-Alanine metabolism	20	3	2	0.6095	0.1296	0.2795
Squalene and cholesterol biosynthesis	55	8	1	0.5579	0.1522	0.2943
Starch and Sucrose Metabolism	33	10	10	0.234	0.3704	0.2987
Androgen and estrogen biosynthesis and metabolism	95	12	11	0.6095	0.1667	0.334
Glycosylphosphatidylinositol(GPI)-anchor biosynthesis	6	1	1	0.4483	0.2642	0.3711
C21-steroid hormone biosynthesis and metabolism	112	16	6	0.7439	0.1739	0.3939

Glycolysis and Gluconeogenesis	49	5	4	0.7483	0.1887	0.4176
Hyaluronan Metabolism	8	1	1	0.4483	0.3396	0.4388
Lipoate metabolism	8	2	1	0.6985	0.25	0.4794
Bile acid biosynthesis	82	31	13	0.7876	0.24	0.5039
Pyrimidine metabolism	70	6	1	0.9757	0.2609	0.6028
Ascorbate (Vitamin C) and Aldarate Metabolism	29	7	2	0.643	0.4	0.6065
Propanoate metabolism	31	3	3	0.4218	0.82	0.7131
Hexose phosphorylation	20	6	6	0.4218	0.84	0.7219
Vitamin D3 (cholecalciferol) metabolism	16	4	2	0.6985	0.5385	0.7439
Aspartate and asparagine metabolism	114	11	3	0.8435	0.5	0.7859
Chondroitin sulfate degradation	37	1	1	0.6985	0.62	0.7955
Glycosphingolipid biosynthesis - ganglioseries	62	2	2	0.6985	0.62	0.7955
Glycosphingolipid biosynthesis - globoseries	16	1	1	0.6985	0.62	0.7955
Glycosphingolipid metabolism	67	4	4	0.6985	0.62	0.7955
N-Glycan biosynthesis	48	2	2	0.6985	0.62	0.7955
Pentose phosphate pathway	37	1	1	0.6985	0.62	0.7955
Pyruvate Metabolism	20	2	2	0.6985	0.62	0.7955
Glycine, serine, alanine and threonine metabolism	88	4	2	0.8435	0.5652	0.8299
Aminosugars metabolism	69	3	2	0.6985	0.86	0.9068
Urea cycle/amino group metabolism	85	4	1	0.9126	0.6667	0.9107
Butanoate metabolism	34	2	1	0.6985	0.96	0.9385
Tyrosine metabolism	160	10	2	0.968	0.766	0.9633
Caffeine metabolism	11	4	2	0.9126	0.9259	0.9873

Saliva

	Total_Size	Hits	Sig_Hits	Mummichog_Pvals	GSEA_Pvals	Combined_Pvals
Fructose and mannose metabolism	33	1	1	0.05	0.01887	0.00752
Porphyrin metabolism	43	1	1	0.25	0.07843	0.0967

Arachidonic acid metabolism	95	4	4	0.45	0.0566	0.119
Prostaglandin formation from arachidonate	78	2	2	0.45	0.0566	0.119
Putative anti-Inflammatory metabolites formation from EPA	27	5	5	0.45	0.0566	0.119
Vitamin E metabolism	54	2	1	0.6071	0.1017	0.2337
Leukotriene metabolism	92	5	3	0.728	0.4154	0.6641

Serum

	Total_Size	Hits	Sig_Hits	Mummichog_Pvals	GSEA_Pvals	Combined_Pvals
Vitamin A (retinol) metabolism	67	1	1	0.15	0.08163	0.06615
Porphyrin metabolism	43	3	1	0.4035	0.2642	0.3453

Table S27. Venn plot analysis of the fecal features selected by DESeq2, GLM and sPLS-DA

Presented as a separate Excel file.

Table S28. FDR-adjusted P values of Spearman's correlation between relative abundance of genera (subgingival plaque and saliva) and clinical parameters

Presented as a separate Excel file.

Table S29. FDR-adjusted P values of Spearman's correlation between clr of genera (feces) and clinical parameters

Presented as a separate Excel file.

Table S30. FDR-adjusted P values of Spearman's correlation between relative abundance of genera (feces) and Venn-selected fecal metabolic features

Presented as a separate Excel file.

Table S31. FDR-adjusted P values of Spearman's correlation between clr of genera (feces) and Venn-selected fecal metabolic features

Presented as a separate Excel file.
